# Cytotoxic Scalarane Sesterterpenes from the Sponge *Hyrtios erectus*

**DOI:** 10.3390/md18050253

**Published:** 2020-05-13

**Authors:** Oh-Seok Kwon, Donghwa Kim, Chang-Kwon Kim, Jeongyoon Sun, Chung J. Sim, Dong-Chan Oh, Sang Kook Lee, Ki-Bong Oh, Jongheon Shin

**Affiliations:** 1 Natural Products Research Institute, College of Pharmacy, Seoul National University, San 56-1, Sillim, Gwanak, Seoul 151-742, Korea; ideally225@snu.ac.kr (O.-S.K.); dkim0719@snu.ac.kr (D.K.); chang-kwon.kim@nih.gov (C.-K.K.); dongchanoh@snu.ac.kr (D.-C.O.); sklee61@snu.ac.kr (S.K.L.); 2 Department of Agricultural Biotechnology, College of Agriculture and Life Science, Seoul National University, San 56-1, Sillim, Gwanak, Seoul 151-921, Korea; rhdlfdhkdsns@snu.ac.kr; 3 Department of Biological Sciences, College of Life Science and Nano Technology, Hannam University, 461-6 Jeonmin, Yuseong, Daejeon 305-811, Korea; cjsim@hnu.kr

**Keywords:** sponge, *Hyrtios erectus*, sesterterpenes, scalaranes, antiproliferative

## Abstract

Twelve new sesterterpenes along with eight known sesterterpenes were isolated from the marine sponge *Hyrtios erectus* collected off the coast of Chuuk Island, the Federated State of Micronesia. Based upon a combination of spectroscopic and computational analyses, these compounds were determined to be eight glycine-bearing scalaranes (**1**–**8**), a 3-keto scalarane (**9**), two oxidized-furan-bearing scalaranes (**10** and **11**), and a salmahyrtisane (**12**). Several of these compounds exhibited weak antiproliferation against diverse cancer cell lines as well as moderate anti-angiogenesis activities. The antiproliferative activity of new compound **4** was found to be associated with G0/G1 arrest in the cell cycle.

## 1. Introduction

Marine scalaranes are a well-known class of sesterterpenes that are exclusively distributed in sponges and their predator nudibranch mollusks [1,2]. This carbon skeleton is composed of a 6/6/6/6-tetracyclic fused ring system. Among the wide structural variations in both the ring systems and the substituents, the terminal isoprene unit typically forms a cyclopentane or an equivalent structure, thus establishing a 6/6/6/6/5-pentacyclic system. Other noticeable variations are nor-, homo- and bishomo-scalaranes, which are generated by either removal or incorporation of one or two methyl groups on the terminal framework [3]. Since the first members of this family were identified from *Cacospongia scalaris* in the early 1970s [4,5], scalarane sesterterpenes have been the most frequently encountered sesterterpenes in sponges, and particularly in the order Dictyoceratida [6], and several hundred unique scalarane sesterterpenes have been reported to date [7]. These compounds exhibit diverse bioactivities such as cytotoxic, antimicrobial, anti-inflammatory, anti-HIV, anti-tubercular, and anti-feedant activities [8,9,10]. Overall, scalaranes are widely recognized as a representative class of terpenes from sponges [1,2,8,9,10]. Despite their immense structural diversity, however, the incorporation of heteroatoms is rather rare, and only less than 13 nitrogeneous scalaranes have been reported to date [11,12,13,14,15,16,17,18].

The genus *Hyrtios* (Family Thorectidae) is a chemically interesting group of Dictyoceratida sponges. Widely distributed in tropical oceans, these animals are the prolific sources of structurally unique and biologically active compounds. As comprehensively covered in a recent review, approximately 150 compounds have been isolated from *Hyrtios* sponges [19]. The majority of *Hyrtios* natural products are sesquiterpenes, sesterterpenes, meroterpenes, and indole- and β-carboline -bearing alkaloids [20]. Several of these compounds, in particular, those from the extensively studied *H. erectus*, exhibit various bioactivities, including broad spectrum of cytotoxic and antimicrobial activities [19].

**Chart 1:**
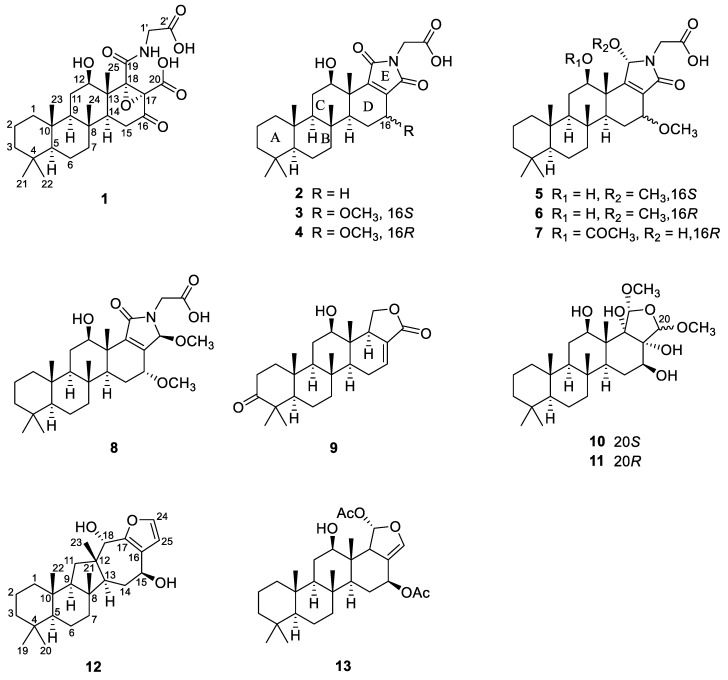
Chemical structures of compounds **1**–**13**.

During the course of our search for bioactive compounds from marine sponges, we encountered specimens of the black encrusting *Hyrtios erectus* off the coast of Chuuk Island, the Federated State of Micronesia. The intriguing LC/ESI-MS profile and significant brine-shrimp lethality (LC_50_ 84 ppm) of the crude extract were indicative of the presence of bioactive compounds, prompting an extensive chemical investigation. Here, we report the isolation of twelve new sesterterpenes along with eight known compounds. Based upon the results of a combination of spectroscopic and computational analyses, the new compounds were determined to be eight glycine-bearing scalaranes (**1**–**8**), one 3-keto scalarane (**9**), two oxidized furan-containing scalaranes (**10** and **11**), and a salmahyrtisane (**12**), thus further contributing to the chemical diversity of *Hyrtios* sponges. These were designated hyrtioscalarins A-H (**1**–**8**), 12-deacetyl-3-oxoscalarin (**9**), 17(*R*),18(*S*)-dihydroxy-19(*R*),20(*S*)-dimethoxysesterstatin 5 (**10**), 17(*R*),18(*S*)-dihydroxy-19(*R*),20(*R*) -dimethoxysesterstatin 5 (**11**), and salmahyrtisol B (**12**), respectively (Chart 1). Several of these compounds exhibited weak antiproliferation against various human cancer cell lines as well as moderate anti-angiogenesis activities. The antiproliferative effect of new compound **4** was found to be associated with G0/G1 arrest in the cell cycle.

## 2. Results and Discussion

### 2.1. Structure Elucidation

The molecular formula of compound **1** was deduced to be C_27_H_39_NO_8_, corresponding to 9 degrees of unsaturation, by HRFABMS analysis ([M-H_2_O-H]^−^ *m/z* 486.2491, calcd for C_27_H_36_NO_7_, 486.2492). The ^13^C NMR data of this compound showed signals indicative of four carbonyl carbons (δ_C_ 204.2, 177.8, 170.4, and 168.7), two oxygenated and non-protonated carbons (δ_C_ 73.3 and 68.7) and one oxymethine carbon (δ_C_ 74.7) (Table 1). The remaining 20 carbons were all aliphatic (four non-protonated, three methine, eight methylene, and five methyl carbons). Therefore, **1** was thought to be a pentacyclic compound. The ^1^H NMR spectra also showed five singlet methyl signals, revealing a terpene or related structure. In conjunction with the mass data and inherent degrees of unsaturation, our preliminary interpretation of the 1-D NMR data suggested that **1** was a highly oxygenated pentacyclic sesterterpene with a nitrogen-containing functionality.

**Figure 1:**
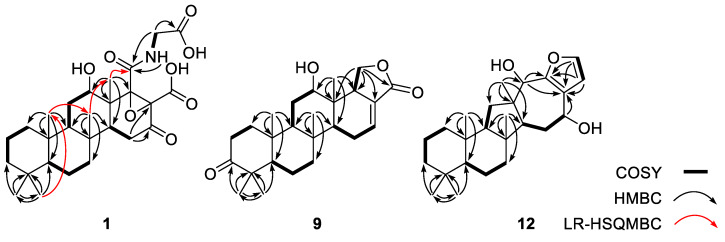
Key correlations of COSY (bold), HMBC (arrows) and LR-HSQMBC (red arrows) experiments for compounds **1**, **9** and **12**.

The planar structure of **1** was determined by combined 2-D NMR analyses (Figure 1). After the initial matching of the protons with their bearing carbons by the HSQC data, three aliphatic proton spin systems were deduced from the ^1^H-^1^H COSY data. These were consisted of three methylenes (C-1-C-3), one methine and two methylenes (C-5-C-7), and two methines and one methylene (C-9, C-11 and C-12), respectively. Subsequently, these linear systems were assembled by using the HMBC correlations with bridgehead singlet methyl protons (H_3_-21-H_3_-25): H_3_-21/C-3, C-4 and C-5, H_3_-22/C-3, C-4 and C-5, H_3_-23/C-1,C-5, C-9, and C-10, H_3_-24/C-7, C-8, C-9, and C-14, and H_3_-25/ C-12, C-13 and C-14. Then these HMBC-based assemblies were confirmed by the LR-HSQMBC correlations between the methyl groups: H_3_-22/C-23, H_3_-23/C-24 and H_3_-24/C-25. Overall **1** was found to possess rings A-C as a 6/6/6 system frequently seen in scalaranes [1,3].

Based on the COSY data, the C-14 methine group was directly connected to a methylene (δ_C_/δ_H_ 35.4/2.35 and 2.29, C-15) that showed no further proton–proton couplings (Figure 1). The HMBC cross peaks of these protons placed a ketone (δ_C_ 204.2, C-16) and a non-protonated carbon (δ_C_ 68.7, C-17) at the neighboring positions: H-14/C-16 and H_2_-15/C-16 and C-17. Similarly, another non-protonated carbon (δ_C_ 73.3, C-18) was placed based on its HMBC cross peaks to previously assigned protons: H-12/C-18 and H_3_-25/C-18. The assignments of C-17 and C-18 were supported by the LR-HSQMBC data [21], a variant of an HMBC experiment, thus constructing a cyclohexanone (ring D): H-14/C-18 and H_2_-15/C-17 (Supporting Information Figure S8). The chemical shifts of these non-protonated carbons at δ_C_ 73.3 and 68.7 indicated not only the attachments of oxygen but also the presence of an epoxide moiety involving these carbons, accounting for the last degree of unsaturation required by the mass data.

The remaining NMR signals were those of an exchangeable NH (δ_H_ 7.88), a methylene (δ_C_/δ_H_ 43.9/4.15 and 3.33) and three carbonyl carbons (δ_C_ 177.8, 170.4 and 168.7). The NH and methylene were directly connected to each other based on their vicinal coupling constants (*J* = 7.4, 3.2 Hz) and COSY data. This spin system was expanded to an *N*-carboglycine moiety by the HMBC cross peaks with the carbonyl carbons at δ_C_ 177.8 and 168.7. A crucial long-range correlation of the latter carbon with H_3_-25 by LR-HSQMBC data linked the *N*-carboglycine moiety to C-18, assigning the carbonyl carbons at C-2’ and C-19, respectively (Figure 1). Although no NMR correlations were observed to the carbonyl carbon at δ_C_ 170.4, this carbon (C-20) must be attached at C-18, the only open position in the main framework. Of the eight oxygens indicated by the mass data of **1**, the four carbonyl groups at C-16, C-19, C-20, and C-2’ and the epoxide at C-17-C-18 only accounted for five. Therefore, the three remaining oxygens must be OH groups at C-12, C-19 and C-20, forming a hydroxyl moiety and two carboxylic acids. Thus, the planar structure of **1** was determined to be a glycine- and epoxide-bearing scalarane sesterterpene.

**Figure 2:**
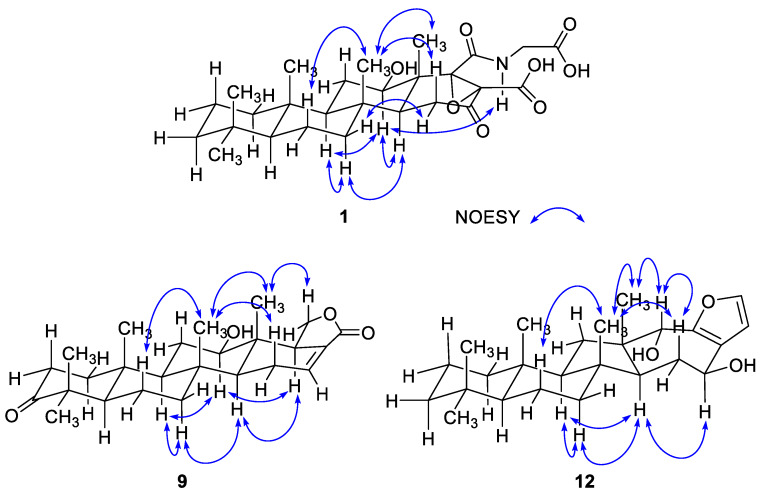
Key correlations of NOESY (blue arrows) experiments for compounds **1**, **9** and **12**.

**Figure 3:**
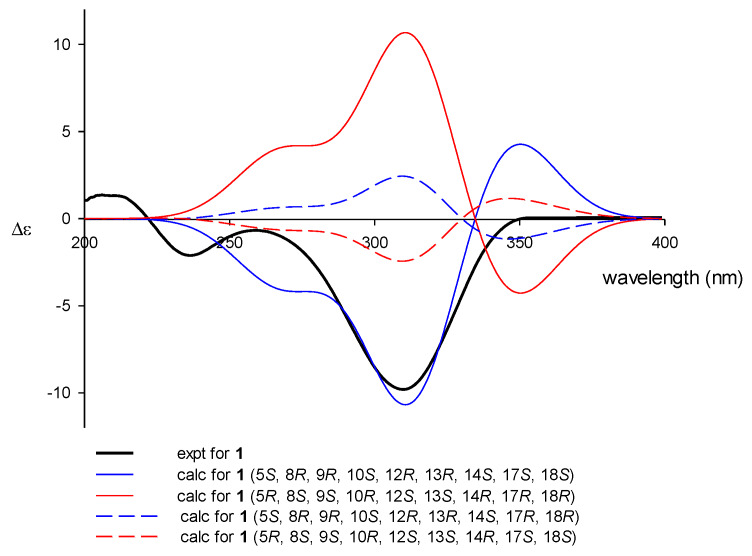
Calculated and experimental ECD spectra of **1**.

The configurations at the stereogenic centers of **1** were assigned based on NOESY data, which readily showed all *trans* junctures for rings A–D, which are typical of scalaranes and similar sesterterpenes (Figure 2). This interpretation was also supported by the characteristic carbon chemical shifts of the bridgehead methines and methyl groups. The β-orientation (12R* configuration) was assigned based on the NOESY cross peak for H-12/H-14 and its vicinal coupling constants (*J* = 11.0, 4.4 Hz) with H_2_-11. For the C-17-C-18 epoxide, which does not have any bound hydrogens, severe steric crowding with the neighboring C-25 methyl group indicated that the olefinic precursor underwent α-oriented attack by the oxygen. This interpretation was confirmed by ECD calculations (Figure 3). Given the all *trans* ring junctures and 12R* configuration, the calculated ECD profile for the 17*S* and 18*S* configurations matched well with the observed profile in both the intensity and wavelength of the signals. In this way, the absolute configurations of the ring junctures and C-12 were also satisfactorily assigned as (5*S*, 8*R*, 9*R*, 10*S*, 12*R*, 13*R*, 14*S*, 17*S*, 18*S*). Thus, compound **1**, designated hyrtioscalarin A, was determined to be a new glycine-bearing scalarane.

The molecular formula of compound **2** was deduced to be C_27_H_39_NO_5_ by HRFABMS analysis ([M-H]^−^ *m/z* 456.2760, calcd for C_27_H_38_NO_5_, 456.2750), which corresponds to 9 degrees of unsaturation. The spectroscopic data of this compound were highly reminiscent of those of **1**, suggesting they shared the same glycine-bearing scalarane core. Detailed examination of the ^1^H and ^13^C NMR and HSQC data, however, revealed remarkable differences, the most noticeable of which were the replacement of the C-16 ketone and C-17-C-18 epoxide of **1** with a methylene group (δ_C_/δ_H_ 23.4/2.53 and 2.26) and two non-protonated carbons (δ_C_ 150.8 and 144.1) in **2** (Table 1). 

The structural differences were defined by a combination of 2-D NMR data (Supporting Information Figure S1). First, the A–C rings of **1** were found to be intact in **2** based on their COSY, HSQC and HMBC data. Then, tracing the proton spin system using the COSY data revealed that the C-14 methine group (δ_C_/δ_H_ 56.4/1.18) was connected to the methylene at C-15 (δ_C_/δ_H_ 17.8/1.98 and 1.67), which was connected in turn to the methylene at C-16 (δ_C_/δ_H_ 23.4/2.53 and 2.26). Subsequently, two non-protonated carbons were placed at C-17 (δ_C_ 144.1) and C-18 (δ_C_ 150.8), constructing ring D as a cyclohexene moiety, based on the HMBC cross peaks of H_2_-15/C-17, H_2_-16/C-17, and H_3_-25/C-18.

The partial formula of the A-D ring portion of **2** was C_23_H_36_O, leaving a C_4_H_3_NO_4_ unit for the remainder of the molecule. The ^1^H NMR and HSQC data of **2** revealed the remaining fragment was an isolated methylene (δ_C_/δ_H_ 40.2/4.19 (2H), C-1’) with HMBC cross peaks with three carbonyl carbons (δ_C_ 175.1, 171.8 and 171.5) (Supporting Information Figure S1). Among these, the carbon at δ_C_ 171.5 was placed at C-20 based on its additional HMBC cross peak with H_2_-16. Therefore, one of the remaining carbonyl carbons must be located at C-19 and attached at C-18, the last open end of the ring system, while the other was a free carboxylic acid carbon at C-2’. The noticeably small and broad shape of the carbon signal at δ_C_ 171.8 in the ^13^C NMR data secured the assignment of this group at C-2’, leaving the signal at δ_C_ 175.1 as C-19. Therefore, **2** must possess a glycine-derived succinimide group (C-17-C-20), fulfilling the remaining 4 degrees of unsaturation required by the mass data. The configurations of stereogenic centers in the A–C rings were also assigned to be identical to those of **1** by the NOESY data (Supporting Information Figure S2) and biogenetic consideration with **1**. Thus, hyrtioscalarin B (**2**) was determined to be a scalarane sesterterpene bearing a succinimide group.

Compounds **3** and **4** were isolated as pale-yellow amorphous solids, and they were found to have the same molecular formula (C_28_H_41_NO_6_) by HRFABMS analyses. The spectroscopic data of these compounds were very similar to each other and highly reminiscent of those of **2**, suggesting they shared the same succinimide-bearing scalarane structure. The most noticeable differences in the ^13^C, ^1^H and HSQC NMR data of **3** and **4** from those of **2** were the replacement of a methylene with a methine (δ_C_/δ_H_ **3**: 73.6/4.18, **4**: 68.4/4.10) and a methoxy group (δ_C_/δ_H_ **3**: 58.1/3.55, **4**: 57.8/3.45) (Supporting Information Tables S1 and S2).

These compounds were defined as 16-methoxy derivatives of **2** based on their combined 1-D and 2-D NMR data, including the crucial HMBC H_3_-OCH_3_/C-16 cross peaks in the spectra of both compounds (Supporting Information Figure S1). The relative configurations at the epimeric C-16 stereogenic center were also determined from the NOESY data. In addition to the same all *trans* ring junctures and 12*R* configurations as seen in **1** and **2** derived from the NOESY data and biogenetic consideration, additional NOESY cross peaks of H-14/H-16, H-15α (δ_H_ 2.32)/H-16, H-15β (δ_H_ 1.65)/H_3_-25, and H-15β/H_3_-24 assigned the 16*S* configuration for **3** (Supporting Information Figure S2). In contrast, the opposite 16*R* configuration was assigned for **4** based on the cross peaks of H-14/H_3_-16OCH_3_, H-15α (δ_H_ 2.12)/H_3_-16OCH_3_ and H-15β/H_3_-25. The absolute configurations of this compound were further supported the ECD calculations (Supporting Information Figure S79). Thus, compounds **3** and **4**, designated hyrtioscalarin C and D, respectively, were defined as epimeric 16-methoxy derivatives of hyrtioscalarin B (**2**).

Compounds **5** and **6** were obtained as another pair of epimeric pale-yellow solids and were found to have the formula C_29_H_45_NO_6_ by HRFABMS analyses. The ^13^C and ^1^H NMR data of these compounds were similar to those of **3** and **4**, indicating they shared the same glycine-incorporated scalarane structures. Aided by the HSQC data, the most conspicuous differences in the ^13^C and ^1^H NMR data were the replacement of a carbonyl carbon with a methoxy and a methine group (δ_C_/δ_H_ **5**: 84.8/5.75 and 50.3/3.09, **6**: 84.5/5.69 and 50.1/3.12) (Supporting Information Tables S1 and S2). The remarkable differentiation of the C-17 and C-18 olefinic carbons between compounds **3**/**4** and **5**/**6** suggested that structural differences were present in compounds **5**/**6** around the amide carbons at C-19 and C-20. This interpretation was confirmed by the COSY and HMBC data of **5**, which assigned a methoxy-methine group at C-19 based on the proton-carbon correlations of H_2_-15/C-17; H-16/C-17; H-19/C-17, C-18 and C-20; H_3_-25/C-18; H_2_-1’/C-19, C-20, and C-2’; H_3_-16OCH_3_/C-16; and H_3_-19OCH_3_/C-19 (Supporting Information Figure S1). Possibly due to the small amounts of materials isolated, the HMBC data of **6** were less informative than those of **5** (Supporting Information Table S4). However, in conjunction with a comparison of their ^13^C and ^1^H NMR data, the planar structure of **6** was defined to be identical to that of **5** based on the key HMBC cross peaks of H_2_-1’/C-19, C-20, and C-2’; H_3_-16OCH_3_/C-16; and H_3_-19OCH_3_/C-19. Overall, these compounds possessed γ-methoxy-α,β-unsaturated butyrolactam rings as a common structural motif (ring E).

Compounds **5** and **6** each possessed two methoxy-bearing stereogenic centers, C-16 and C-19. The relative configurations at these centers were determined from their NOESY data as well as the biogenetic consideration with other scalaranes. For **5**, the 16*S* and 19*S* configurations were assigned based on the cross peaks of H-12/H-14, H-12/H_3_-19OCH_3_, H-14/H-16, H-15β (δ_H_ 1.65)/H_3_-25, H-19/H_3_-25, and H-19/H-1’ (δ_H_ 3.65) (Supporting Information Figure S2). Similarly, the 16*R* and 19*S* configurations were assigned for **6** based on the cross peaks of H-12/H-14, H-12/H_3_-19OCH_3_, H-15α (δ_H_ 2.04)/H_3_-16OCH_3_, H-15β (δ_H_ 1.53)/H_3_-25, H-19/H_3_-25, and H-19/H-1’ (δ_H_ 3.69). These results clearly indicated that **5** and **6** were epimeric at the methoxy-bearing C-16 position. Overall, hyrtioscalarins E (**5**) and F (**6**) are epimeric scalaranes bearing a glycine-derived γ-methoxy-α,β-unsaturated butyrolactam moiety.

The molecular formula of compound **7** was established as C_30_H_45_NO_7_ based on its HRFABMS data ([M-H]^−^ *m/z* 530.3112, calcd for C_30_H_44_NO_7_, 530.3118). The spectroscopic data of this compound were similar to those of **5** and **6**, suggesting an analogous structure. The most noticeable differences in the ^13^C and ^1^H NMR data were the appearance of an acetyl group (δ_C_/δ_H_ 173.4 and 22.2/2.03) and the lack of a methoxy group (Supporting Information Tables S1 and S2). Based upon the extensive 2-D NMR analyses, the structural differences were readily found to be the 12-acetyl and 19-hydroxy groups (Supporting Information Figure S1). After assigning the same ring junctures and 12*R* configurations using the NOESY data and biogenetic consideration, the 16*R* and 19*S* configurations were also assigned in a similar manner based on the cross peaks of H-12/H-14, H-14/H_3_-16OCH_3_, H-15α (δ_H_ 2.09)/H_3_-16OCH_3_, H-15β (δ_H_ 1.71)/H_3_-25, H-19/H_3_-25, H-19/H-1’ (δ_H_ 3.88), and H-19/H_3_-12OCOCH_3_ (Supporting Information Figure S2). Thus, hyrtioscalarin G (**7**) was defined as a new scalarane bearing a γ-hydroxy-α,β-unsaturated butyrolactam group.

The molecular formula of compound **8** was established to be C_29_H_45_NO_6_, identical to that of **5** and **6**, by HRFABMS analysis ([M-H]^−^ *m/z* 502.3169, calcd for C_29_H_44_NO_6_, 502.3169). Although the spectroscopic data of this compound were also very similar to those of **5** and **6**, a detailed examination of its ^13^C and ^1^H NMR data revealed significant differences among the carbons and protons of the butyrolactam (ring E) and the adjacent positions (Supporting Information Tables S1 and S2). Although the 2-D NMR data indicated the same A–D rings as in the other compounds, the butyrolactam moiety of **8** was revealed to have a distinct substitution mode. That is, key HMBC cross peaks were found for H_2_-15/C-17; H-16/C-17, C-18 and C-19; H_2_-1’/C-19, C-20 and C-2’; and H_3_-20OCH_3_/C-19 (Supporting Information Figure S1). Therefore, methylation must have occurred at the C-20 lactam carbon instead of C-19. After assigning the all *trans* ring junctures and 12*R* configurations from the NOESY data and biogenetic consideration, the 16*R* and 20*S* configurations of the methoxy-bearing stereogenic centers were also determined from the NOESY cross peaks of H-15α (δ_H_ 2.16)/H_3_-16OCH_3_, H-15β (δ_H_ 1.71)/H_3_-25, H-16/H_3_-20OCH_3_, H-20/H_3_-16OCH_3_, and H_2_-1’ (δ_H_ 3.87)/H_3_-20OCH_3_ (Supporting Information Figure S2). This interpretation was further supported by the ECD calculation (Supporting Information Figure S79). In this way, compound **8**, designated hyrtioscalarin H, was determined to be a new butyrolactam-bearing scalarane.

Hyrtioscalarins A-H (**1**–**8**) all possess glycine-derived nitrogenous functionalities. A literature study showed that among the numerous scalarane sesterterpenes reported to date, few are nitrogenous. To the best of our knowledge, the only examples are molliorins A–C from *Cacospongia mollior* [11,12,13], an unnamed compound (**14**, a congener in this work) from *Hyatella* sp. [14], petrosaspongiolactams A–C from *Petrosaspongia* sp. [15], scalalactam A–D from *Spongia* sp. [16], a pyrrole containing scalarane from *Scalarispongia* sp. [17], and 24-methoxypetrosaspongia C from *Hyrtios erectus* [18]. Moreover, only the one compound from *Hyatella* sp. possesses the same glycine-derived moiety as seen in **1**–**8**, emphasizing the unusual structural features of these new compounds. Indeed, our finding of diverse nitrogenous derivatives suggests that these compounds are important structural variants of scalaranes.

In addition to glycine-bearing scalaranes **1**–**8**, a number of non-nitrogenous analogs were also isolated. The molecular formula of compound **9** was deduced to be C_25_H_36_O_4_, corresponding to 8 degrees of unsaturation, from its HRFABMS data ([M-H]^−^ *m/z* 399.2531, calcd for C_25_H_35_O_4_, 399.2535). The combined ^13^C, ^1^H and HSQC NMR data of this compound showed the presence of two carbonyl carbons (δ_C_ 220.3 and 172.4) and two olefinic carbons (δ_C_/δ_H_ 137.2/6.81 and 128.6) in the deshielded region, which together account for 3 degrees of unsaturation (Table 2). Signals for an oxymethine (δ_C_/δ_H_ 81.4/3.41) and an oxymethylene (δ_C_/δ_H_ 70.8/4.50 and 4.20) were also conspicuous in the NMR data. Other signals in the shielded region of the ^13^C NMR spectra were those of four non-protonated, four methine, six methylene, and five methyl carbons, which matched well with the corresponding protons based on the HSQC data. Altogether, these data indicated that **9** is pentacyclic and likely to be a typical scalarane sesterterpene.

The planar structure of **9** was elucidated from its 2-D NMR data (Figure 1). First, the COSY data revealed the presence of a spin system involving two methylenes (δ_C_/δ_H_ 40.2/1.98 and 1.58 and 34.8/2.53 and 2.47, C-1 and C-2) and requiring a non-protonated carbon at C-3. Based on the HMBC data, a ketone carbon (δ_C_ 220.3) was placed at this position based on its correlations with neighboring protons at H_2_-1, H_2_-2, H_3_-21, and H_3_-22. Similarly, the A–C ring portion was defined as the same 6/6/6 tricyclic moiety seen in **1**-**8** based on the COSY and HMBC data, including crucial long-range correlations between the five bridgehead methyl protons and their neighboring carbons. Similar to other scalaranes, the chemical shifts (δ_C_/δ_H_ 81.4/3.41), vicinal coupling constants (*J* = 11.4, 3.4 Hz) and HMBC cross peaks to the C-25 methyl group (H-12/C-25, H_3_-25/C-12, H-9/C-12, and H_2_-11/C-12) placed a β-hydroxy group at C-12.

The COSY data located a double bond at C-16 based on the proton–proton correlations of H-14 with H-16 via H_2_-15 (δ_H_ 1.34, 2.39 and 2.27, and 6.81, respectively). Due to the small amount of material obtained, no HMBC data were available to directly support this interpretation. However, the chemical shifts of the C-18 methine group (δ_C_/δ_H_ 51.6/2.92), which showed long-range coupling with H_3_-25, were definitive enough to place an olefinic carbon at C-17 (δ_C_ 128.6). Then, the placement of an oxymethylene (δ_C_/δ_H_ 70.8/4.50 and 4.20) at C-19 was accomplished based on direct proton–proton couplings with H-18. Finally, the HMBC cross peaks of H_2_-19 with the neighboring carbons (H_2_-19/C-13, C-17, C-18, and C-20) not only confirmed its location but also constructed a five-membered lactone as ring E.

The configuration at the newly constructed C-18 center was assigned as *R* based on the NOESY cross peaks of H-18 with the α-oriented H-12 and H-14 protons as well as the all *trans* ring junctures derived from the NOESY data and biogenetic relation with other scalaranes (Figure 2). Thus, the structure of **9**, designated 12-deacetyl-3-oxoscalarin (following the previously reported analog scalarin), was determined to be a scalarane lactone bearing a 3-keto group. A literature study showed that scalarane sesterterpenes possessing a 3-keto group are quite rare. To the best of our knowledge, the only four reported examples are two scalaranes from *H. erectus* [22] and *H. erecta* [23], a norscalarane from the mollusk *Dorisprismatica* (= *Glossodoris*) *atromarginata* [24] and a scalarane with acetoxy group at C-3 position from *H. erecta* [25].

An oxidized-furan-bearing scalarane (**10**) was isolated as a pale-yellow solid, and it was found to have the molecular formula C_27_H_46_O_7_, corresponding to 5 degrees of unsaturation, by HRFABMS ([M-H]^−^ *m/z* 481.3168, calcd for C_27_H_45_O_7_, 481.3165). With the aid of the HSQC data, the ^13^C and ^1^H NMR spectra of this compound showed signals for two oxygen-bearing non-protonated carbons (δ_C_ 83.1 and 81.0), four methines (δ_C_/δ_H_ 106.1/5.22, 103.2/4.99, 74.4/4.34, and 74.2/3.72) and two methyl groups (δ_C_/δ_H_ 56.0/3.56 and 55.6/3.40) (Supporting Information Tables S1 and S2). Since all the other carbon and proton signals were observed in the shielded regions and showed no evidence of unsaturation, **10** was thought to be a pentacyclic sesterterpene.

**Figure 4:**
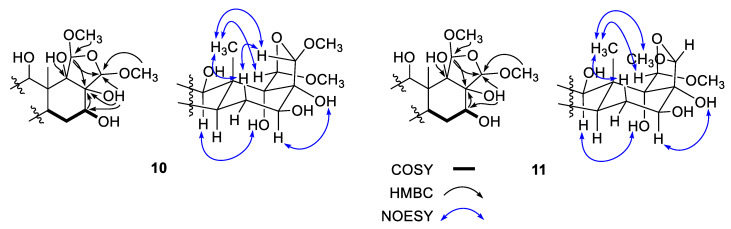
Key correlations of COSY (bold), HMBC (arrows), and NOESY (blue arrows) experiments for compounds **10** and **11**.

Based upon the results of 2-D NMR analyses and comparison with other scalaranes, **10** was readily found to possess the same A-C rings, including the 12-βOH group seen in the compounds described above, and most of the oxygenated functionalities of **10** were located on rings D and E. The HMBC data, including the correlations of hydroxy protons, were crucial to defining the remaining structure (Figure 4). That is, starting at H-14 (δ_H_ 1.32), the COSY correlations placed a hydroxy methine at C-16 (δ_C_/δ_H_ 74.2/3.72), which was confirmed by the HMBC cross peaks of this methine carbon with H-14 and H_2_-15 (δ_H_ 1.79 and 1.44). The neighboring carbon, C-17 (δ_C_ 81.0), was similarly assigned as being a hydroxy-bearing non-protonated carbon based on the correlations of H_2_-15/C-17, H-16/C-17, and 17-OH (δ_H_ 2.32)/C-16 and C-17. Another hydroxy group was also placed at C-18 (δ_C_ 83.1) by the HMBC correlations of H_3_-25/C-18 and of 18-OH (δ_H_ 3.87)/C-13 and C-18.

In addition, two methoxy groups (δ_C_/δ_H_ 55.6/3.40 and 56.0/3.56) were directly attached to oxymethines (δ_C_/δ_H_ 106.1/5.22 and 103.2/4.99, C-19 and C-20) by diagnostic 3-bond HMBC interactions. The deshielded chemical shifts of these oxymethines in conjunction with the remaining oxygen from the mass data indicated these were part of an acetal. This interpretation was confirmed, and a tetrahydrofuran moiety was constructed by a series of HMBC cross peaks, namely, H-19/C-13, C-17 and C-20, H-20/C-17, and 18-OH (δ_H_ 3.47)/C-20. Thus, the planar structure of **10** was defined as a polyoxygenated scalarane.

Compound **10** possessed stereogenic centers at the D/E ring juncture and adjacent sites, and their relative configurations were assigned based on the NOESY data (Figure 4). The H-14/H-16 cross peak defined a β-orientation for the 16-OH group, which was in good agreement with the vicinal coupling constants (*J* = 12.0, 5.7 Hz) of H-16. In addition, the H_3_-25 protons showed cross peaks with H-15β (δ_H_ 1.44), H-19 and H-20. To achieve sufficient spatial proximity with the bridgehead H_3_-25 protons, not only a *cis* D/E ring juncture but also β-orientations of both H-19 and H-20 in ring E are required. Overall, aided by the biogenetic consideration, the configurations of the stereogenic centers were assigned as 12*R*, 16*S*, 17*R*, 18*S*, 19*R*, and 20*S*. Thus, 17(*R*),18(*S*)-dihydroxy-19(*R*),20(*S*)-dimethoxysesterstatin 5 (**10**), was determined to be a tetrahydrofuran-bearing scalarane sesterterpene.

The molecular formula of compound **11** was deduced to be C_27_H_46_O_7_, identical to that of **10**, based on HRFABMS analysis ([M-H]^−^ *m/z* 481.3172, calcd for C_27_H_45_O_7_, 481.3165). The spectroscopic data of this compound were also very similar to those of **10**, implying the same planar structure, which was confirmed by 1-D and 2-D NMR analyses. However, a detailed comparison of its ^13^C and ^1^H NMR data revealed remarkable differences among the carbons and protons of ring E and the adjacent positions, suggesting that **10** and **11** are epimers (Supporting Information Tables S1 and S2). To confirm this, the NOESY data of **11** showed a cross peak for H-14/H-16, indicating the presence of the same 16β-OH group as in **10**. Additionally, cross peaks of H_3_-25 with H-15β (δ_H_ 1.65), H-19 and H_3_-20OCH_3_ were observed. To generate these signals, the D/E ring juncture must be *cis*, identical to that in **10**, but an opposite orientation was required at the C-20 stereogenic center, indicating a 20*R* configuration (Figure 4). Thus, 17(*R*),18(*S*)-dihydroxy-19(*R*),20(*R*)-dimethoxysesterstatin 5 (**11**) was defined as another tetrahydrofuran-bearing scalarane.

In addition to scalaranes, the *H. erectus* extract contained a sesterterpene with a different skeleton. The molecular formula of compound **12** was established as C_25_H_38_O_3_, corresponding to 7 degrees of unsaturation, by HRFABMS analysis ([M-H]^−^ *m/z* 385.2738, calcd for C_25_H_37_O_3_, 385.2743). The ^13^C NMR data of this compound showed four olefinic carbons (δ_C_ 150.2, 141.4, 124.2, and 111.5) (Table 2). The chemical shifts (δ_H_ 7.30 and 6.50) and coupling constants (*J* = 1.7 Hz for both) of the corresponding protons in the ^1^H NMR data were indicative of a disubstituted furan moiety. Aided by the HSQC data, two oxymethines (δ_C_/δ_H_ 76.1/4.46 and 69.8/4.66) were also identified from the NMR data. The other signals were those of four non-protonated, three methine, seven methylene and five methyl groups. Accordingly, **12** must be a pentacyclic sesterterpene.

A detailed comparison of the 1-D NMR data with those of other compounds revealed remarkable differences in the chemical shifts of several carbons and protons, prompting extensive 2-D NMR analyses (Figure 1). First, the COSY data revealed the presence of two linear chains of three protonated carbons (C-1-C-3 and C-5-C-7). Subsequently, several long-range correlations from these carbons to the protons of four singlet methyl groups (H_3_-19, H_3_-20, H_3_-21 and H_3_-22) secured a 6/6 bicyclic moiety (rings A and B), the same as its congeners.

In the process of determining the structures of rings A and B, the C-9 methine group (δ_C_/δ_H_ 61.4/1.30) was confidently assigned based on its HMBC cross peaks with the H_3_-21 and H_3_-22 protons. In contrast to the scalaranes, however, the proton spin system involving H-9 terminated at the C-11 methylene (δ_C_/δ_H_ 34.9/1.89 and 1.37), placing a non-protonated carbon at neighboring C-12. This was confirmed by the HMBC cross peaks of C-11 and C-12 (δ_C_ 43.9) with a common methyl proton (δ_H_ 1.01, H_3_-23). This methyl proton showed an additional HMBC cross peak with a methine group (δ_C_/δ_H_ 49.3/1.82, C-13). Then, a crucial 3-bond correlation with H_3_-21 confirmed the attachment of C-13 to C-8 of ring B, establishing a five-membered ring (C-8, C-9, C-11-C-13, ring C) (Figure 1).

The COSY data revealed a linear spin system of H-13 (δ_H_ 1.82), H_2_-14 (δ_H_ 1.90 and 1.70) and H-15 (δ_H_ 4.66), analogous to the H-14-H-16 spin system in the scalarane congeners. The assignment of an oxygenated methine at C-15 (δ_C_ 69.8) is also reminiscent of several previously reported compounds. This three-carbon unit was then extended by two additional non-protonated carbons (δ_C_ 124.2 and 150.2, C-16 and C-17, respectively) by the HMBC cross peaks of H_2_-14/C-16 and of H-15/C-16 and C-17. Similarly, another hydroxy-bearing methine (δ_C_/δ_H_ 76.1/4.46, C-18) was attached to C-12 by the HMBC cross peaks of H-18/C-12 and C-14 and of H_3_-23/C-18. Then, C-18 was linked to C-17 by the similar correlations of H-18 with C-16 and C-17, establishing a seven-membered ring (C-12-C-18, ring D).

The preliminary examination of the ^13^C and ^1^H NMR data revealed the presence of a disubstituted furan moiety that required the linkage of C-16 and C-17 to the remaining carbons at C-24 (δ_C_/δ_H_ 141.4/7.30) and C-25 (δ_C_/δ_H_ 111.5/6.50). This confirmed by the small proton–proton coupling constants (*J* = 1.7 Hz) between H-24 and H-25 as well as their HMBC cross peaks with C-16 and C-17 (Figure 1). Thus, a 1,2-disubstituted furan moiety was deduced as the final structural motif (ring E). Overall, the planar structure of **12** was determined to be that of a salmahyrtisane-type sesterterpene [25,26,27].

**Figure 5:**
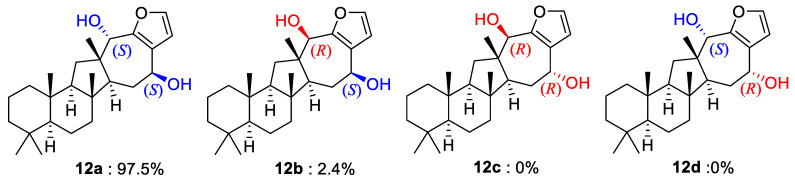
The results of DP4 probability analysis for compound **12**.

In addition to the ring junctures, compound **12** possessed two hydroxy-bearing stereogenic centers at C-15 and C-18. The NOESY data showed several diagnostic 1,3-diaxial cross peaks between the bridgehead methyl protons and spatially adjacent protons, assigning all *trans* ring junctures (Figure 2). The orientations of the hydroxy groups were assigned as 15β and 18α by a number of NOESY cross peaks for protons on ring D and in adjacent positions (H-13/H-15, H-14α (δ_H_ 1.90)/H-15, H-14β (δ_H_ 1.70)/H_3_-21 and 23, and H-18/H_3_-23). Therefore, the 15*S* and 18*S* configurations were assigned. The NOESY-based configurations were further supported by the DP4 calculations in which these configurations (**12a**) matched the NMR data better than did any other configurational combination (**12b**–**12d**) (Figure 5 and Supporting Information Figure S77). Thus, compound **12**, designated sarmahyrtisol B, was elucidated as a new sesterterpene.

The salmahyrtisane skeleton of **12** is rare, and to date, only three compounds with this skeleton have been reported: salmahyrtisol A from the Red Sea sponge *H. erecta* (= *H. erectus*) [25], similan A from a Thai specimen of *H. gumininae* [26], and hippospongide A from a Taiwanese *Hippospongia* sp [27]. The structures of these compounds were closely related to each other by the possession of oxygenated functionalities at C-15 and C-18 on an identical furan-bearing carbon framework. Despite the weak or no cytotoxicity of these compounds, the unusual carbon framework and co-isolation with scalaranes and other sesterterpenes makes the biosynthesis of sarmahyrtisanes of great interest. Accordingly, salmahyrsitol A and hippospongide A were recently prepared by biomimetic synthesis [28]. Notably, **12** has been proposed as a biosynthetic intermediate but was isolated for the first time as a natural product in this work. Both our finding of a sarmahyrtisane from the *Hyrtios* sponge and its co-isolation with scalaranes coincides well with previous works [25,26,27].

Several new compounds, such as **3**/**4**, **5**/**6**, and **10**/**11**, were isolated as configurational pairs at their methoxy-bearing centers. In addition, **10** and **11** showed typical oxidized furans for ring E. These findings suggest that these compounds are abiotic products derived from natural precursors, the results of solvolysis and Michael additions, either during prolonged storage or the isolation process [29]. Even considering this, however, the quantities, diversity and unusual structural features of these new compounds demonstrate both the immense structural variation in sponge sesterterpenes and the chemical prosperity of *Hyrtios* sponges.

In addition to **1**-**12**, the extract of *H. erectus* contained several known scalaranes (Supporting Information Figure S78). Based upon the results of 1-D and 2-D NMR and mass spectroscopy, these were identified as heteronemin (**13**, known from various sponges) [30,31,32], a glycine-bearing derivative (**14**, known from a *Hyatella* sp.) [14], sesterstatins 5 and 6 (**15** and **16**, known from *H. erecta* (= *H. erectus*)) [33,34], 16-hydroxyscalarolide (**17**, known from *H. erectus*) [35], 12-*O*-deacetyl-12-*epi*-scalarin (**18**, known from a *Spongia* sp. [36] and *H. erectus* [37]), and hyrtiosin A and 16-*O*-deacetyl-16-*epi*-scalarolbutenolide (**19** and **20**, known from *H. erecta*) [38,39]. The spectroscopic data of these compounds were in good accordance with those in the literature. Compound **13** was the major constituent, far exceeding all the known and new compounds isolated in this work.

### 2.2. Biological Activity 

The bioactivities of new compounds **1**–**12** and the major metabolite **13** were extensively studied. Anti-angiogenesis activity of compounds **1**–**13** was evaluated in HUVEC cells. Compounds **1**–**12** showed moderate anti-angiogenesis activity without cytotoxicity, while strong anti-angiogenesis activity of **13** was derived from overt cytotoxicity in HUVEC cells. In tests against various human cancer cell lines, compounds **1**–**12** exhibited weak to no antiproliferation activity. Among the scalaranes, **4** and **7** were generally more antiproliferative than other new compounds. In contrast, **13** displayed significant inhibition, comparable to that of etoposide, the positive control, suggesting it may be responsible for the remarkable brine-shrimp lethality of the crude extract (Table 3).

**Figure 6:**
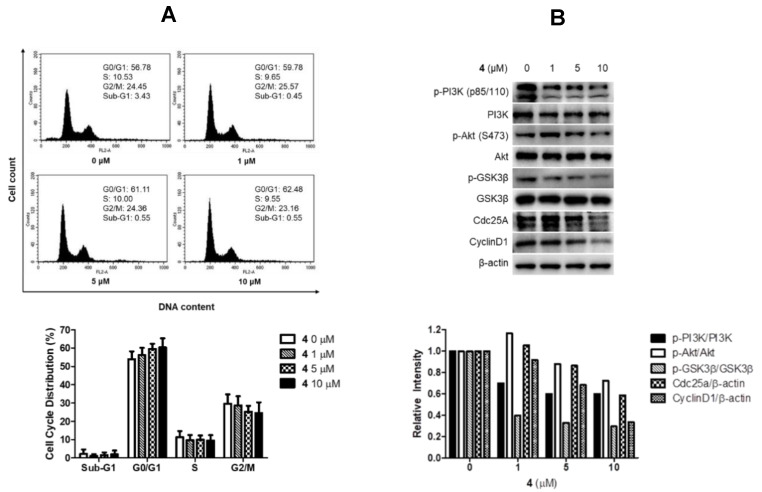
Effect of **4** on the regulation of cell cycle in PC3 cells. (**A**) Cell-cycle regulation measured by flow cytometry. The data are expressed as the mean values ± SD (n = 4) and are representative of four separate experiments; (**B**) Western blotting assay for PI3K/Akt/GSK-3β pathway and G0/G1 cell-cycle regulatory proteins. Relative intensity of indicated proteins was quantified using NIH ImageJ software.

Compound **12**, a salmahyrstane derivative, also showed weak but consistent inhibition toward the cancer strains. The antiproliferative activity of **4** was further studied in PC3 human prostate cancer cells which were insensitive to etoposide treatment. Treatment with **4** slightly increased the G0/G1 cell population (Figure 6A). Therefore, the effects of **4** on proteins that are associated with G0/G1 arrest were further evaluated using Western blot analyses. Consistent with the cell cycle analysis results, treatment of **4** suppressed the expressions of Cdc25A and CyclinD1 in a concentration dependent manner (Figure 6B). Furthermore, treatment of **4** decreased the expressions of p-PI3K, p-Akt, and p-GSK3β without affecting their total protein expression level (Figure 6B). These data suggested that the antiproliferative activities of these compounds would be associated with G0/G1 arrest and PI3K/Akt/GSK-3β pathway. In the anti-angiogenesis assay, several compounds moderately inhibited tube formation at nontoxic concentrations (>10 μM). These compounds also showed weak to no antibacterial activities against various Gram-positive and Gram-negative strains (Supporting Information Table S3). In related assays against microbial enzymes, no compounds significantly inhibited the action of sortase A (SrtA) or isocitrate lyase (ICL) (IC_50_ > 128 g/mL).

In summary, twelve new sesterterpenes along with eight known sesterterpenes were isolated from the tropical sponge *Hyrtios erectus*. Based upon a combination of spectroscopic and computational analyses, these compounds were determined to be eight glycine-bearing scalaranes (**1**–**8**), a 3-keto scalarane (**9**), two oxidized furan-containing scalaranes (**10** and **11**), and a salmahyrtisane derivative (**12**). Several of these compounds exhibited weak antiproliferation against diverse cancer cell lines as well as moderate anti-angiogenesis activities. The antiproliferative activities were found to be associated with G0/G1 arrest in the cell cycle. This work demonstrates both the structural diversity of sponge-derived sesterterpenes and the prosperity of *Hyrtios erectus* as their source.

## 3. Materials and Methods

### 3.1. General Experimental Procedures

Optical rotations were measured using a JASCO P-1020 polarimeter (Jasco, Tokyo, Japan) with a 1-cm cell. CD spectra were obtained using an Applied Photophysics Ltd. Chirascan Plus spectrometer (Leatherhead, Surrey, UK). UV spectra were acquired using a Hitachi U-3010 spectrophotometer (Hitachi High-Technologies, Tokyo, Japan). IR spectra were recorded on a JASCO 4200 FT-IR spectrometer (Jasco, Tokyo, Japan) using a ZnSe cell. NMR spectra were recorded in MeOH-*d*_3_, MeOH-*d*_4_ and CDCl_3_ with the solvent peaks (δ_H_ 3.30/δ_c_ 47.5 and δ_H_ 7.26/δ_c_ 77.0) as internal standards on a Bruker Avance 600 MHz spectrometer (Billerica, MA, USA). Proton and carbon NMR spectra were measured at 600 and 150 MHz, respectively. High-resolution FABMS data were obtained at the National Center for Inter-university Research Facilities (NCIRF), Seoul National University, and acquired using a JEOL JMS 700 mass spectrometer (Jeol, Tokyo, Japan) with 6 keV-energy, emission current 5.0 mA, xenon as the inert gas, and meta-nitrobenzyl alcohol (NBA) as the matrix. HPLC separations were performed on a SpectraSYSTEM p2000 equipped with a refractive index detector (SpectraSYSTEM RI-150 (Waltham, MA, USA)) and a UV-Vis detector (Gilson UV-Vis-151 (Middleton, WI, USA)). All solvents used were of spectroscopic grade or were distilled prior to use.

### 3.2. Animal Material

Specimens of *Hyrtios erectus* sponge (sample number 163CH-111) were collected by hand using SCUBA offshore of Chuuk Island in the Federated States of Micronesia at a depth of 15 m on 7 March 2012. The thick, black encrusting sponge formed finger-like projections that were 3 mm thick and up to 14 cm long. The surface of the sponge retained a distinctly conulose appearance. The specimens also showed a wide variety of encrusting masses with finger-like projections. Sand was present in the skeletal fibers of this sponge, and the fibers were easily broken. The texture of the sponge was quite firm and brittle. These gross morphological features, including the skeletal structures, were consistent with those of *H. erectus* (*Heteronema erecta* Keller, 1889) [40]. A voucher specimen (registry no. spo. 81) was deposited in the MABIK (Marine Biodiversity Institute of Korea), Seocheon, Korea.

### 3.3. Extraction and Isolation

Freshly collected specimens were immediately frozen and stored at −25 °C until use. Lyophilized specimens (225.8 g) were macerated and repeatedly extracted with MeOH (3 × 2 L) and CH_2_Cl_2_ (3 × 2 L). The combined extracts (48.7 g) were sequentially partitioned between H_2_O (33.2 g) and *n*-BuOH (14.7 g); the latter layer was repartitioned between H_2_O-MeOH (15:85, 8.15 g) and *n*-hexane (6.30 g). Then, the H_2_O-MeOH layer was separated by C_18_ reversed-phase flash chromatography using sequential mixtures of MeOH and H_2_O as the eluent (six fractions in an H_2_O-MeOH gradient, from 50:50 to 0:100) followed by acetone and finally EtOAc.

Based on the ^1^H NMR spectra, the fractions eluted with 40:60 H_2_O-MeOH (0.36 g), 20:80 H_2_O-MeOH (0.42 g), 10:90 H_2_O-MeOH (0.56 g) and 0:100 H_2_O-MeOH (1.80 g) were chosen for further separation. The 40:60 H_2_O-MeOH fraction was separated by semipreparative reversed-phase HPLC (YMC-ODS column, 10 × 250 mm; 2.0 mL/min; H_2_O-MeOH, 65:35) to yield compound **1** (*t*_R_ = 44.2 min). This compound was further purified by analytical HPLC (YMC-ODS column, 4.6 × 250 mm; 0.7 mL/min; H_2_O-MeCN gradient (from 90:10 to 40:60 in 40 min); *t*_R_ = 21.1 min).

The 20:80 H_2_O-MeOH fraction was separated by semipreparative reversed-phase HPLC (H_2_O-MeOH, 30:70 with 0.1% TFA), yielding, in order of elution, compounds **9** (*t*_R_ = 8.2 min), **5** (*t*_R_ = 10.1 min), **7** (*t*_R_ = 12.5 min), **6** (*t*_R_ = 16.7 min), **12** (*t*_R_ = 17.9 min), **8** (*t*_R_ = 20.0 min), **3** (*t*_R_ = 20.5 min), **4** (*t*_R_ = 21.9 min), **2** (*t*_R_ = 23.0 min), **10** (*t*_R_ = 35.1 min), and **11** (*t*_R_ = 38.2 min). The former nine compounds were purified by analytical HPLC (H_2_O-MeCN gradient from 70:30 to 0:100 with 0.1% TFA in 50 min); *t*_R_ = 42.9, 44.4, 46.0, 37.0, 40.8, 42.0, 46.6, 47.7, and 48.1 min for **9**, **5**, **7**, **6**, **12**, **8**, **3**, **4**, and **2**, respectively).

The 10:90 H_2_O-MeOH fraction was separated by semipreparative reversed-phase HPLC (H_2_O-MeOH, 25:65), yielding, in order of elution, compounds **16** (*t*_R_ = 18.0 min), **18** (*t*_R_ = 22.8 min), **15** (*t*_R_ = 28.6 min), **20** (*t*_R_ = 38.3 min), **14** (*t*_R_ = 44.1 min), **17** (*t*_R_ = 46.9 min), and **19** (*t*_R_ = 50.4 min). Compounds **13**, **16**–**18**, and **20** were further purified by analytical HPLC (H_2_O-MeCN gradient (from 50:50 to 0:100 in 40 min); *t*_R_ = 29.0, 22.1, 35.5, 34.3, and 30.0 min, for **16**, **18**, **20**, **19**, and **17**, respectively). The MeOH fraction from flash chromatography was also separated by semipreparative reversed-phase HPLC (H_2_O-MeOH, 10:65) to afford compound **13** (*t*_R_ = 23.0 min) as a pure compound. The purified metabolites were isolated in the following amounts: 2.8, 6.1, 7.0, 2.2, 4.6, 1.9, 1.8, 3.0, 0.8, 5.2, 3.7, 2.3, 110, 1.8, 9.8, 0.9, 1.2, 1.6, 4.7, and 2.2 mg for **1**–**20**, respectively.

*Hyrtioscalarin A* (**1**)*:* pale yellow, amorphous solid; [α]${}_{D}^{25}$ −14 (*c* 0.20, MeOH); UV (MeOH) λ_max_ (log ε) 203 (3.77) nm; IR (ZnSe) ν_max_ 3399, 2981, 1718, 1710, 1666, 1397 cm^−1^; ^1^H and ^13^C NMR data, Table 1; HRFABMS *m/z* 486.2491 [M-H_2_O-H]^−^ (calcd for C_27_H_36_NO_7_, 486.2492).

*Hyrtioscalarin B* (**2**)*:* pale yellow, amorphous solid; [α]${}_{D}^{25}$ −5 (*c* 0.25, MeOH); UV (MeOH) λ_max_ (log ε) 207 (3.74); IR (ZnSe) ν_max_ 3525, 2969, 1717, 1701, 1384 cm^−1^; ^1^H and ^13^C NMR data, Table 1; HRFABMS *m/z* 456.2760 [M-H]^−^ (calcd for C_27_H_38_NO_5_, 456.2750).

*Hyrtioscalarin C* (**3**)*:* pale yellow, amorphous solid; [α]${}_{D}^{25}$ −8 (*c* 0.25, MeOH); UV (MeOH) λ_max_ (log ε) 207 (3.74) nm; IR (ZnSe) ν_max_ 3526, 2969, 1717, 1702, 1680, 1395 cm^−1^; ^1^H and ^13^C NMR data, Supporting Information Tables S1 and S2; HRFABMS *m/z* 486.2859 [M-H]^−^ (calcd for C_28_H_41_NO_6_, 486.2856). 

*Hyrtioscalarin D* (**4**)*:* pale yellow, amorphous solid; [α]${}_{D}^{25}$ −21 (*c* 0.10, MeOH); UV (MeOH) λ_max_ (log ε) 206 (3.69) nm; IR (ZnSe) ν_max_ 3525, 2933, 1714, 1701, 1674, 1391 cm^−1^; ^1^H and ^13^C NMR data, Supporting Information Tables S1 and S2; HRFABMS *m/z* 486.2850 [M-H]^−^ (calcd for C_28_H_41_NO_6_, 486.2856). 

*Hyrtioscalarin E* (**5**)*:* pale yellow, amorphous solid; [α]${}_{D}^{25}$ +3 (*c* 0.25, MeOH); UV (MeOH) λ_max_ (log ε) 207 (3.85), 250 (3.05) nm; IR (ZnSe) ν_max_ 3525, 2938, 1720, 1711, 1674, 1316 cm^−1^; ^1^H and ^13^C NMR data, Supporting Information Tables S1 and S2; HRFABMS *m/z* 502.3167 [M-H]^−^ (calcd for C_29_H_44_NO_6_, 502.3169). 

*Hyrtioscalarin F* (**6**)*:* pale yellow, amorphous solid; [α]${}_{D}^{25}$ +2 (*c* 0.20, MeOH); UV (MeOH) λ_max_ (log ε) 206 (3.79), 247 (3.10) nm; IR (ZnSe) ν_max_ 3525, 2939, 1720, 1709, 1678, 1316 cm^−1^; ^1^H and ^13^C NMR data, Supporting Information Tables S1 and S2; HRFABMS *m/z* 502.3169 [M-H]^−^ (calcd for C_29_H_44_NO_6_, 502.3169).

*Hyrtioscalarin G* (**7**)*:* pale yellow, amorphous solid; [α]${}_{D}^{25}$ −9 (*c* 0.30, MeOH); UV (MeOH) λ_max_ (log ε) 207 (3.88), 249 (3.11) nm; IR (ZnSe) ν_max_ 3535, 2939, 1723, 1710, 1678, 1374 cm^−1^; ^1^H and ^13^C NMR data, Supporting Information Tables S1 and S2; HRFABMS *m/z* 530.3112 [M-H]^−^ (calcd for C_30_H_44_NO_7_, 530.3118).

*Hyrtioscalarin H* (**8**)*:* pale yellow, amorphous solid; [α]${}_{D}^{25}$ −4 (*c* 0.25, MeOH); UV (MeOH) λ_max_ (log ε) 206 (3.81), 242 (3.07) nm; IR (ZnSe) ν_max_ 3525, 2968, 1724, 1709, 1680, 1395 cm^−1^; ^1^H and ^13^C NMR data, Supporting Information Tables S1 and S2; HRFABMS *m/z* 502.3169 [M-H]^−^ (calcd for C_29_H_44_NO_6_, 502.3169).

*12-Deacetyl-3-oxoscalarin* (**9**)*:* pale yellow, amorphous solid; [α]${}_{D}^{25}$ −2 (*c* 0.30, MeOH); UV (MeOH) λ_max_ (log ε) 203 (3.68) nm; IR (ZnSe) ν_max_ 3422, 2940, 1719, 1678, 1640, 1399 cm^−1^; ^1^H and ^13^C NMR data, Table 2; HRFABMS *m/z* 399.2531 [M-H]^−^ (calcd for C_25_H_35_O_4_, 399.2535).

*17(R),18(S)-Dihydroxy-19(R),20(S)-dimethoxysesterstatin 5* (**10**)*:* pale yellow, amorphous solid; [α]${}_{D}^{25}$ −9 (*c* 0.25, MeOH); IR (ZnSe) ν_max_ 3524, 2936 cm^−1^; ^1^H and ^13^C NMR data, Supporting Information Tables S1 and S2; HRFABMS *m/z* 481.3168 [M-H]^-^ (calcd for C_27_H_45_O_7_, 481.3165).

*17(R),18(S)-Dihydroxy-19(R),20(R)-dimethoxysesterstatin 5* (**11**)*:* pale yellow, amorphous solid; [α]${}_{D}^{25}$ −22 (*c* 0.30, MeOH); IR (ZnSe) ν_max_ 3522, 2941 cm^−1^; ^1^H and ^13^C NMR data, Supporting Information Tables S1 and S2; HRFABMS *m/z* 481.3172 [M-H]^−^ (calcd for C_27_H_45_O_7_, 481.3165).

*Sarmahyrtisol B* (**12**)*:* pale yellow, amorphous solid; [α]${}_{D}^{25}$ −19 (*c* 0.25, MeOH); UV (MeOH) λ_max_ (log ε) 215 (3.68) nm; IR (ZnSe) ν_max_ 3397, 2927, 1761, 1714, 1463, 1384 cm^−1^; ^1^H and ^13^C NMR data, Table 2; HRFABMS *m/z* 385.2738 [M-H]^−^ (calcd for C_25_H_37_O_3_, 385.2743).

### 3.4. ECD Calculations

All conformational searches were performed using Macromodel (Version 9.9, Schrodinger LLC., NY, USA) software with “mixed torsional/low mode sampling” in the Merck molecular force field (MMFF). The searches were conducted in the gas phase with a 50 kJ/mol energy window limit and a maximum of 10,000 steps to thoroughly examine all low-energy conformers. The Polak-Ribiere conjugate gradient (PRCG) method was utilized for minimization processes with a maximum of 10,000 iterations and a 0.001 kJ (mol Å)^−1^ convergence threshold on the RMS gradient. Conformers within 10 kJ/mol of each global minimum for compound **1** were used for ECD calculations with TmoleX Version 4.2.1 (COSMOlogic GmbH and Co. KG, Leverkusen, Germany) at the B3LYP/6-31G(d,p) level in the gas phase. The CD spectra were simulated by overlapping each transition, where *σ* is the width of the band at 1/e height. ΔE*_i_* and R*_i_* are the excitation energies and rotatory strengths, respectively, for transition *i*. In the current work, the value was 0.10 eV.

$$\Delta \varepsilon \left(E\right)=\frac{1}{2.297\times 10^{-39}}\frac{1}{\sqrt{2\pi \sigma }}\overset{A}{\underset{i}{\sum }}\Delta E_{i}R_{i}e^{\left[-{\left(E-\Delta E_{i}\right)}^{2}/{\left(2\sigma \right)}^{2}\right]}$$

### 3.5. DP4 Probability Method

All conformational searches were performed using the MacroModel (Version 9.9, Schrodinger LLC., NY, USA) program with “Mixed torsional/Low-mode sampling” in the MMFF force field. To thoroughly examine all low-energy conformers, the searches were implemented in the gas phase with a 10 kJ/mol energy window limit and 10,000 as the maximum number of steps. The Polak-Ribiere conjugate gradient (PRCG) method was utilized for minimization processes with 10,000 maximum iterations and a 0.001 kJ (mol Å)^−1^ convergence threshold on the RMS gradient. Conformers within 10 kJ/mol of each global minimum of **12a**, **12b**, **12c**, and **12d** were used for gauge-independent atomic orbital (GIAO) shielding constant calculations without geometry optimization employing TmoleX Version 4.2.1 (COSMOlogic GmbH and Co. KG, Leverkusen, Germany) at the B3LYP/6-31G(d,p) level in the gas phase. Calculated chemical shift values were obtained based on the following equation: $\delta_{\mathrm{calc}.}^{X}=\frac{\sigma^{0}-\sigma^{X}}{1-\sigma^{0}/10^{6}}$ in which $\delta_{\mathrm{calc}.}^{X}$ is the calculated chemical shift value for nucleus x (e.g., ^1^H or ^13^C) and $\sigma^{X}$ and $\sigma^{0}$ are the calculated isotropic constants for nucleus x and tetramethylsilane (TMS), respectively. These calculated chemical shifts of **12a**, **12b**, **12c**, and **12d** were averaged based on their Boltzmann populations and used for calculations of the DP4 analysis by employing an applet available at http://www-jmg.ch.cam.ac.uk/tools/nmr/.

### 3.6. Cell Proliferation Assay and Antibacterial Activity Assay

The antiproliferative activities of the compounds against cancer cells were measured using the sulforhodamine B (SRB) method as described previously [41]. HUVEC growth was assessed by the MTT assay as described previously [42]. Antimicrobial and enzyme-inhibition assays were performed according to a method described previously [43].

### 3.7. Tube Formation Assay

The tube formation assay was conducted as described previously [42]. Briefly, HUVECs in the presence of various concentrations of the test compounds and VEGF (50 ng/mL) were seeded in each well of a Matrigel-coated 96-well plate. The cells were further incubated for 6 h and visualized under an inverted microscope (Olympus Optical Co. Ltd., Tokyo, Japan). The tube formation activity was calculated using the following equation: tube formation (%) = 100 × (average tube number of sample—average tube number of VEGF-control)/(average tube number of VEGF+control—average tube number of VEGF-control). The length of tubular structures was analyzed using the Angiogenesis Image Analyzer in ImageJ software (Bethesda, MD, USA).

### 3.8. Western Blotting

PC3 cells were treated with indicated concentrations of compound **4** for 24 h. Western blotting was conducted as described previously [41].

### 3.9. Cell Cycle Analysis

PC3 cells were seeded and incubated for 24 h and then starved in serum-free medium overnight. The medium was replenished, and compound **4** were treated for 18 h. After the cells were collected and fixed with 70% ethanol, they were stained with propidium iodide (PI) mixed with RNase. The stained cells were analyzed by a FACSCalibur flow cytometry, and the results are presented as histograms using CELLQuest 3.0.1 software (BD Biosciences, CA, USA).

## 4. Conclusions

Twelve new sesterterpenes were isolated and structurally elucidated from the tropical sponge *Hyrtios erectus*. The configurations of stereogenic centers were determined by combined spectroscopic (NOESY) and computational analyses (ECD, DP4). The new compounds exhibited weak antiproliferation against diverse cancer cell lines. In addition, the mechanistic study showed that the antiproliferative activity of compound **4** was related to G0/G1 arrest in cell cycle.

## Figures and Tables

**Table 1:** ^13^C (150 MHz), ^1^H (600 MHz) NMR Assignments for Compounds **1 *^a^*** and **2 *^b^***.

Position	1		2	
	ppm, Type	*δ*, mult (*J* in Hz)	ppm, type	*δ*, mult (*J* in Hz)
1α	41.5, CH_2_	0.86, m	41.4, CH_2_	0.86, m
1β		1.71, br d (12.7)		1.73, br d (12.1)
2α	20.1, CH_2_	1.43, m	20.1, CH_2_	1.44, m
2β		1.66, ddd (13.6, 3.4, 3.4)		1.67, m
3α	43.8, CH_2_	1.18, ddd (13.5, 13.4, 3.8)	43.7, CH_2_	1.16, m
3β		1.37, br d (12.9)		1.38, br d (13.0)
4	34.7, C		34.7, C	
5	58.3, CH	0.85, m	58.3, CH	0.87, m
6α	19.7, CH_2_	1.55, m	19.9, CH_2_	1.61, m
6β		1.41, m		1.48, m
7α	42.9, CH_2_	0.89, m	43.1, CH_2_	0.97, m
7β		1.61, ddd (12.8, 2.9, 2.9)		1.88, ddd (12.6, 2.9, 2.9)
8	38.8, C		39.0, C	
9	59.8, CH	0.96, dd (12.7, 1.2)	59.7, CH	0.95, m
10	38.9, C		39.0, C	
11α	29.0, CH_2_	1.77, ddd (13.1, 4.5, 1.2)	26.8, CH_2_	1.81, ddd (13.1, 4.1, 1.7)
11β		1.54, m		1.51, m
12	74.7, CH	3.96, dd (11.0, 4.4)	76.8, CH	3.71, dd (11.0, 4.5)
13	45.6, C		45.2, C	
14	47.3, CH	1.80, dd (12.9, 4.8)	56.4, CH	1.18, m
15a	35.4, CH_2_	2.29, dd (18.5, 4.8)	17.8, CH_2_	1.98, dd (13.3, 7.1)
15β		2.35, dd (18.5, 13.0)		1.67, m
16α	204.2, C		23.4, CH_2_	2.26, m
16β				2.53, dd (19.8, 5.5)
17	68.7, C		144.1, C	
18	73.3, C		150.8, C	
19	168.7, C		175.1, C	
20	170.4, C		171.5, C	
21	34.3, CH_3_	0.85, s	34.2, CH_3_	0.86, s
22	22.3, CH_3_	0.83, s	22.2, CH_3_	0.84, s
23	17.2, CH_3_	0.87, s	17.1, CH_3_	0.90, s
24	17.6, CH_3_	0.90, s	18.3, CH_3_	0.93, s
25	12.9, CH_3_	1.43, s	17.8, CH_3_	1.15, s
1’	43.9, CH_2_	4.15, dd (17.0, 7.4) 3.33, dd (17.0, 4.3)	40.2, CH_2_	4.19, s
2’	177.8, C		171.8, C	
19-NH		7.88, dd (7.4, 3.2)		

***^a^***^,***b***^ Data were measured at MeOH-*d*_3_ and MeOH-*d*_4_, respectively.

**Table 2:** ^13^C (150 MHz), ^1^H NMR (600 MHz) Assignments for Compounds **9 *^a^*** and **12 *^b^***.

Position	9		12	
	ppm, Type	*δ*, mult (*J* in Hz)	ppm, type	*δ*, mult (*J* in Hz)
1α	40.2, CH_2_	1.50, m	42.8, CH_2_	1.17, ddd (13.6, 13.3, 3.4)
1β		1.98, m		1.37, m
2α	34.8, CH_2_	2.47, m	19.0, CH_2_	1.44, m
2β		2.53, m		1.68, m
3α	220.3, C		40.4, CH_2_	1.12, ddd (12.4, 12.4, 3.7)
3β				1.41, m
4	48.5, C		33.3, C	
5	55.7, CH	1.49, m	58.0, CH	0.93, dd (12.3, 2.4)
6α	20.1, CH_2_	1.62, m	18.6, CH_2_	1.57, m
6β		1.52, m		1.40, m
7α	41.6, CH_2_	1.02, m	40.3, CH_2_	0.98, m
7β		1.81, br d (12.8)		1.71, m
8	38.2, C		45.2, C	
9	58.9, CH	1.06, m	61.4, CH	1.30, dd (13.4, 6.4)
10	37.8, C		37.1, C	
11α	28.5, CH_2_	1.67, m	34.9, CH_2_	1.89, m
11β		1.55, m		1.37, m
12	81.4, CH	3.41, dd (11.4, 3.4)	43.9, C	
13	41.3, C		49.3, CH	1.82, br d (11.3)
14	54.3, CH	1.34, dd (11.3, 5.7)	32.9, CH_2_	1.90, m 1.70, m
15a	24.9, CH_2_	2.39, m	69.8, CH	4.66, dd (10.9, 6.1)
15β		2.27, m		
16	137.2, CH	6.81, dd (6.8, 3.3)	124.2, C	
17	128.6, C		150.2, C	
18	51.6, CH	2.92, m	76.1, CH	4.46, s
19	70.8, CH_2_	4.50, dd (9.5, 9.5)4.20, dd (9.5, 9.5)	33.8, CH_3_	0.85, s
20	172.4, C		21.5, CH_3_	0.84, s
21	27.1, CH_3_	1.07, s	16.8, CH_3_	0.81, s
22	21.3, CH_3_	1.04, s	15.5, CH_3_	0.85, s
23	16.9, CH_3_	0.98, s	25.5, CH_3_	1.01, s
24	16.5, CH_3_	1.00, s	141.4, C	7.30, d (1.7)
25	8.3, CH_3_	0.77, s	111.5, C	6.50, d (1.7)

***^a^***^, ***b***^ Data were measured at MeOH-*d*_4_ and CDCl_3_, respectively.

**Table 3:** Results of Antiproliferation and Anti-Angiogenesis Tests.

Compound	IC_50_ (μM)							
	Antiproliferation *^a^*						Anti-Angiogenesis *^b^*	
	A549	SNU638	HCT116	MDA- MB-231	SK- Hep-1	PC3	Tube Formation	Cell Viability
**1**	>20	>20	>20	>20	>20	>20	5.4	>10
**2**	18	12	18	>20	>20	14	4.9	>10
**3**	>20	>20	>20	>20	>20	>20	7.2	>10
**4**	5.4	5.5	9.7	>20	13	5.9	2.9	>10
**5**	>20	16	19	18	>20	>20	12	>10
**6**	18	19	>20	>20	>20	19	6.2	>10
**7**	6.9	8.9	12	>20	11	5.2	4.3	>10
**8**	>20	>20	16	>20	>20	>20	9	>10
**9**	10	5.8	10	>20	8.1	>20	17	>10
**10**	9.8	5	8.9	>20	18	6.7	13	>10
**11**	12	11	14	17	17	15	14	>10
**12**	7.1	5.5	4.5	7.5	11	7.8	6.9	>10
**13**	0.48	0.3	0.41	0.17	0.46	0.25	0.27	0.59
etoposide	0.76	0.13	0.54	4	0.18	>20		
sunitinib							1.7	6

***^a^*** A549 (human lung cancer cell), SNU638 (human stomach cancer cell), HCT116 (human colon cancer cell), and MDA-MB-231 (human breast cancer cell), SK-Hep1 (human liver cancer cell), and PC3 (human prostate cancer cell); ***^b^*** Measured for PC3 cell line. Tube formation and viability were measured at 6 and 24 h, respectively.

## Supplementary Materials

The following are available online at https://www.mdpi.com/1660-3397/18/5/253/s1, Tables S1–S2: The NMR table of compounds **3**–**8**, **10**, and **11**; Table S3: Results of antibacterial tests; Table S4: Isolated amount of each compound; Figures S1–S76: 1-D and 2-D NMR spectra of **1**-**12**; Figure S77: The DP4 analyses of **12**; Figure S78: Isolated known compounds from *Hyrtios erectus*; Figure S79: Calculated and experimental ECD spectra of **4**, **8** and **13**.
