# Supplementary material for: Cytotoxic Scalarane Sesterterpenes from the Sponge Hyrtios erectus
Source: Mar Drugs. 2020 May 13;18(5):253. doi: 10.3390/md18050253 (PMC7281328; doi:10.3390/md18050253)
Supplement: Supplementary file 1 [file marinedrugs-18-00253-s001.pdf]

Supporting Information

Cytotoxic Scalarane Sesterterpenes from the Sponge *Hyrtios erectus*

Oh-Seok Kwon [1], Donghwa Kim [1], Chang-Kwon Kim [1], Jeongyoon Sun [2], Chung J. Sim [3], Dong-Chan Oh [1], Sang Kook Lee [1], Ki-Bong Oh [2,*], and Jongheon Shin [1,*]

[1] *Natural Products Research Institute, College of Pharmacy, Seoul National University, San 56-1, Sillim, Gwanak, Seoul 151-742, Korea*

[2] *Department of Agricultural Biotechnology, College of Agriculture and Life Science, Seoul National University, San 56-1, Sillim, Gwanak, Seoul 151-921, Korea*

[3] *Department of Biological Sciences, College of Life Science and Nano Technology, Hannam University, 461-6 Jeonmin, Yuseong, Daejeon 305-811, Korea*

shinj@snu.ac.kr

Contents

Table S1.^{13}C NMR (ppm, type) Assignments for Compounds **3-8**, **10**, and **11** at 150 MHz S5
Table S2.^{1}H NMR (δ, mult (J in Hz)) Assignments for Compounds **3-8**, **10**, and **11** at 600 MHz S6
Table S3. Results of Antibacterial Tests S7
Table S4. Isolated amount of each compounds S7
Figure S1. Key correlations of COSY (bold), and HMBC (arrows) experiments for compounds **2-8** S8
Figure S2. Key correlations of NOESY (blue arrows) experiments for compounds **2-8** S9
Figure S3. The ^{1}H NMR (600 MHz, MeOH-d_3) spectrum of **1** S10
Figure S4. The ^{13}C NMR (150 MHz, MeOH-d_3) spectrum of **1** S11
Figure S5. The COSY (600 MHz, MeOH-d_3) spectrum of **1** S12
Figure S6. The eHSQC (600 MHz, MeOH-d_3) spectrum of **1** S13
Figure S7. The HMBC (600 MHz, MeOH-d_3) spectrum of **1** S14
Figure S8. The LR-HSQMBC (600 MHz, MeOH-d_3) spectrum of **1** S15
Figure S9. The NOESY (600 MHz, MeOH-d_3) spectrum of **1** S16
Figure S10. ESI/MS isotopic cluster patterns of compound **1** in positive and negative ion modes S17
Figure S11. The ^{1}H NMR (600 MHz, MeOH-d_4) spectrum of **2** S18
Figure S12. The ^{13}C NMR (150 MHz, MeOH-d_4) spectrum of **2** S19
Figure S13. The COSY (600 MHz, MeOH-d_4) spectrum of **2** S20
Figure S14. The eHSQC (600 MHz, MeOH-d_4) spectrum of **2** S21
Figure S15. The HMBC (600 MHz, MeOH-d_4) spectrum of **2** S22
Figure S16. The NOESY (600 MHz, MeOH-d_4) spectrum of **2** S23
Figure S17. The ^{1}H NMR (600 MHz, $CDCl_3$) spectrum of **3** S24
Figure S18. The ^{13}C NMR (150 MHz, $CDCl_3$) spectrum of **3** S25
Figure S19. The COSY (600 MHz, $CDCl_3$) spectrum of **3** S26
Figure S20. The eHSQC (600 MHz, $CDCl_3$) spectrum of **3** S27
Figure S21. The HMBC (600 MHz, $CDCl_3$) spectrum of **3** S28
Figure S22. The NOESY (600 MHz, $CDCl_3$) spectrum of **3** S29
Figure S23. The ^{1}H NMR (600 MHz, $CDCl_3$) spectrum of **4** S30
Figure S24. The ^{13}C NMR (150 MHz, $CDCl_3$) spectrum of **4** S31
Figure S25. The COSY (600 MHz, $CDCl_3$) spectrum of **4** S32
Figure S26. The eHSQC (600 MHz, $CDCl_3$) spectrum of **4** S33

Figure S27. The HMBC (600 MHz, $CDCl_3$) spectrum of **4** S34
Figure S28. The NOESY (600 MHz, $CDCl_3$) spectrum of **4** S35
Figure S29. The ^{1}H NMR (600 MHz, $CDCl_3$) spectrum of **5** S36
Figure S30. The ^{13}C NMR (150 MHz, $CDCl_3$) spectrum of **5** S37
Figure S31. The COSY (600 MHz, $CDCl_3$) spectrum of **5** S38
Figure S32. The eHSQC (600 MHz, $CDCl_3$) spectrum of **5** S39
Figure S33. The HMBC (600 MHz, $CDCl_3$) spectrum of **5** S40
Figure S34. The NOESY (600 MHz, $CDCl_3$) spectrum of **5** S41
Figure S35. The ^{1}H NMR (600 MHz, $CDCl_3$) spectrum of **6** S42
Figure S36. The ^{13}C NMR (150 MHz, $CDCl_3$) spectrum of **6** S43
Figure S37. The COSY (600 MHz, $CDCl_3$) spectrum of **6** S44
Figure S38. The eHSQC (600 MHz, $CDCl_3$) spectrum of **6** S45
Figure S39. The HMBC (600 MHz, $CDCl_3$) spectrum of **6** S46
Figure S40. The NOESY (600 MHz, $CDCl_3$) spectrum of **6** S47
Figure S41. The ^{1}H NMR (600 MHz, MeOH-d_4) spectrum of **7** S48
Figure S42. The ^{13}C NMR (150 MHz, MeOH-d_4) spectrum of **7** S49
Figure S43. The COSY (600 MHz, MeOH-d_4) spectrum of **7** S50
Figure S44. The eHSQC (600 MHz, MeOH-d_4) spectrum of **7** S51
Figure S45. The HMBC (600 MHz, MeOH-d_4) spectrum of **7** S52
Figure S46. The NOESY (600 MHz, MeOH-d_4) spectrum of **7** S53
Figure S47. The ^{1}H NMR (600 MHz, MeOH-d_4) spectrum of **8** S54
Figure S48. The ^{13}C NMR (150 MHz, MeOH-d_4) spectrum of **8** S55
Figure S49. The COSY (600 MHz, MeOH-d_4) spectrum of **8** S56
Figure S50. The eHSQC (600 MHz, MeOH-d_4) spectrum of **8** S57
Figure S51. The HMBC (600 MHz, MeOH-d_4) spectrum of **8** S58
Figure S52. The NOESY (600 MHz, MeOH-d_4) spectrum of **8** S59
Figure S53. The ^{1}H NMR (600 MHz, MeOH-d_4) spectrum of **9** S60
Figure S54. The ^{13}C NMR (150 MHz, MeOH-d_4) spectrum of **9** S61
Figure S55. The COSY (600 MHz, MeOH-d_4) spectrum of **9** S62
Figure S56. The HSQC (600 MHz, MeOH-d_4) spectrum of **9** S63
Figure S57. The HMBC (600 MHz, MeOH-d_4) spectrum of **9** S64
Figure S58. The NOESY (600 MHz, MeOH-d_4) spectrum of **9** S65
Figure S59. The ^{1}H NMR (600 MHz, $CDCl_3$) spectrum of **10** S66
Figure S60. The ^{13}C NMR (150 MHz, $CDCl_3$) spectrum of **10** S67

Figure S61. The COSY (600 MHz, $CDCl_3$) spectrum of **10** S68
Figure S62. The HSQC (600 MHz, $CDCl_3$) spectrum of **10** S69
Figure S63. The HMBC (600 MHz, $CDCl_3$) spectrum of **10** S70
Figure S64. The NOESY (600 MHz, $CDCl_3$) spectrum of **10** S71
Figure S65. The ^1H NMR (600 MHz, $CDCl_3$) spectrum of **11** S72
Figure S66. The ^{13}C NMR (150 MHz, $CDCl_3$) spectrum of **11** S73
Figure S67. The COSY (600 MHz, $CDCl_3$) spectrum of **11** S74
Figure S68. The HSQC (600 MHz, $CDCl_3$) spectrum of **11** S75
Figure S69. The HMBC (600 MHz, $CDCl_3$) spectrum of **11** S76
Figure S70. The NOESY (600 MHz, $CDCl_3$) spectrum of **11** S77
Figure S71. The ^1H NMR (600 MHz, $CDCl_3$) spectrum of **12** S78
Figure S72. The ^{13}C NMR (150 MHz, $CDCl_3$) spectrum of **12** S79
Figure S73. The COSY (600 MHz, $CDCl_3$) spectrum of **12** S80
Figure S74. The HSQC (600 MHz, $CDCl_3$) spectrum of **12** S81
Figure S75. The HMBC (600 MHz, $CDCl_3$) spectrum of **12** S82
Figure S76. The NOESY (600 MHz, $CDCl_3$) spectrum of **12** S83
Figure S77. The results of DP4 analyses of **12** S84
Figure S78. Isolated known compounds from *Hyrtios erectus* S85
Figure S79. Calculated and experimental ECD spectra of **4**, **8** and **13** S87

Table S1. ^{13}C NMR (ppm, type) Assignments for Compounds **3-8**, **10**, and **11** at 150 MHz

position	**3** [a]	**4** [a]	**5** [a]	**6** [a]	**7** [b]	**8** [b]	**10** [a]	**11** [a]
1	39.6, CH_2	39.6, CH_2	39.8, CH_2	39.8, CH_2	41.4, CH_2	41.4, CH_2	39.7, CH_2	39.8, CH_2
2	18.5, CH_2	18.5, CH_2	18.6, CH_2	18.2, CH_2	20.1, CH_2	20.1, CH_2	18.5, CH_2	18.5, CH_2
3	42.0, CH_2	42.0, CH_2	42.0, CH_2	42.1, CH_2	43.7, CH_2	43.7, CH_2	42.0, CH_2	42.0, CH_2
4	33.2, C	33.2, C	33.2, C	33.3, C	34.7, C	34.7, C	33.2, C	33.3, C
5	56.6, CH	56.3, CH	56.6, CH	56.5, CH	58.5, CH	58.4, CH	56.3, CH	56.4, CH
6	18.1, CH_2	18.1, CH_2	18.1, CH_2	18.6, CH_2	19.8, CH_2	19.8, CH_2	18.2, CH_2	18.2, CH_2
7	41.5, CH_2	41.1, CH_2	41.5, CH_2	41.2, CH_2	43.0, CH_2	43.1, CH_2	41.8, CH_2	41.8, CH_2
8	37.2, C	36.7, C	37.5, C	37.0, C	38.7, C	38.5, C	37.7, C	37.9, C
9	57.7, CH	57.5, CH	58.7, CH	58.6, CH	59.8, CH	59.9, CH	57.6, CH	57.9, CH
10	37.4, C	37.3, C	37.4, C	37.4, C	39.1, C	39.0, C	37.2, C	37.3, C
11	25.4, CH_2	25.3, CH_2	25.5, CH_2	25.6, CH_2	24.5, CH_2	27.0, CH_2	27.2, CH_2	27.4, CH_2
12	74.4, CH	74.1, CH	75.0, CH	79.4, CH	79.0, CH	77.1, CH	74.4, CH	75.0, CH
13	44.1, C	44.3, C	44.7, C	45.3, C	43.9, C	45.1, C	45.4, C	46.1, C
14	53.5, CH	48.9, CH	53.0, CH	48.6, CH	51.4, CH	51.2, CH	49.4, CH	50.0, CH
15	24.3, CH_2	22.6, CH_2	24.4, CH_2	22.6, CH_2	23.7, CH_2	23.0, CH_2	24.9, CH_2	28.0, CH_2
16	73.6, CH	68.4, CH	73.9, CH	68.7, CH	70.8, CH	71.9, CH	74.2, CH	76.6, CH
17	140.4, C	138.7, C	133.9, C	133.6, C	131.7, C	151.8, C	81.0, C	81.8, C
18	152.4, C	152.8, C	160.3, C	160.6, C	165.1, C	147.4, C	83.1, C	82.2, C
19	171.8, C	172.0, C	84.8, CH	84.5, CH	83.0, C	173.3, C	106.1, CH	106.0, CH
20	167.9, C	168.7, C	169.0, C	169.6, C	171.3, C	88.4, C	103.2, CH	110.7, CH
21	33.2, CH_3	33.2, CH_3	33.2, CH_3	33.3, CH_3	34.2, CH_3	34.2, CH_3	33.2, CH_3	33.3, CH_3
22	21.3, CH_3	21.2, CH_3	21.2, CH_3	21.2, CH_3	22.2, CH_3	22.3, CH_3	21.2, CH_3	21.2, CH_3
23	15.9, CH_3	15.9, CH_3	16.2, CH_3	16.1, CH_3	17.2, CH_3	17.1, CH_3	16.3, CH_3	16.4, CH_3
24	17.1, CH_3	17.3, CH_3	17.4, CH_3	17.7, CH_3	18.4, CH_3	18.3, CH_3	18.2, CH_3	18.0, CH_3
25	16.4, CH_3	15.1, CH_3	17.4, CH_3	16.1, CH_3	17.9, CH_3	16.0, CH_3	11.1, CH_3	10.4, CH_3
1’	38.5, CH_2	38.6, CH_2	40.3, CH_2	40.3, CH_2	41.5, CH_2	42.4, CH_2		
2’	170.1, C	170.3, C	171.1, C	171.4, C	173.6, C	172.2, C		
12-OAc					173.4, C			
					22.2, CH_3			
16-OMe	58.1, CH_3	57.8, CH_3	57.3, CH_3	57.3, CH_3	57.9, CH_3	58.0, CH_3		
19-OMe			50.3, CH_3	50.1, CH_3			55.6, CH_3	56.9, CH_3
20-OMe						51.7, CH_3	56.0, CH_3	55.6, CH_3

[a, b]Data were measured at $CDCl_3$, and MeOH-d_4, respectively.

Table S2. ^{1}H NMR (δ, mult (J in Hz)) Assignments for Compounds **3-8, 10**, and **11** at 600 MHz

position	**3** [a]	**4** [a]	**5** [a]	**6** [a]
1α	0.76, m	0.78, m	0.81, m	0.81, m
1β	1.71, br d (12.7)	1.71, br d (12.5)	1.71, br d (12.8)	1.69, br d (12.5)
2α	1.41, m	1.45, m	1.44, m	1.39, m
2β	1.58, m	1.57, m	1.63, m	1.62, m
3α	1.12, m	1.12, m	1.11, m	1.10, m
3β	1.37, br d (12.8)	1.36, br d (13.0)	1.37, br d (13.1)	1.37, br d (13.0)
5	0.78, m	0.81, m	0.80, m	0.82, m
6α	1.60, m	1.61, m	1.58, m	1.57, m
6β	1.42, m	1.41, m	1.39, m	1.43, m
7α	0.88, m	0.96, m	0.90, m	0.96, m
7β	1.82, m	1.76, br d (11.5)	1.84, ddd (12.8, 2.8, 2.8)	1.78, m
9	0.86, m	0.95, m	0.86, m	0.96, m
11α	1.86, m	1.89, br d (11.9)	1.80, dd (12.8, 2.3)	1.80, m
11β	1.52, m	1.52, m	1.55, m	1.54, m
12	3.70, dd (10.5, 3.8)	3.74, br d (9.3)	3.58,dd (11.0, 4.0)	3.64, dd (11.2, 4.1)
14	1.13, m	1.50, m	1.13, m	1.55, m
15α	2.32, dd (12.7, 7.3)	2.12, br d (12.5)	2.25, dd (12.4, 6.8)	2.04, br d (12.0)
15β	1.65, m	1.60, m	1.65, m	1.53, m
16	4.18, dd (9.2, 6.7)	4.10, br d (2.3)	4.21, dd (9.3, 7.2)	3.99, br d (1.7)
19			5.75, s	5.69, s
21	0.84, s	0.84, s	0.84, s	0.82, s
22	0.81, s	0.81, s	0.81, s	0.79, s
23	0.84, s	0.84, s	0.85, s	0.84, s
24	0.91, s	0.89, s	0.91, s	0.88, s
25	1.22, s	1.11, s	1.21, s	1.09, s
1’	4.26, br s	4.27, br s	4.50, d (17.9)	4.52, d (17.9)
			3.65, d (17.9)	3.69, d (17.9)
16-OMe	3.55, s	3.45, s	3.52, s	3.42, s
19-OMe			3.09, s	3.12, s

position	**7** [b]	**8** [b]	**10** [a]	**11** [a]
1α	0.88, m	0.86, m	0.82, m	0.82, m
1β	1.65, m	1.72, m	1.67, m	1.66, m
2α	1.44, m	1.44, m	1.43, m	1.44, m
2β	1.66, m	1.67, m	1.60, m	1.60, m
3α	1.18, ddd (13.8, 13.6, 3.9)	1.17, ddd (13.5, 13.3, 4.2)	1.12, m	1.12, m
3β	1.38, br d (13.6)	1.38, m	1.34, br d (12.4)	1.33, br d (12.4)
5	0.87, m	0.87, m	0.82, m	0.80, m
6α	1.62, m	1.62, m	1.53, m	1.53, m
6β	1.48, m	1.49, m	1.38, m	1.38, m
7α	0.96, m	0.93, m	0.92, br d (12.3)	0.91, br d (12.3)
7β	1.89, ddd (12.6, 3.2, 3.2)	1.87, br d (12.8)	1.78, m	1.77, m
9	1.00, dd (12.6, 1.5)	0.94, m	0.93, m	0.92, m
11α	2.02, dd (4.6, 1.9)	1.78, ddd (13.3, 4.1, 1.9)	1.60, m	1.61, m
11β	1.50, m	1.50, m	1.50, m	1.50, m
12	4.83, dd (11.3, 4.5)	3.62, dd (10.9, 4.3)	4.34, m	4.34, m
14	1.58, dd (12.7, 1.3)	1.37, m	1.32, m	1.26, m
15α	2.09, br d (14.3)	2.16, br d (14.7)	1.79, m	1.74, m
15β	1.71, m	1.71, m	1.44, m	1.65, m
16	3.98, dd (2.7, 1.3)	3.93, dd (4.2, 1.0)	3.72, dd (12.0, 5.7)	3.57, dd (11.5, 6.0)
19	5.40, s		5.22, s	5.40, s
20		5.49, s	4.99, s	5.10, s
21	0.87, s	0.87, s	0.83, s	0.83, s
22	0.84, s	0.84, s	0.79, s	0.80, s
23	0.88, s	0.89, s	0.82, s	0.83, s
24	0.97, s	0.93, s	0.88, s	0.87, s
25	1.22, s	1.09, s	1.00, s	1.12, s
1’	4.27, d (18.0)	4.32, d (18.0)		
	3.88, d (18.0)	3.87, d (18.0)		
12-OAc	2.03, s			
16-OMe	3.41, s	3.41, s		
16-OH			2.32, br s	2.57, br d (10.3)
17-OH			3.47, br s	3.13, br s
18-OH			3.87, br s	3.68, br s
19-OMe			3.40, s	3.49, s
20-OMe		3.09, s	3.56, s	3.46, s

[a, b]Data were measured at $CDCl_3$, and MeOH-d_4, respectively.

Table S3. Results of Antibacterial Tests [a]

	MIC (μg/mL)					
	Gram (+) bacteria			Gram (–) bacteria		
Compound	A	B	C	D	E	F
**1**	>64	8	>64	>64	2	>64
**2**	>64	>64	>64	>64	>64	>64
**3**	>64	>64	>64	64	>64	>64
**4**	>64	>64	>64	64	>64	>64
**5**	>64	>64	>64	>64	>64	>64
**6**	>64	>64	>64	64	>64	>64
**7**	64	>64	>64	64	>64	>64
**8**	64	64	16	32	>64	>64
**9**	>64	>64	>64	64	>64	>64
**10**	>64	>64	>64	>64	>64	>64
**11**	>64	>64	>64	64	>64	>64
**12**	8	8	>64	4	>64	>64
**13**	>64	>64	>64	2	>64	>64
ampicillin	0.13	0.50	0.50		0.13	4
tetracyclin				0.25		

[a]A: *Staphylococcus arueus* (ATCC6538p), B: *Enterococcus faecalis* (ATCC19433), C: *Enterococcus faecium* (ATCC 19434), D: *Klebsiella pneumoniae* (ATCC10031), E: *Salmonella enterica* (ATCC14028), F: *Escherichia coli* (ATCC25922)

Table S4. Isolated amount of each compound

Compound	overall yields (mg)	percent yields (%)	retention time (min)
**1**	2.8	0.0012	44.2[a]
**2**	6.1	0.0027	23.0[b]
**3**	7.0	0.0031	20.5[b]
**4**	2.2	0.00097	21.9[b]
**5**	4.6	0.0020	10.1[b]
**6**	1.9	0.00084	16.7[b]
**7**	1.8	0.00079	12.5[b]
**8**	3.0	0.0013	20.0[b]
**9**	0.8	0.00035	8.2[b]
**10**	5.2	0.0023	35.1[b]
**11**	3.7	0.0016	38.7[b]
**12**	2.3	0.0010	19.7[b]
**13**	110	0.048	23.0[d]
**14**	1.8	0.00079	44.1[c]
**15**	9.8	0.0043	28.6[c]
**16**	0.9	0.00039	18.0[c]
**17**	1.2	0.00053	46.9[c]
**18**	1.6	0.00070	22.8[c]
**19**	4.7	0.0020	50.4[c]
**20**	2.2	0.00097	38.3[c]

semipreparative reversed-phase HPLC (YMC-ODS column, 10 × 250 mm; 2.0 mL/min)

[a] (H_2O-MeOH, 65:35)

[b] (H_2O-MeOH, 30:70 with 0.1% TFA)

[c] (H_2O-MeOH, 25:65)

[d] (H_2O-MeOH, 10:65)

Extract = 225.8 g

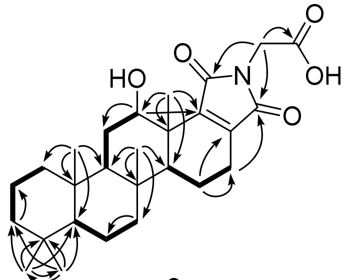

2

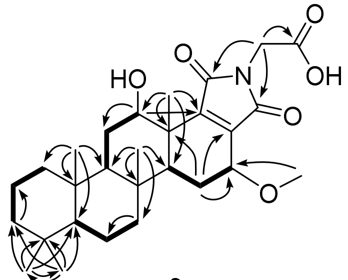

3

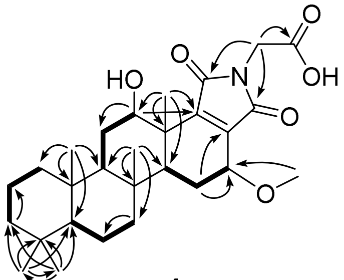

4

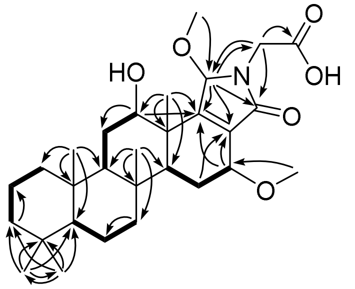

5

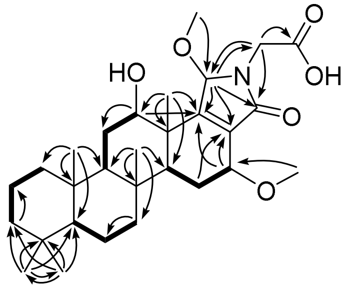

6

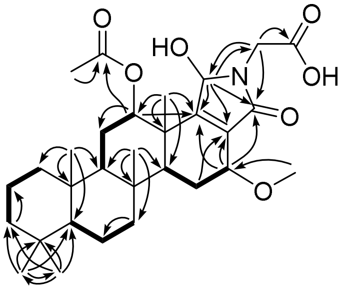

7

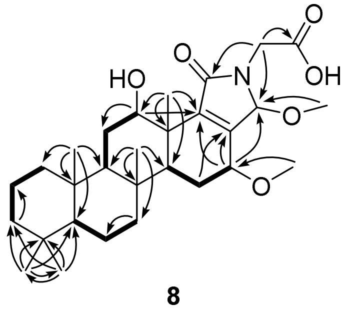

8

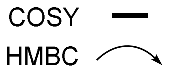

Figure S1. Key correlations of COSY (bold), and HMBC (arrows) experiments for compounds **2-8**

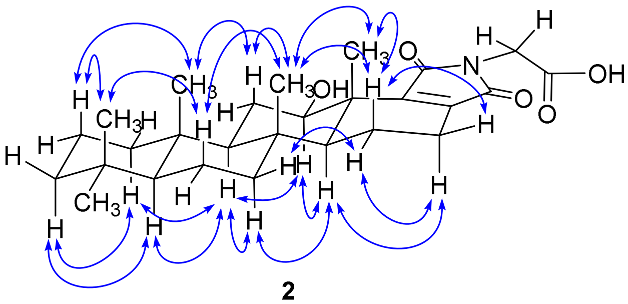

2

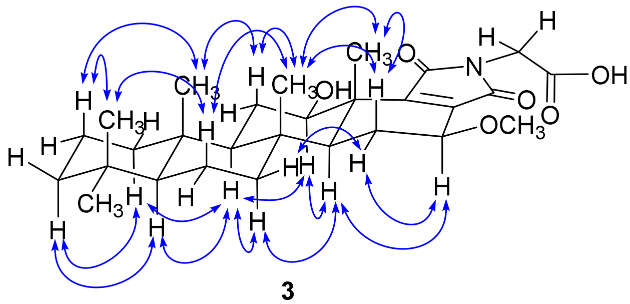

3

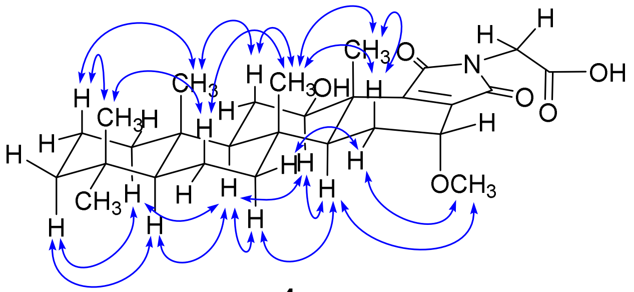

4

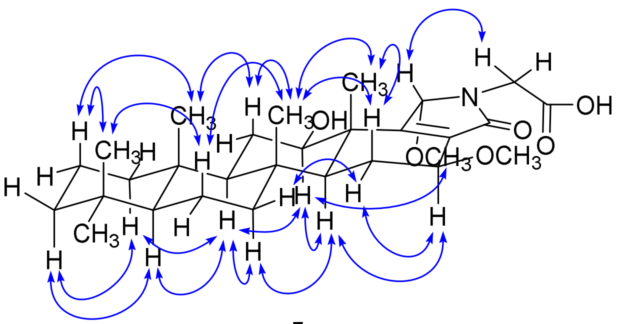

5

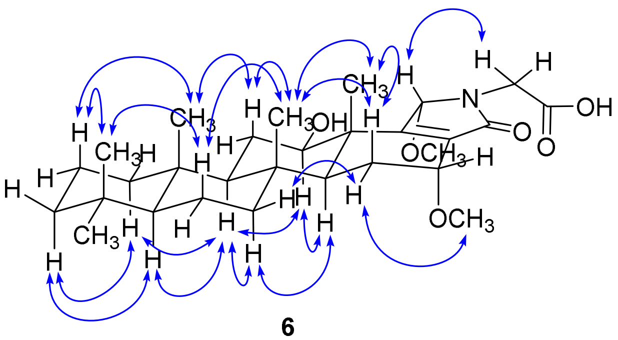

6

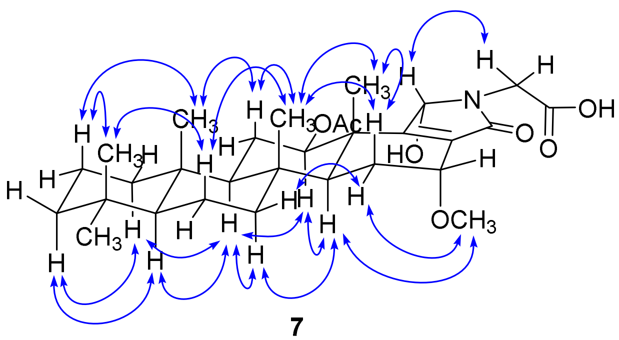

7

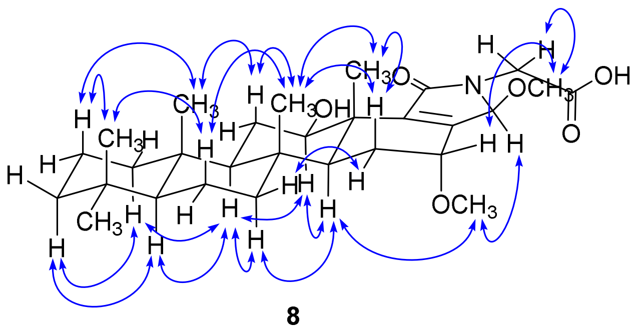

8

NOESY

Figure S2. Key correlations of NOESY (blue arrows) experiments for compounds **2-8**

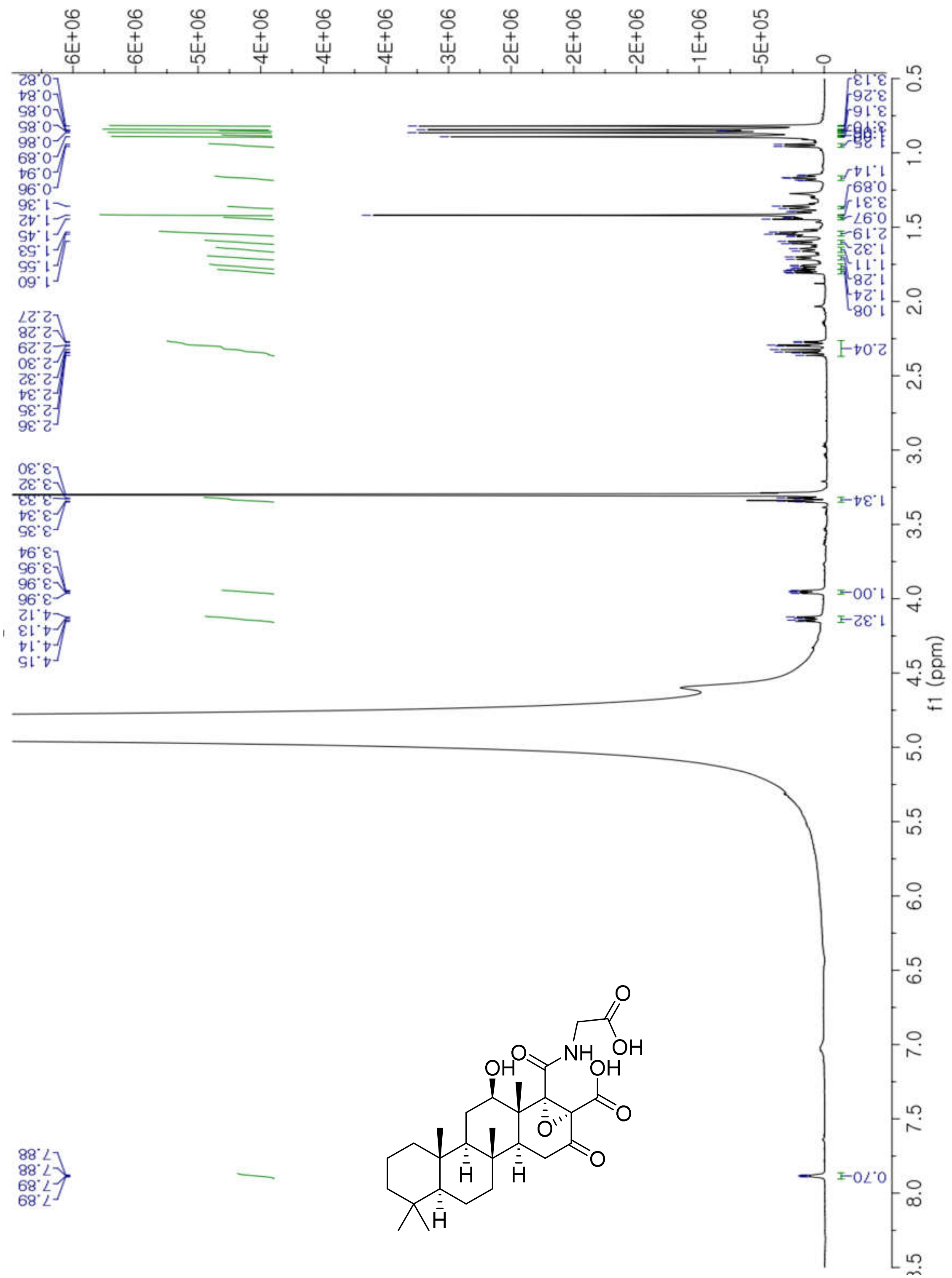

Figure S3. The ^{1}H NMR (600 MHz, MeOH-d_3) spectrum of **1**

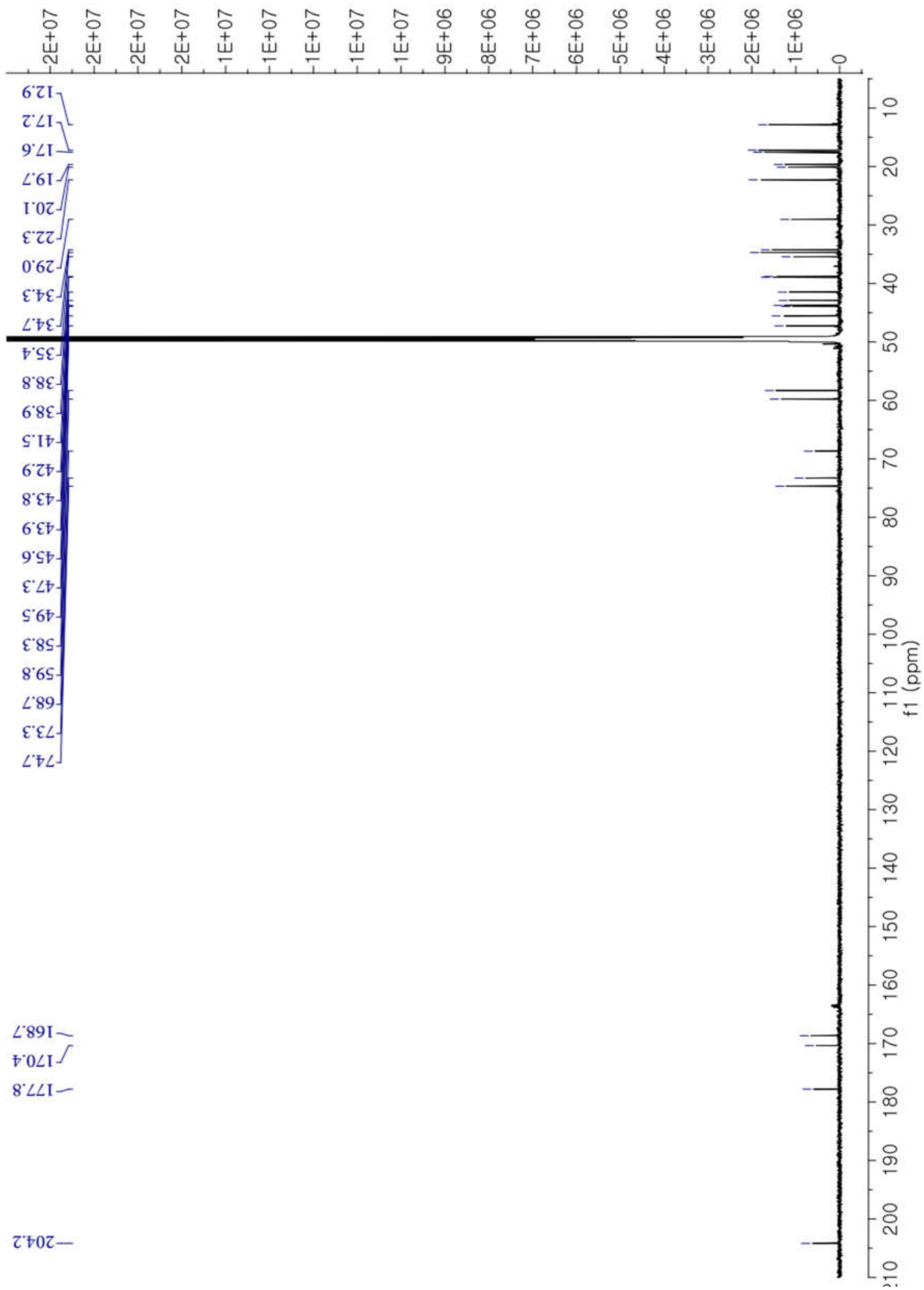

Figure S4. The ^{13}C NMR (150 MHz, MeOH-d_3) spectrum of **1**

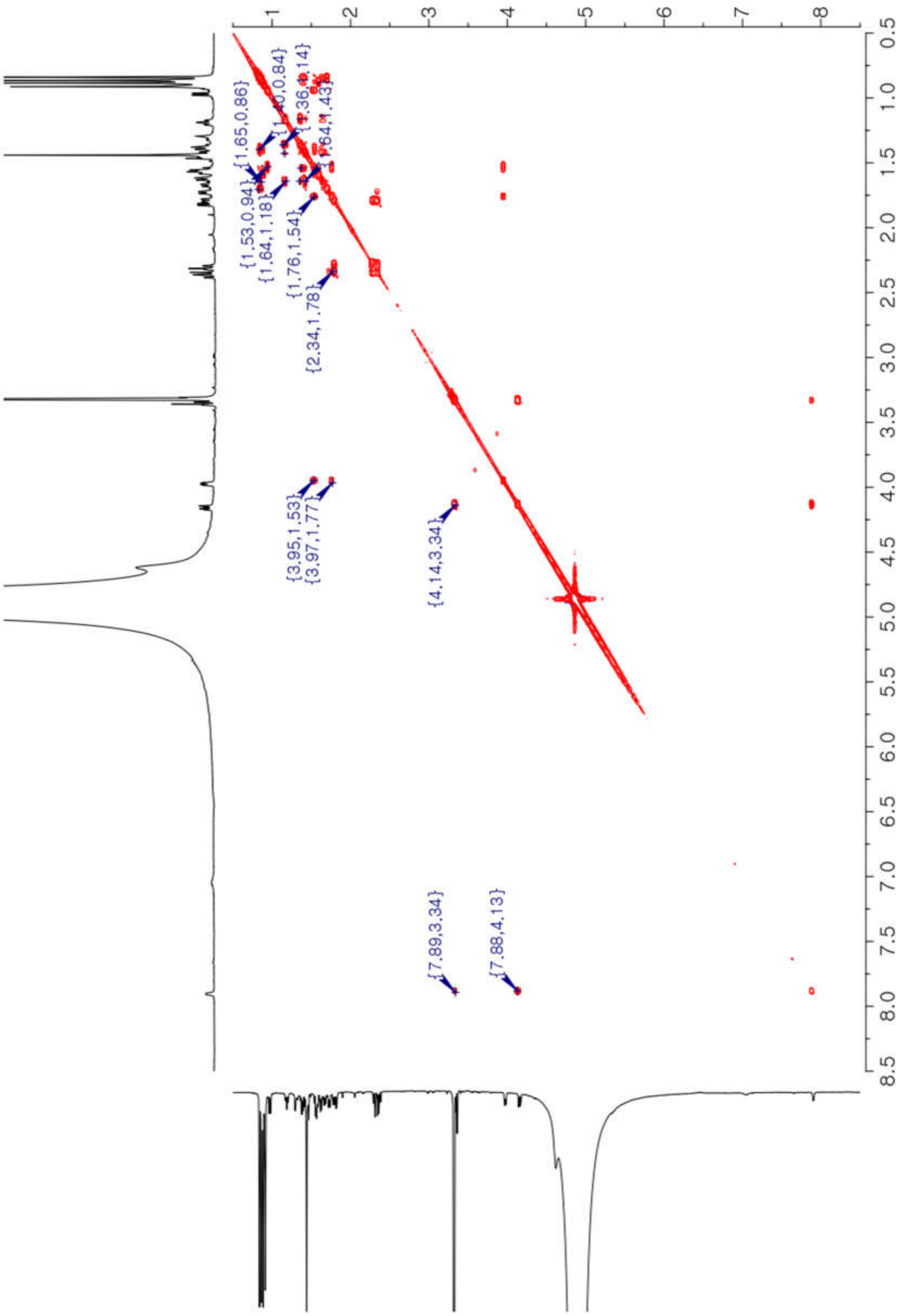

Figure S5. The COSY (600 MHz, MeOH-d_3) spectrum of **1**

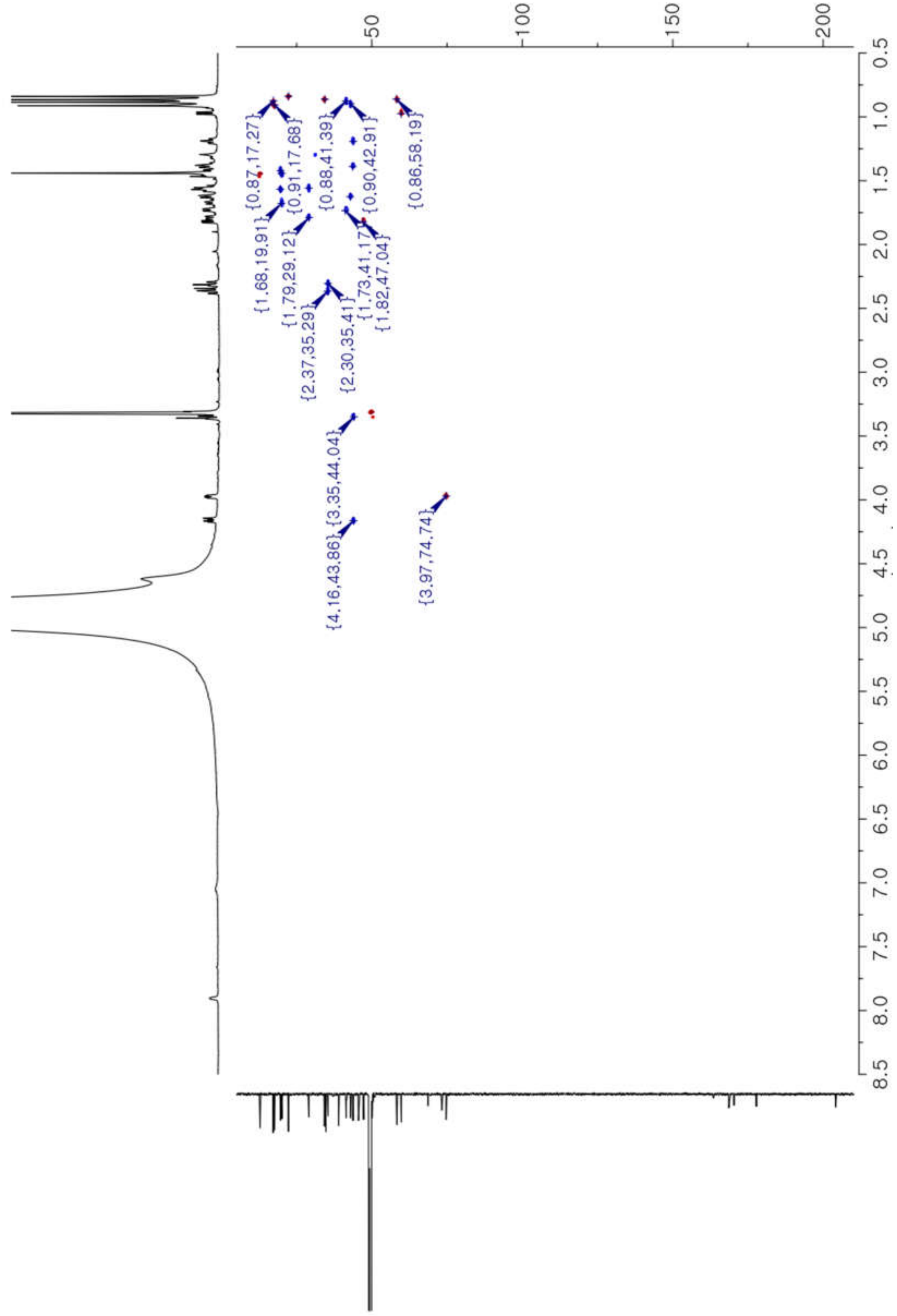

Figure S6. The eHSQC (600 MHz, MeOH-d_3) spectrum of **1**

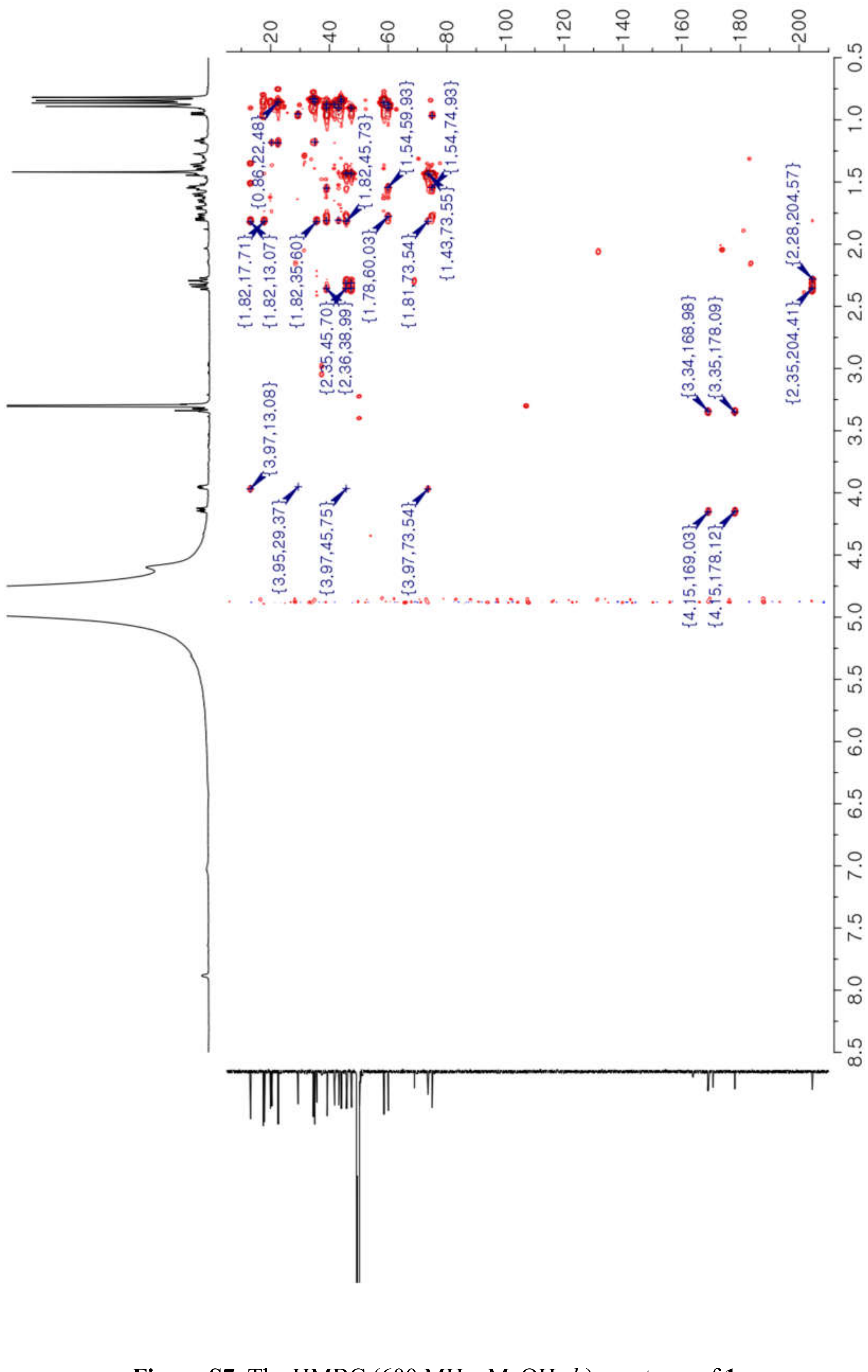

Figure S7. The HMBC (600 MHz, MeOH-d_3) spectrum of **1**

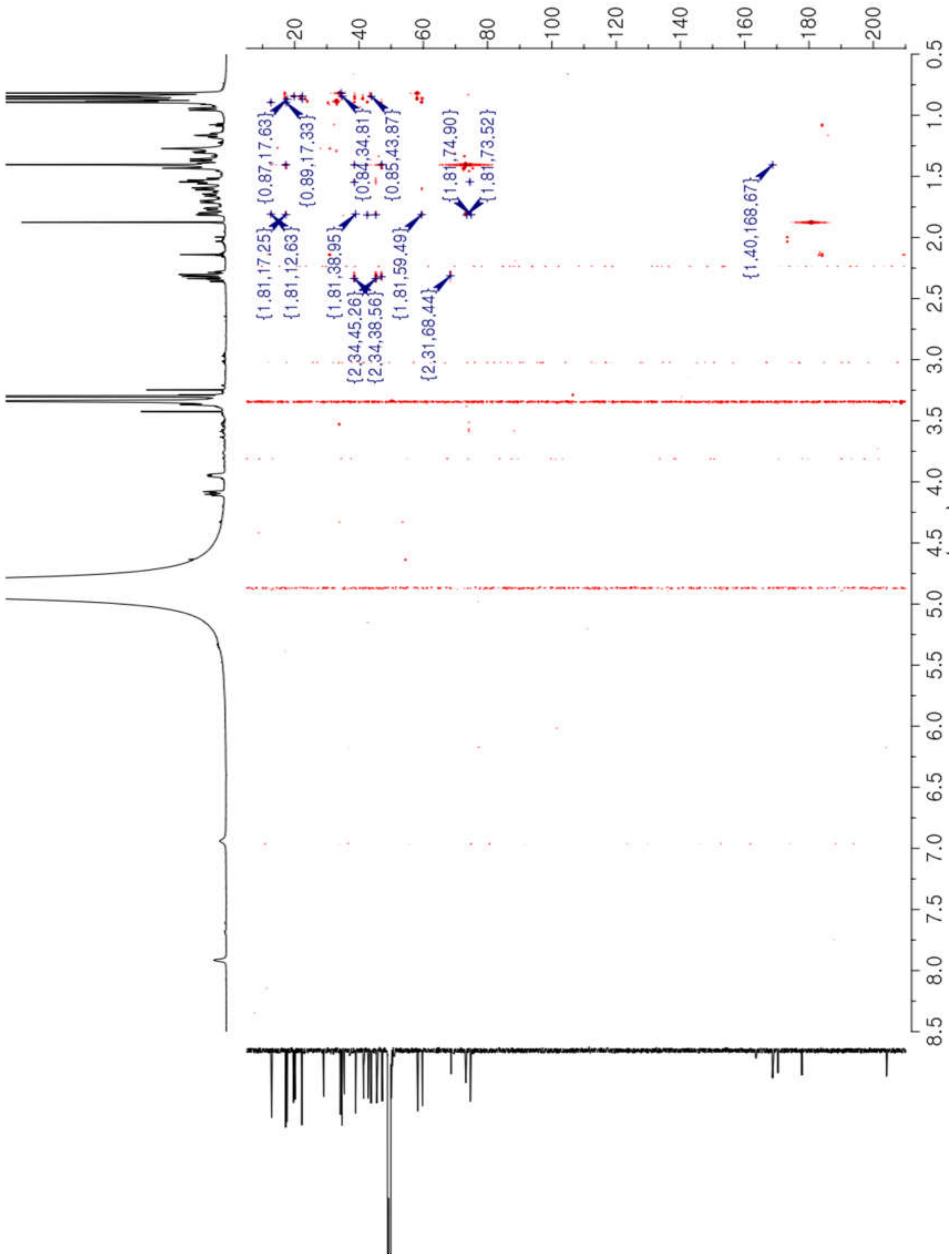

Figure S8. The LR-HSQMBC (600 MHz, MeOH-d_3) spectrum of **1**

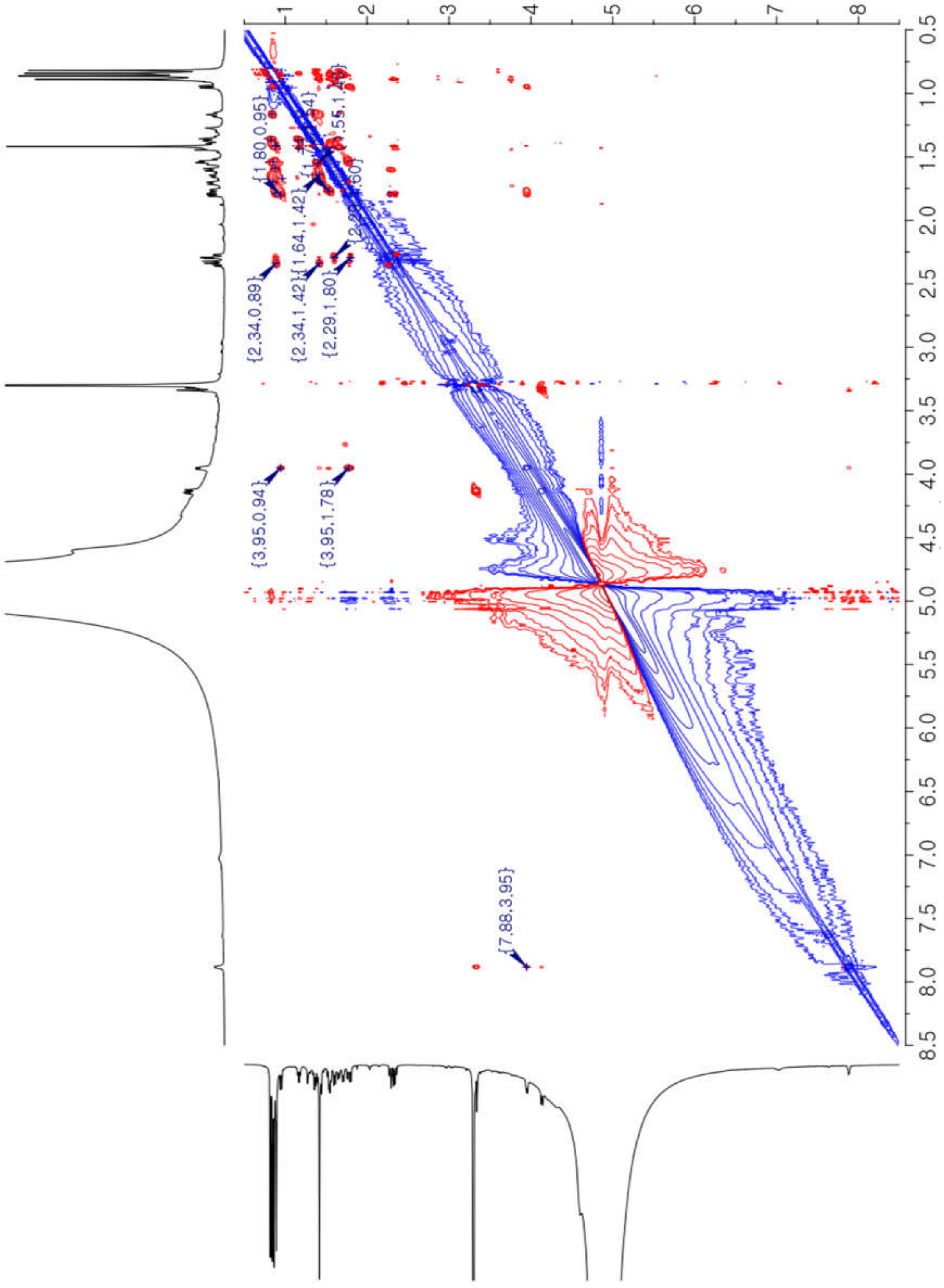

Figure S9. The NOESY (600 MHz, MeOH-d_3) spectrum of **1**

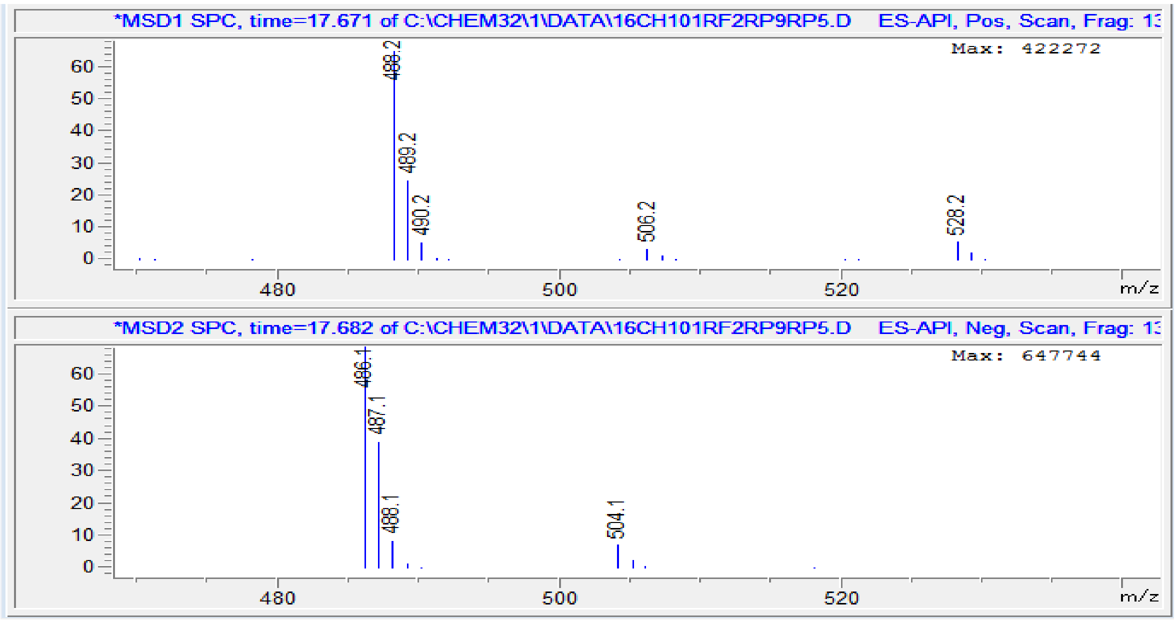

Figure S10. ESI/MS isotopic cluster patterns of compound **1** in positive and negative ion modes

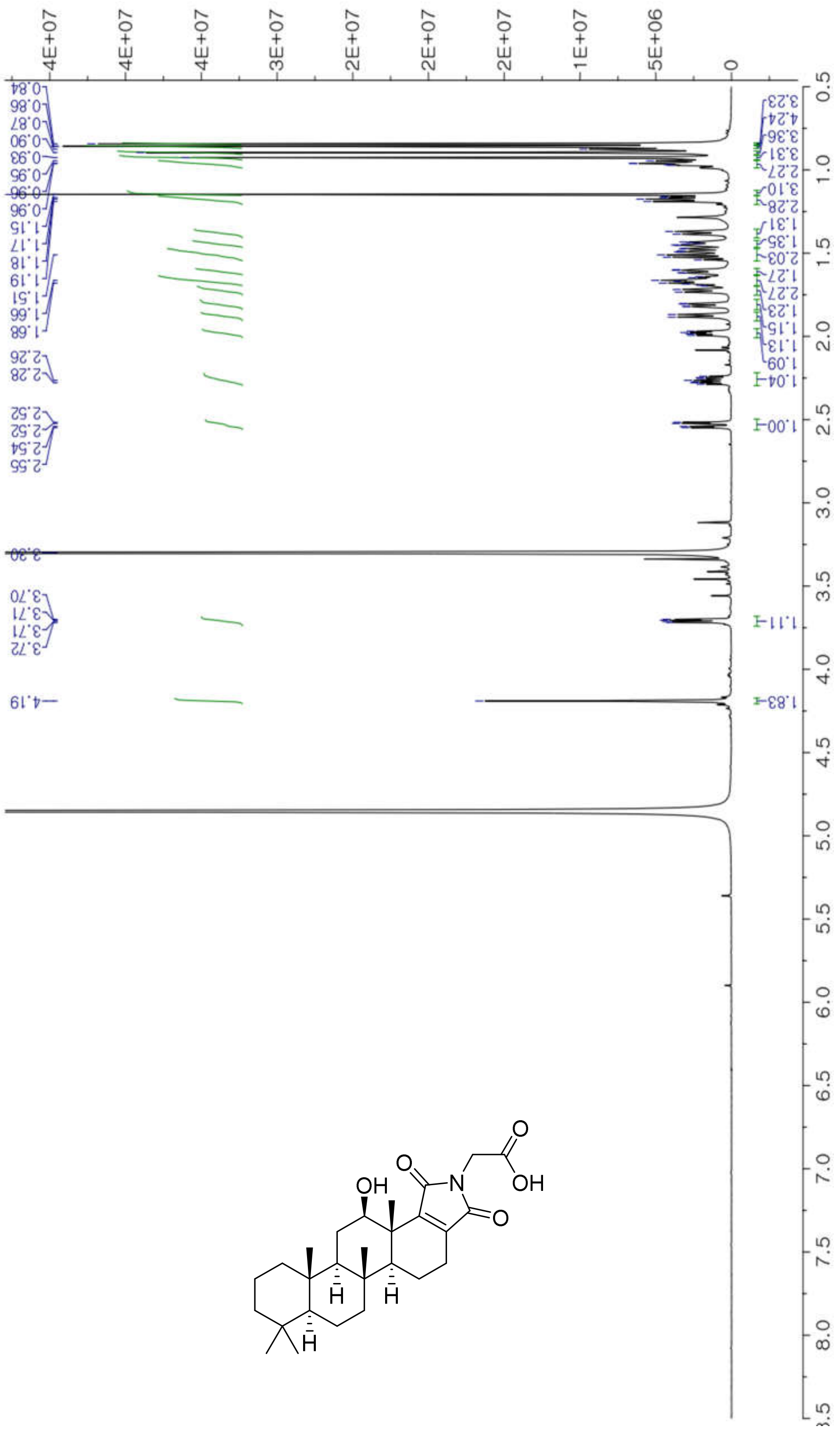

Figure S11. The ^{1}H NMR (600 MHz, MeOH-d_4) spectrum of **2**

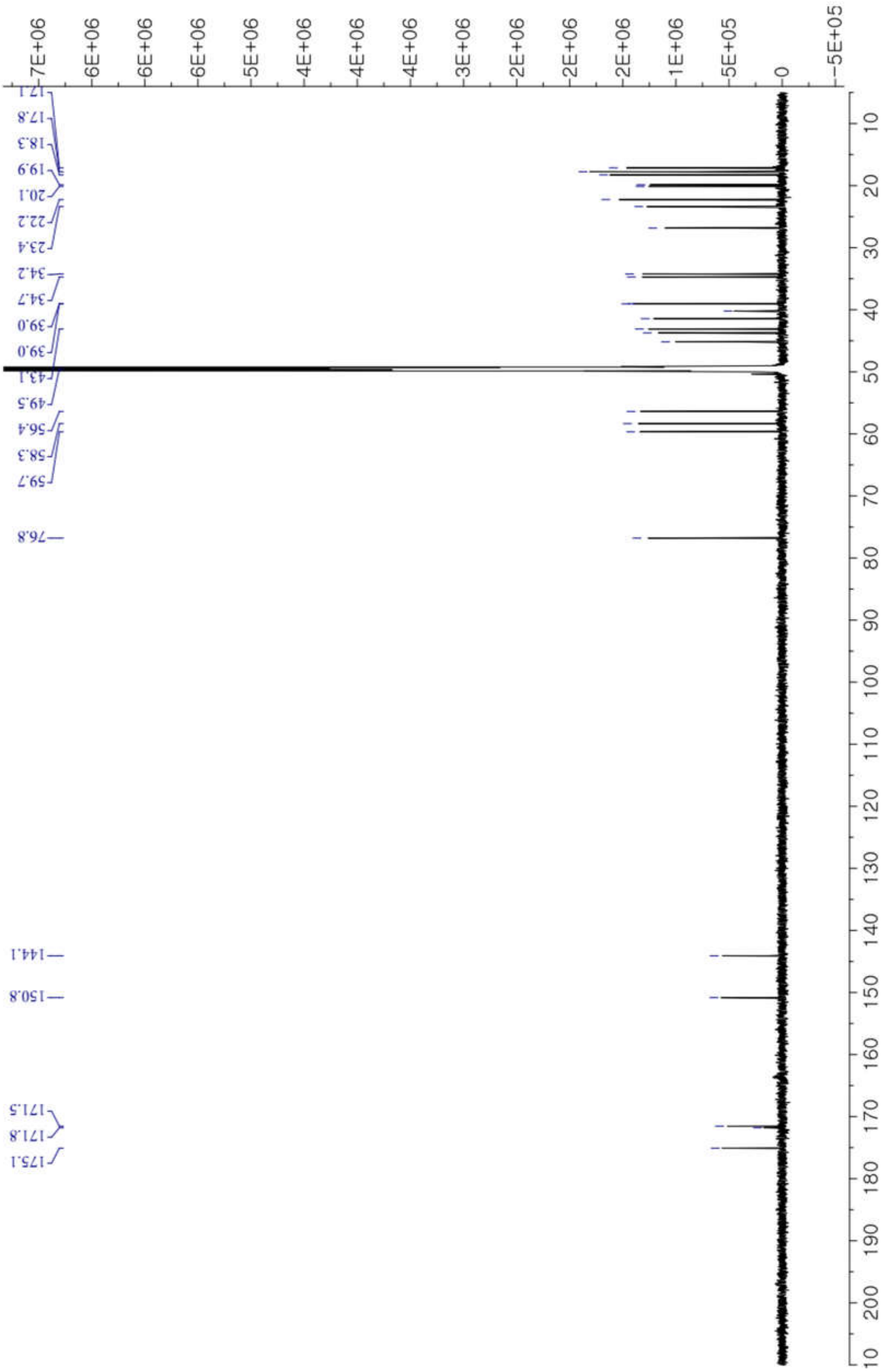

Figure S12. The ^{13}C NMR (150 MHz, MeOH-d_4) spectrum of **2**

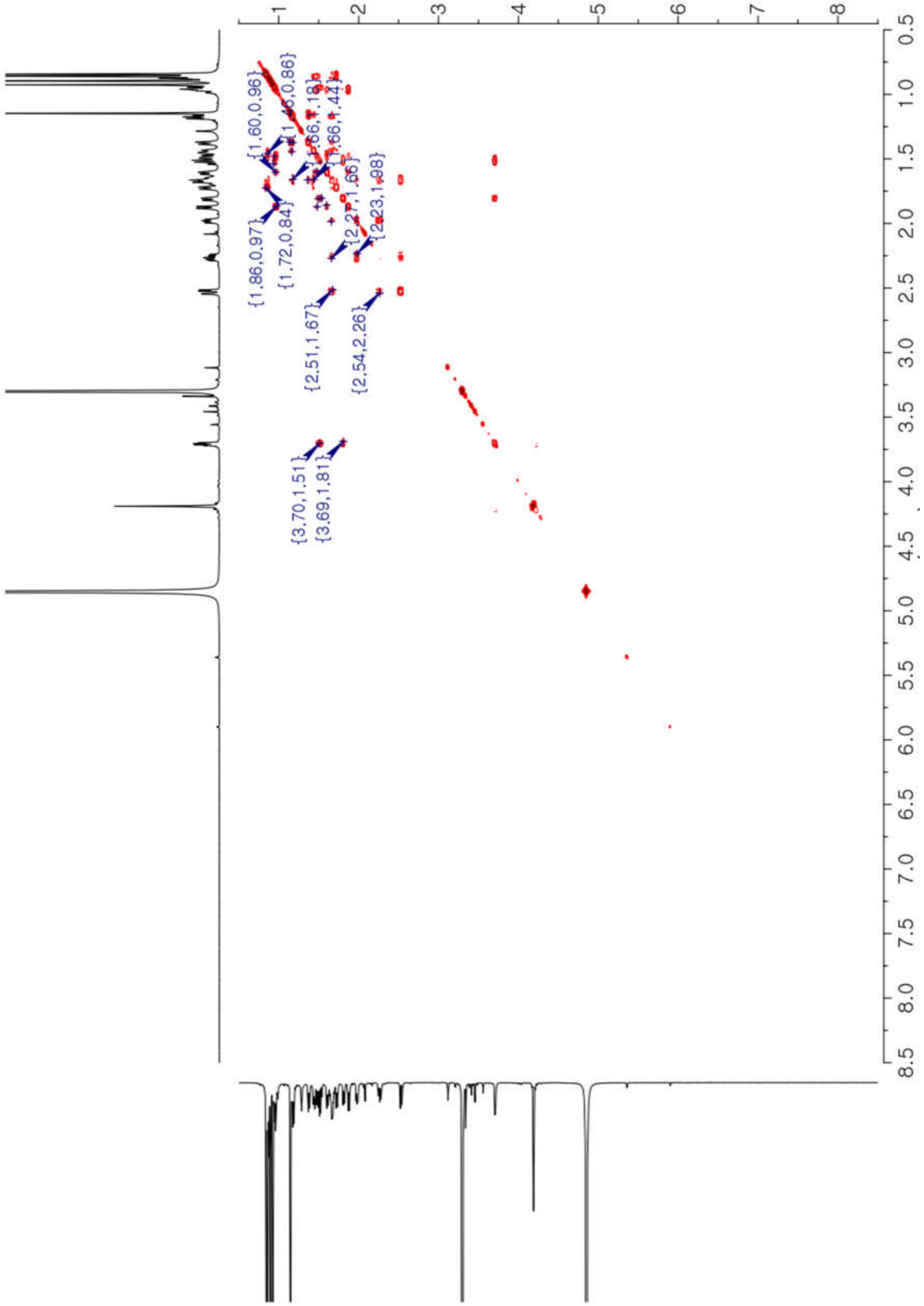

Figure S13. The COSY (600 MHz, MeOH-d_4) spectrum of **2**

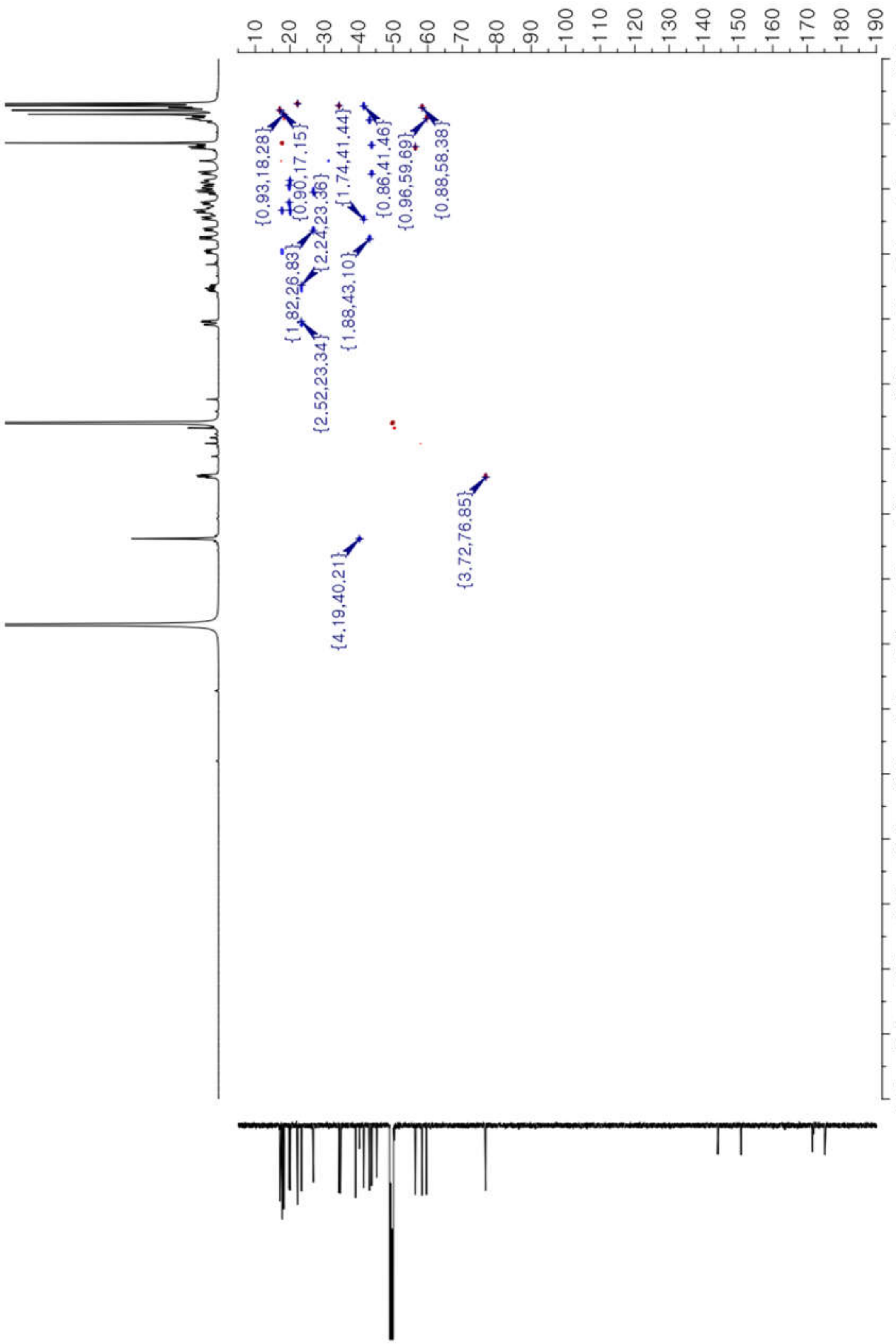

Figure S14. The eHSQC (600 MHz, MeOH-d_4) spectrum of **2**

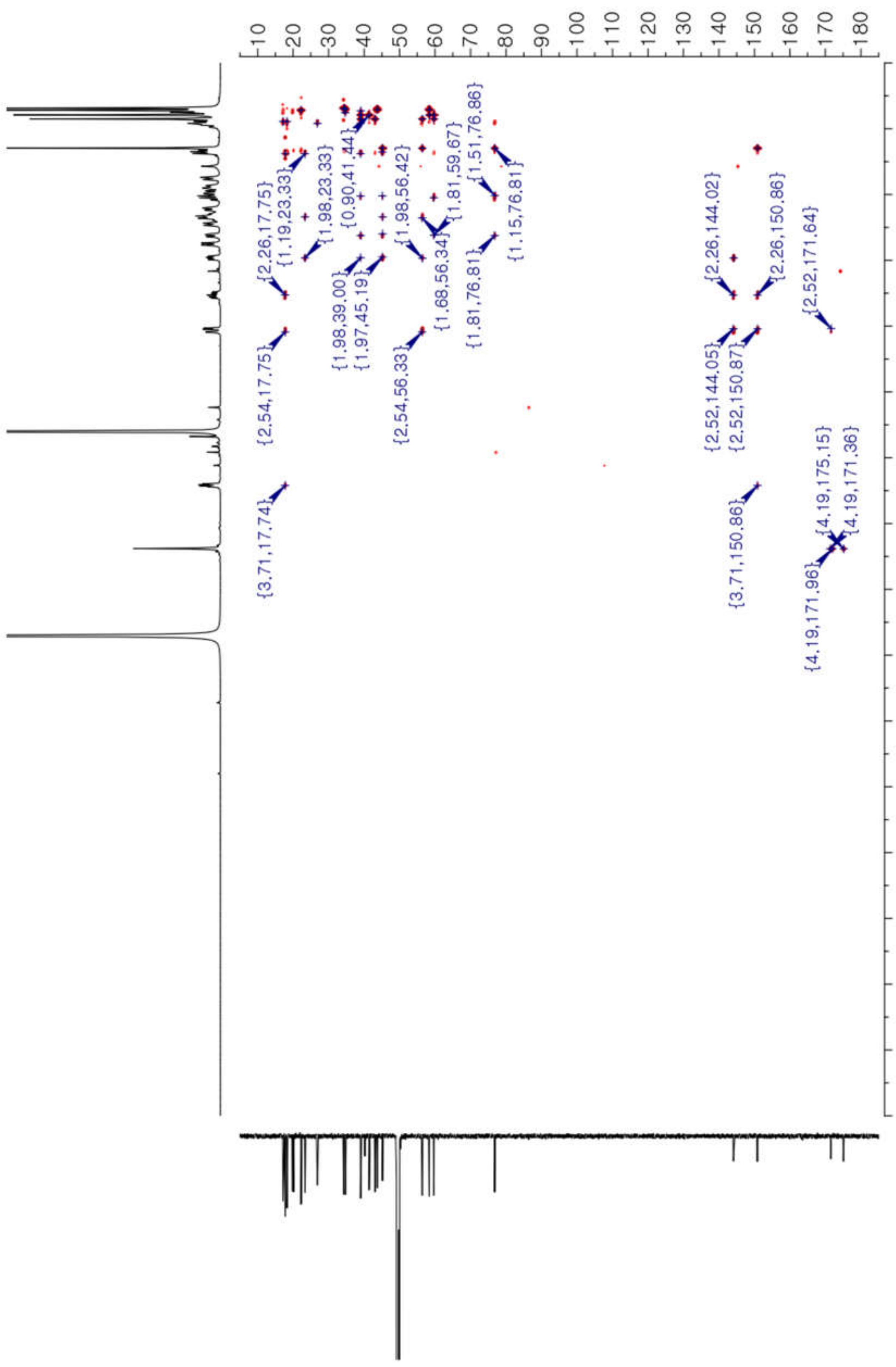

Figure S15. The HMBC (600 MHz, MeOH-d_4) spectrum of **2**

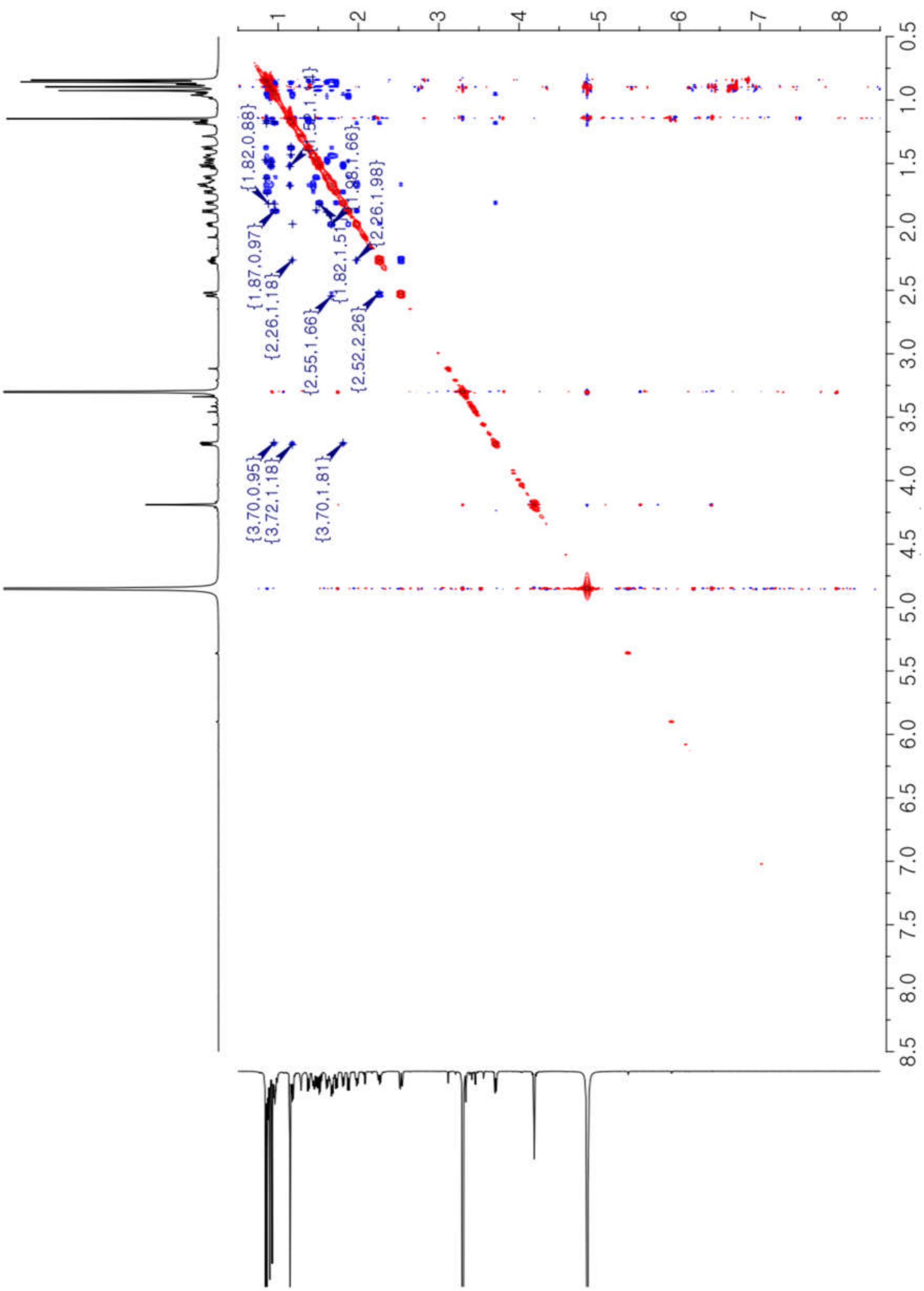

Figure S16. The NOESY (600 MHz, MeOH-d_4) spectrum of **2**

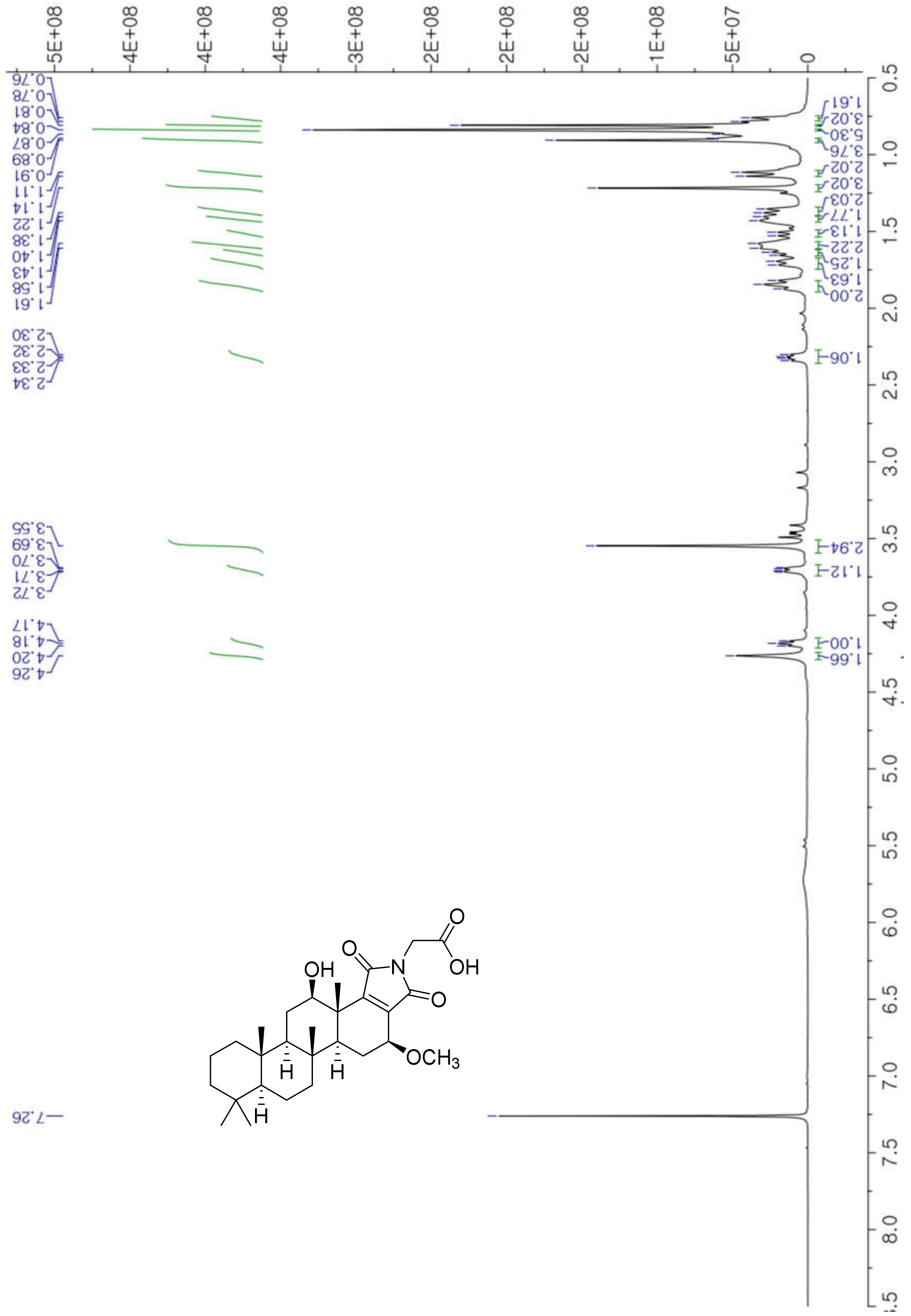

Figure S17. The ^{1}H NMR (600 MHz, $CDCl_3$) spectrum of **3**

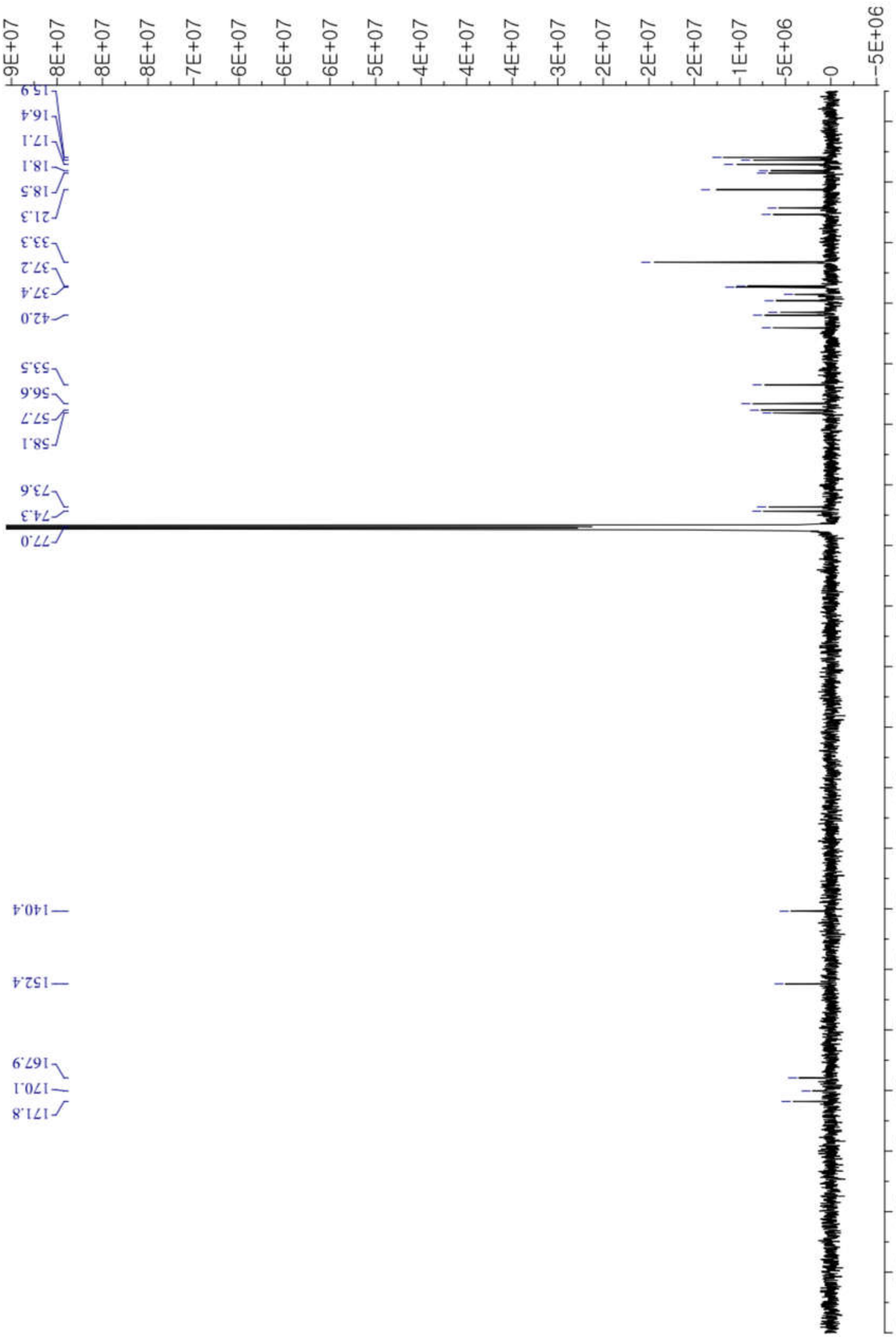

Figure S18. The ^{13}C NMR (150 MHz, $CDCl_3$) spectrum of **3**

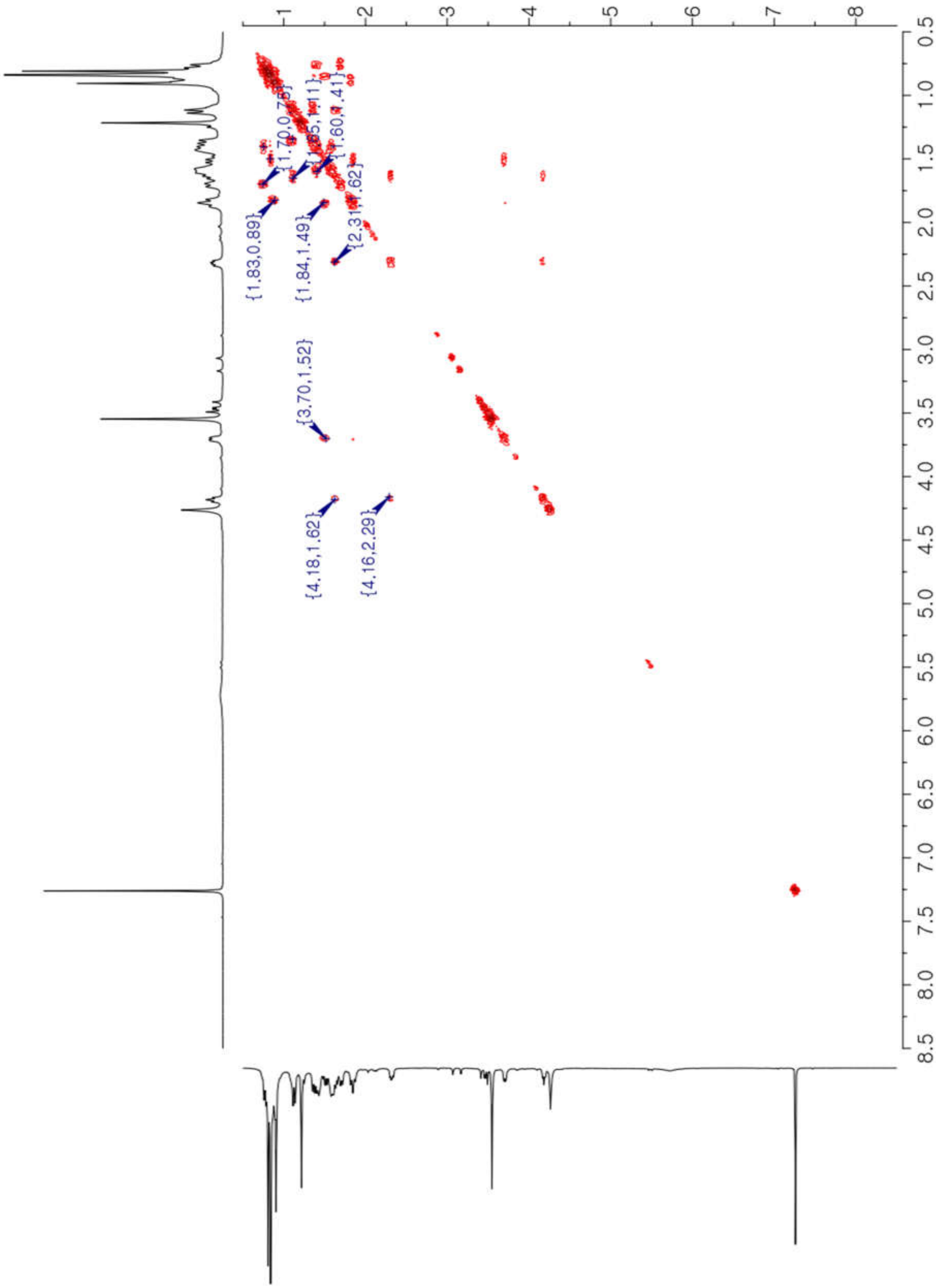

Figure S19. The COSY (600 MHz, $CDCl_3$) spectrum of **3**

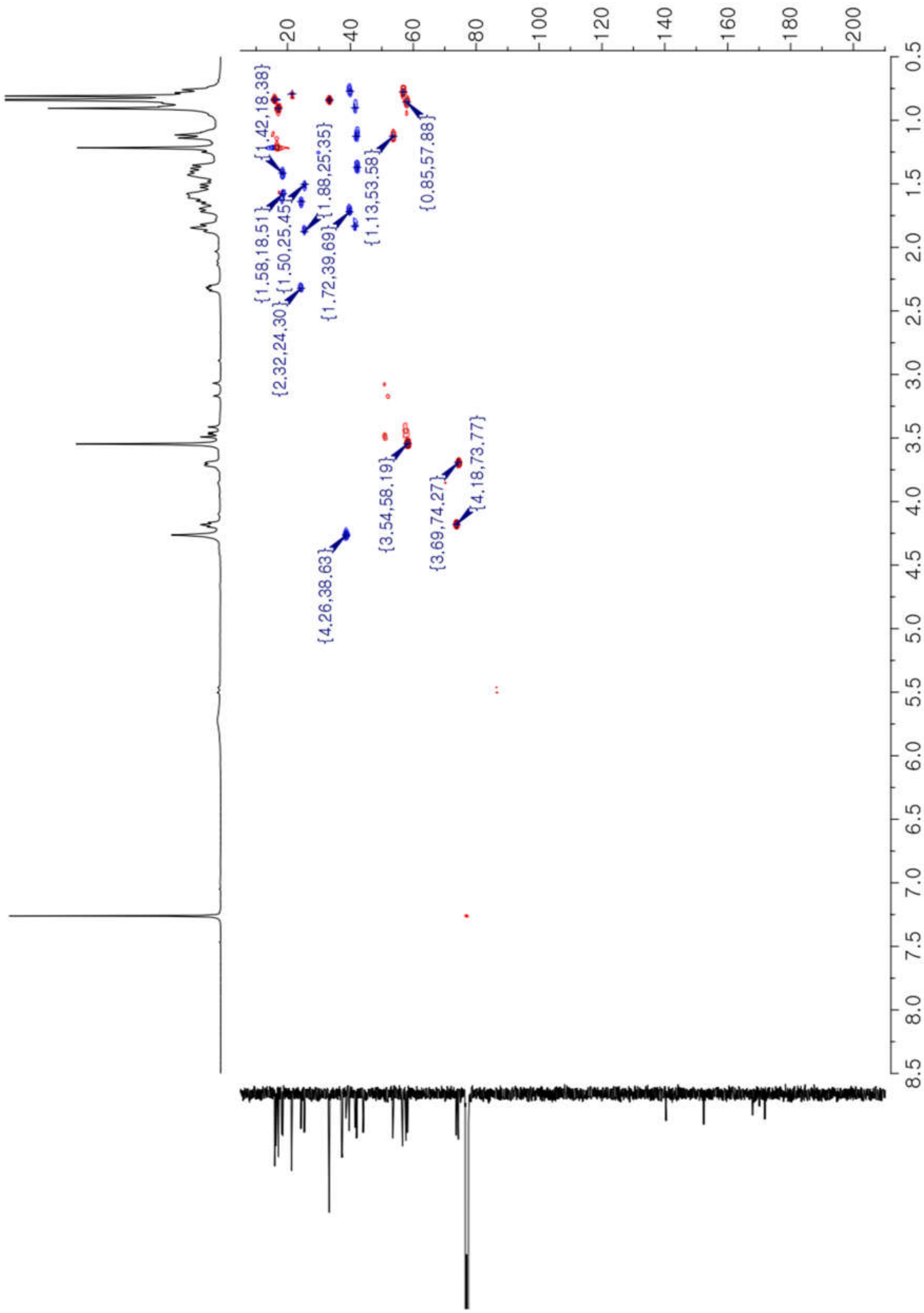

Figure S20. The eHSQC (600 MHz, $CDCl_3$) spectrum of **3**

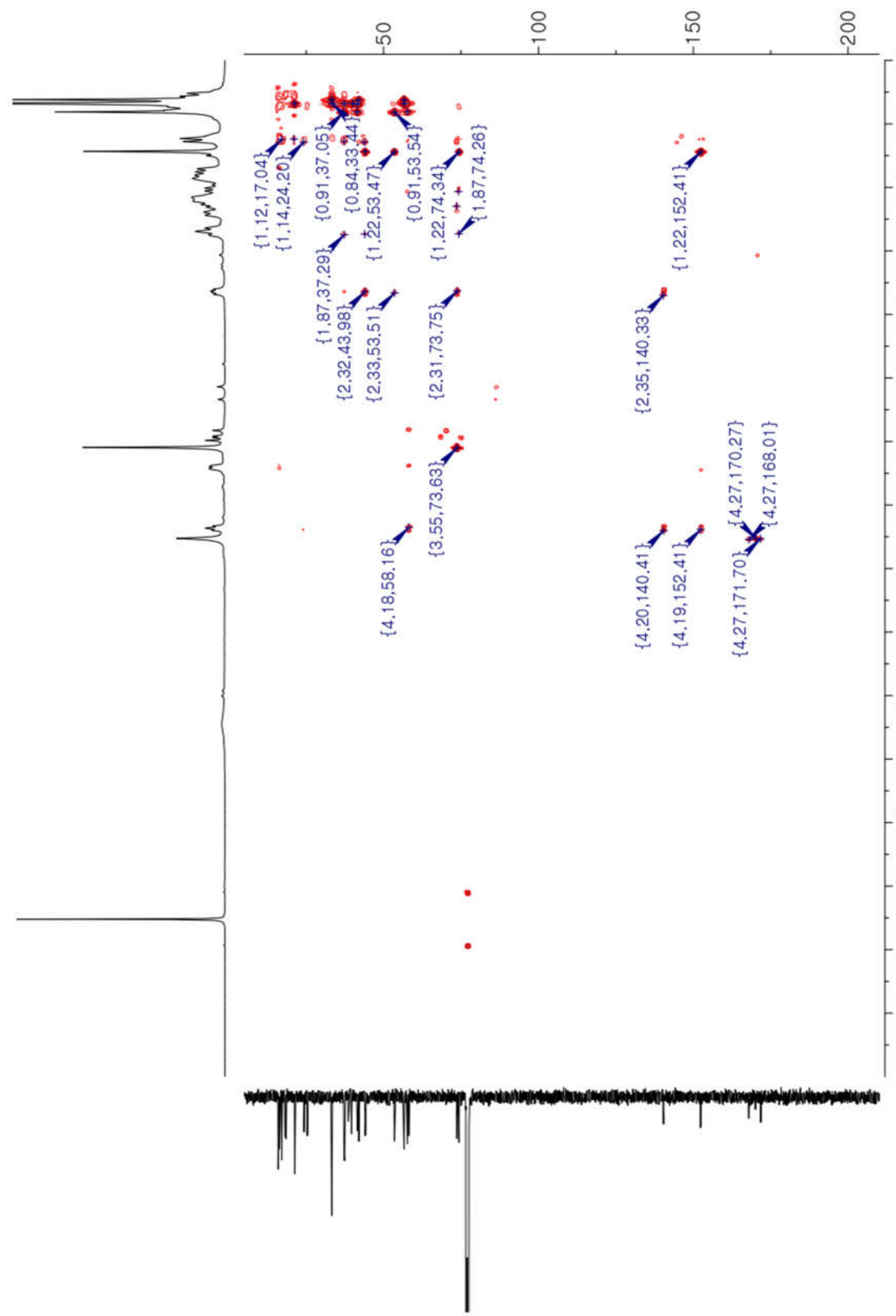

Figure S21. The HMBC (600 MHz, $CDCl_3$) spectrum of **3**

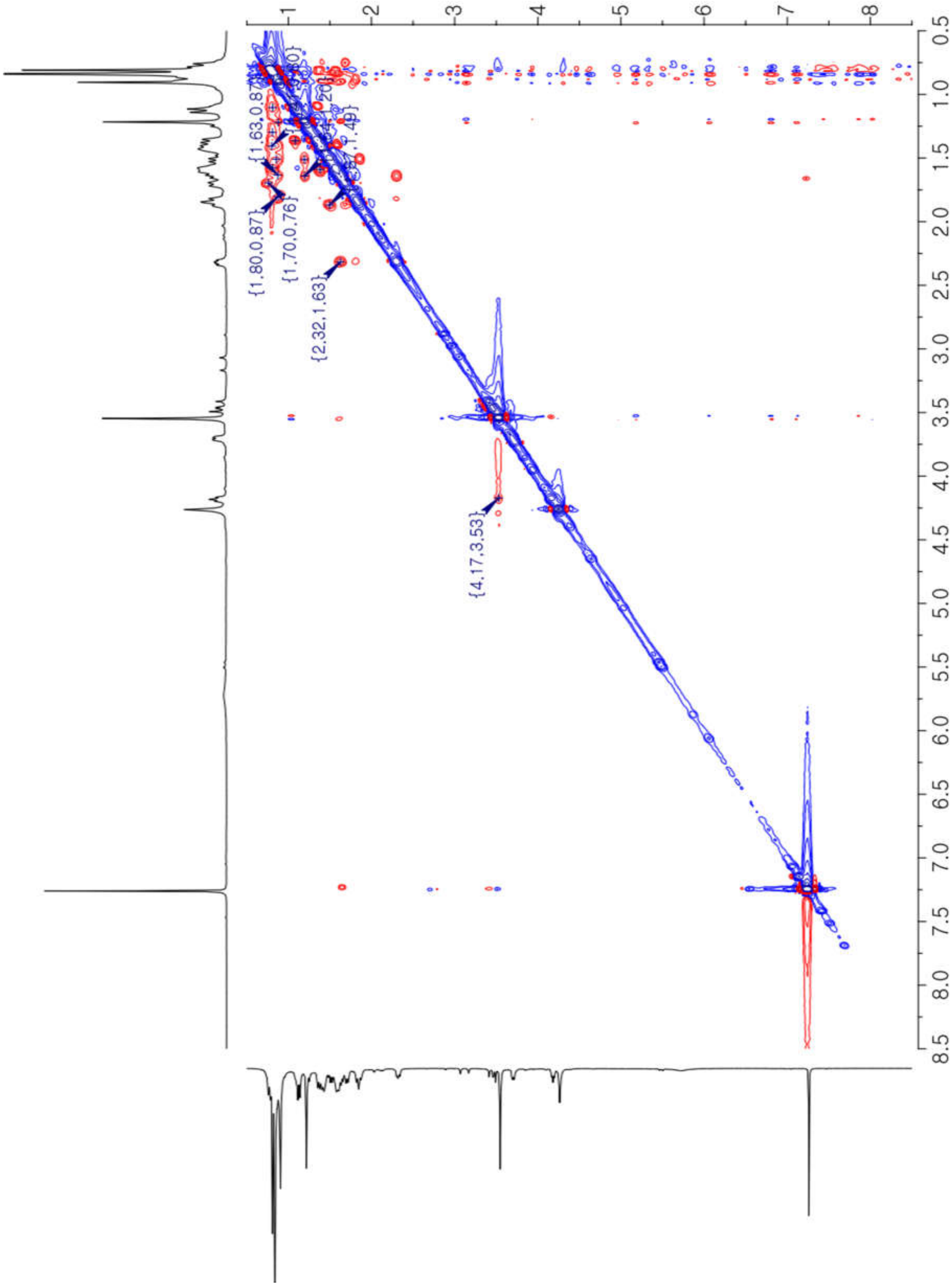

Figure S22. The NOESY (600 MHz, $CDCl_3$) spectrum of **3**

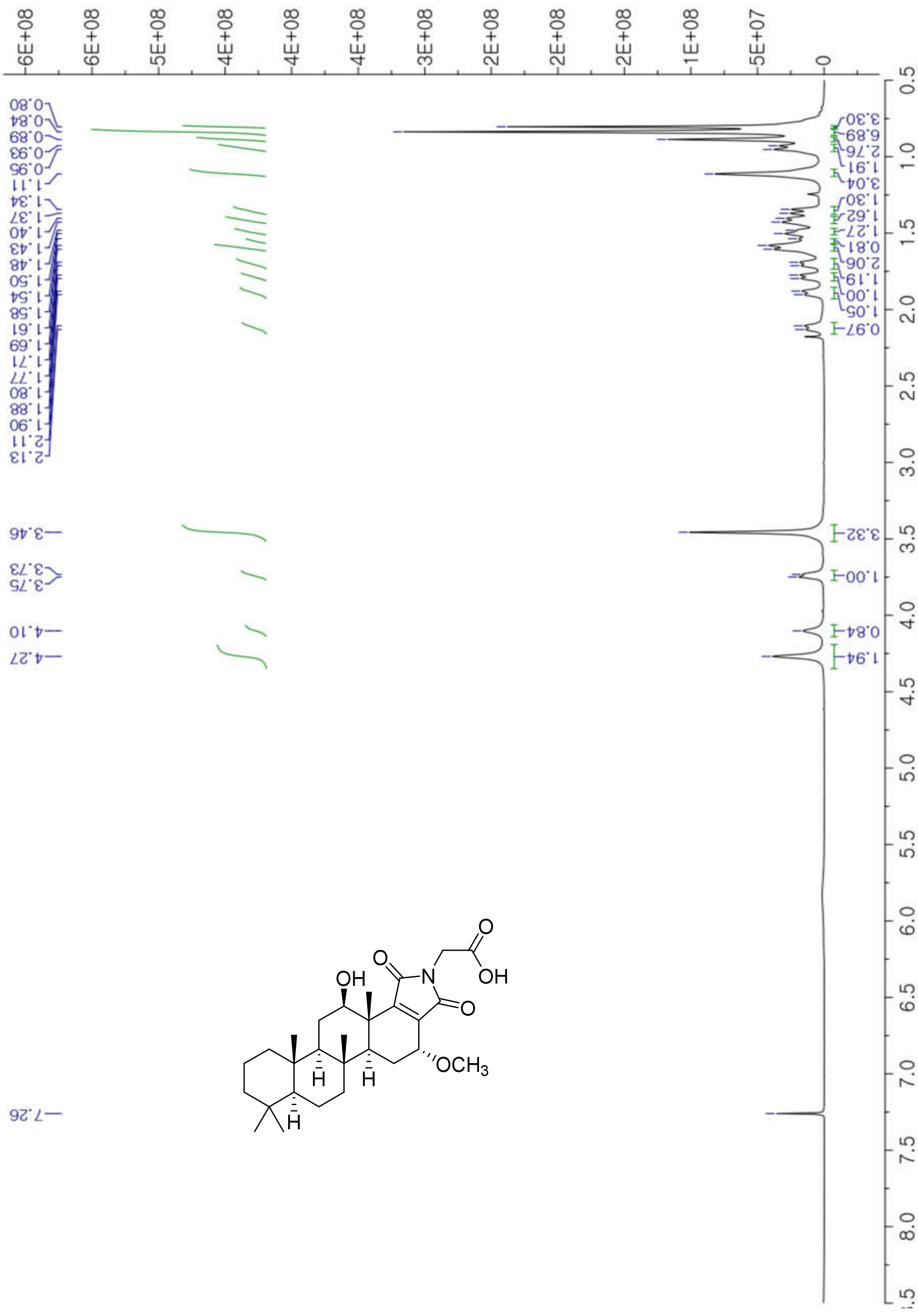

Figure S23. The ^{1}H NMR (600 MHz, $CDCl_3$) spectrum of **4**

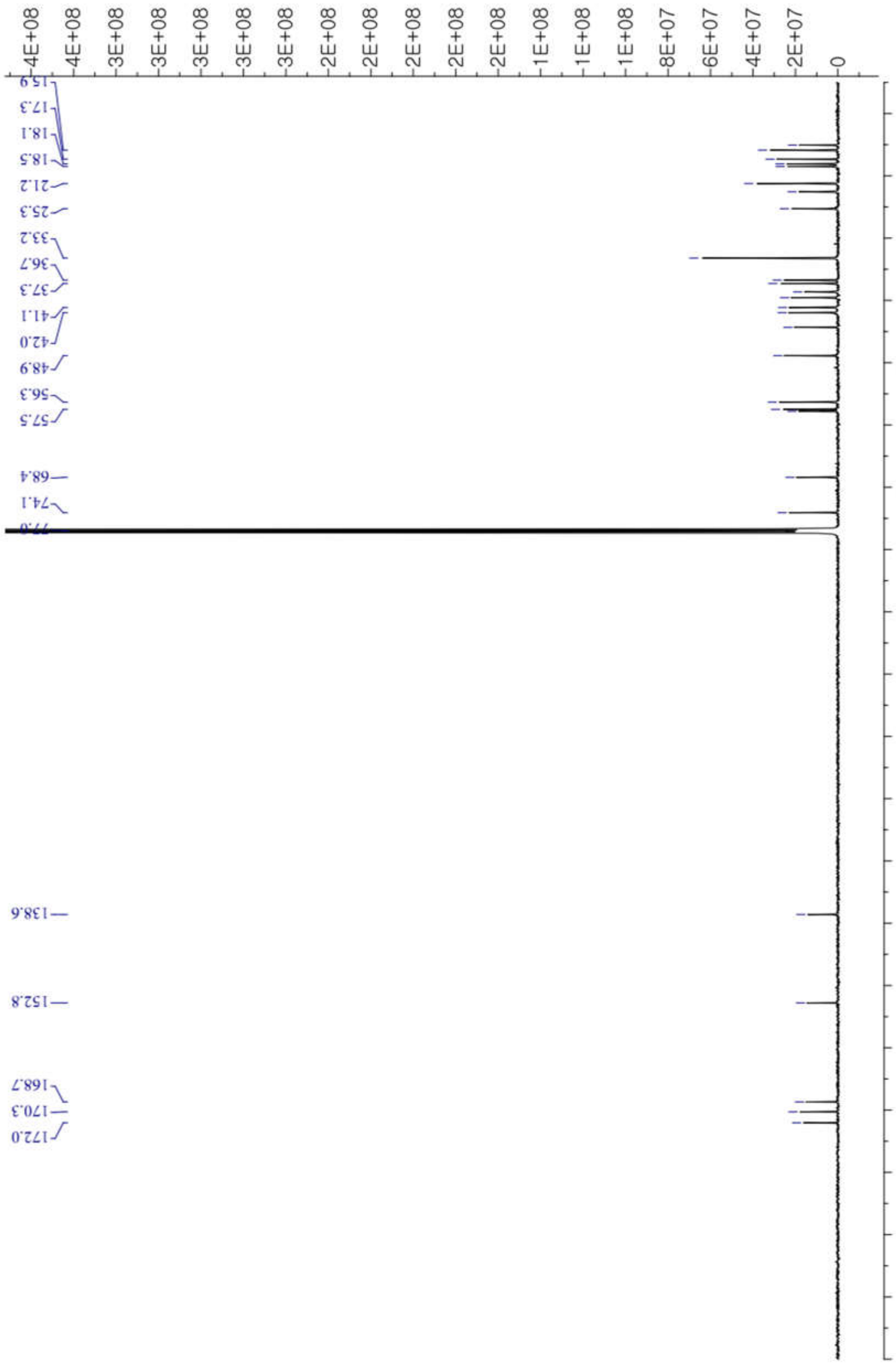

Figure S24. The ^{13}C NMR (150 MHz, $CDCl_3$) spectrum of **4**

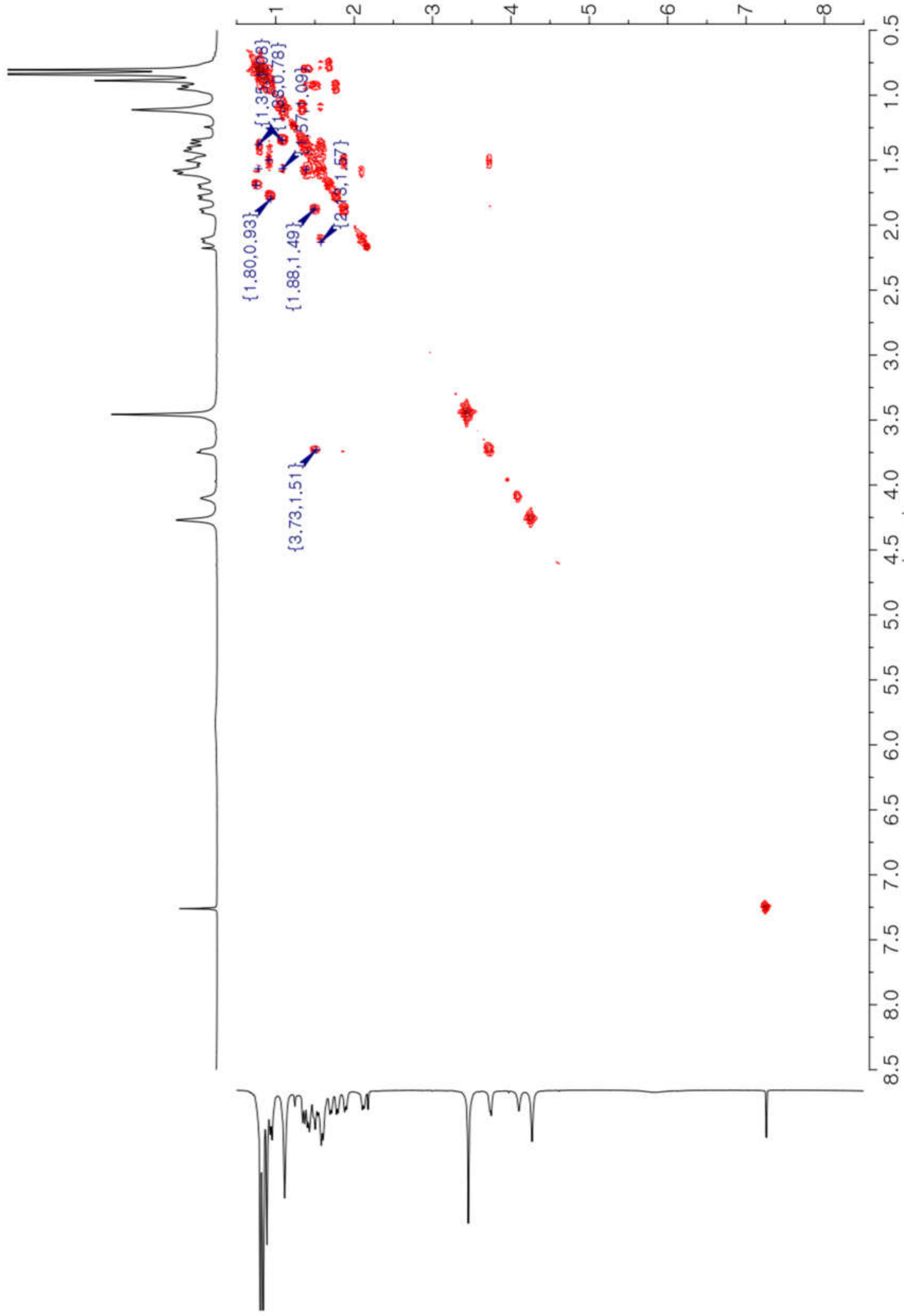

Figure S25. The COSY (600 MHz, $CDCl_3$) spectrum of **4**

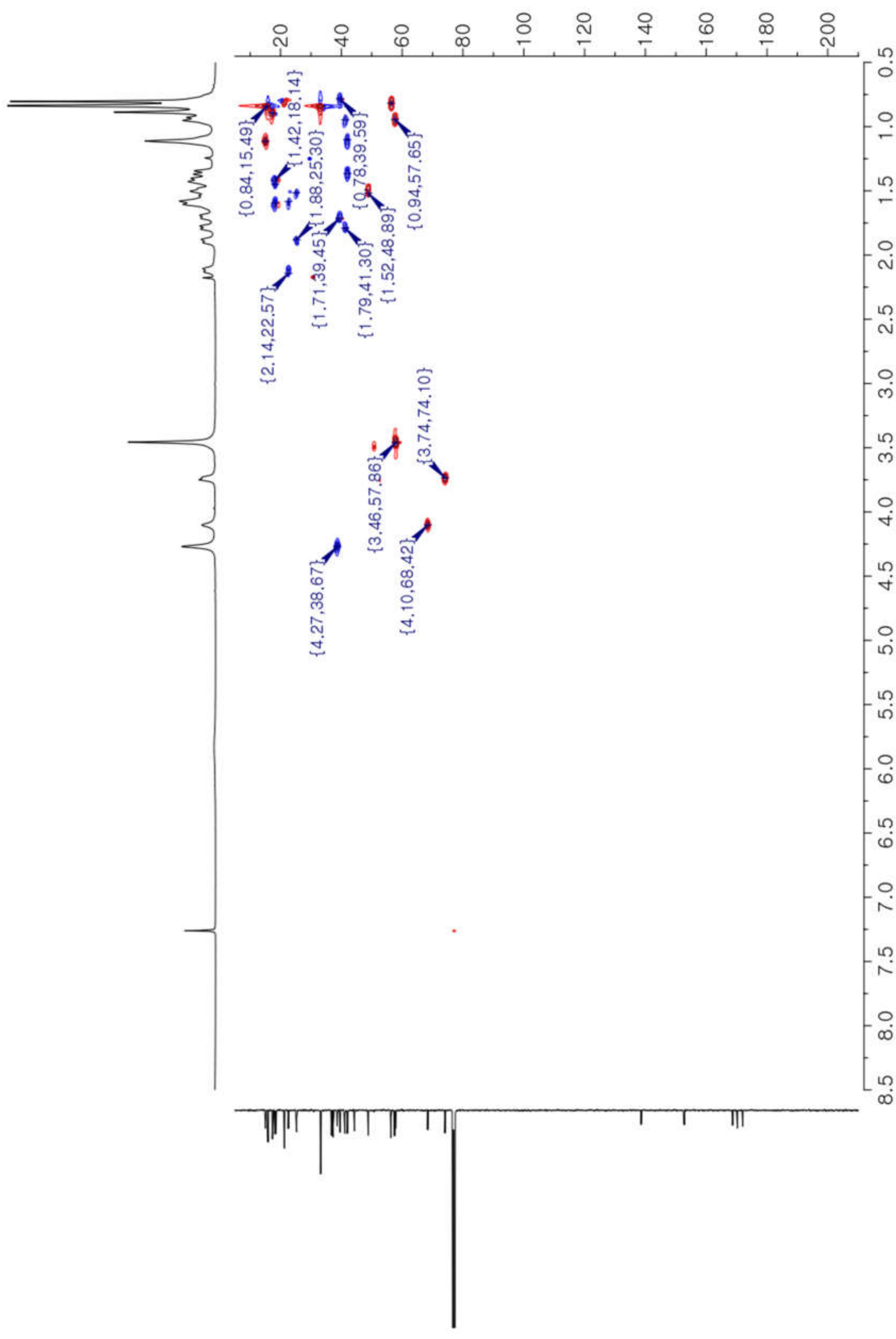

Figure S26. The eHSQC (600 MHz, $CDCl_3$) spectrum of **4**

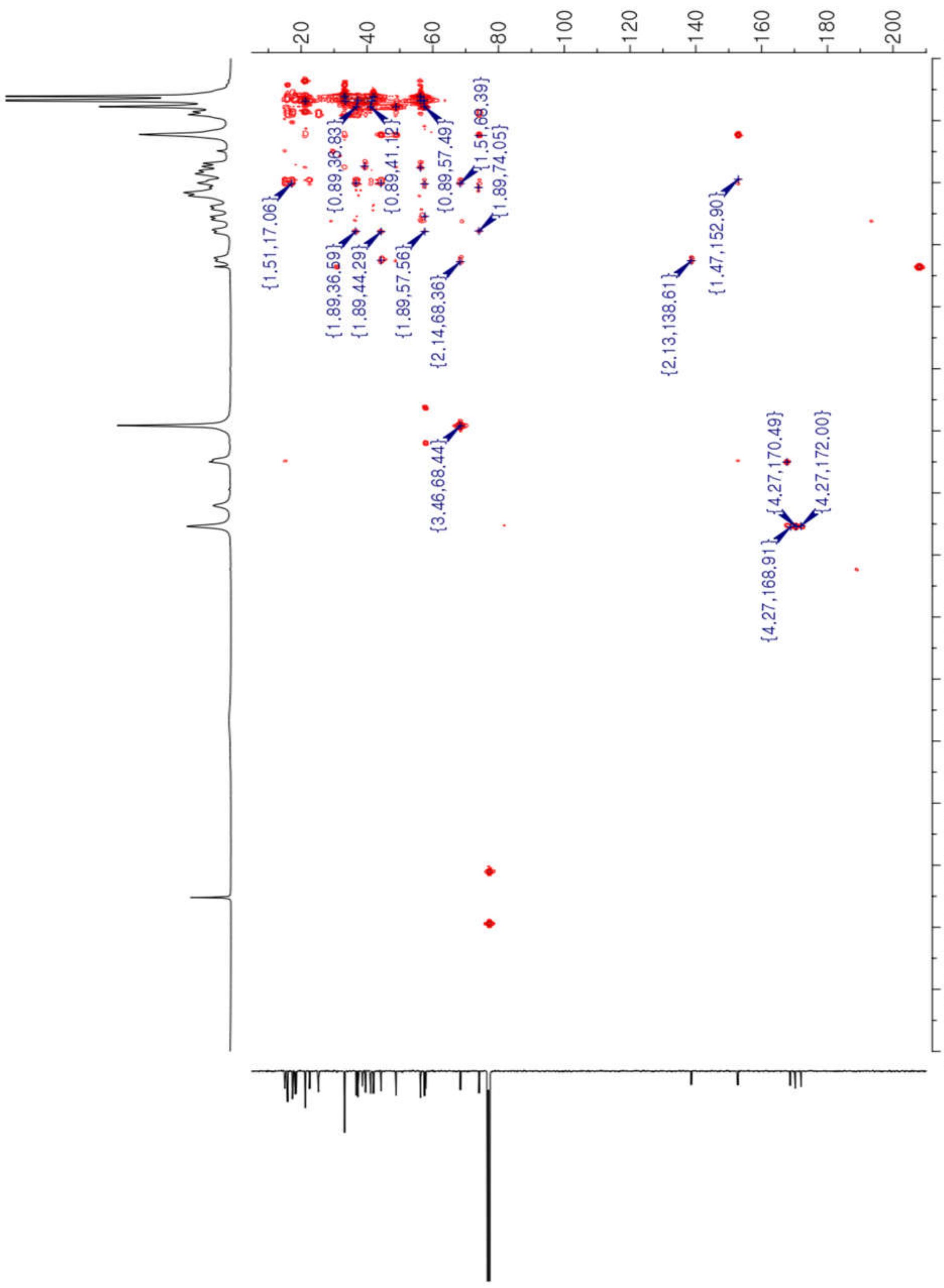

Figure S27. The HMBC (600 MHz, $CDCl_3$) spectrum of **4**

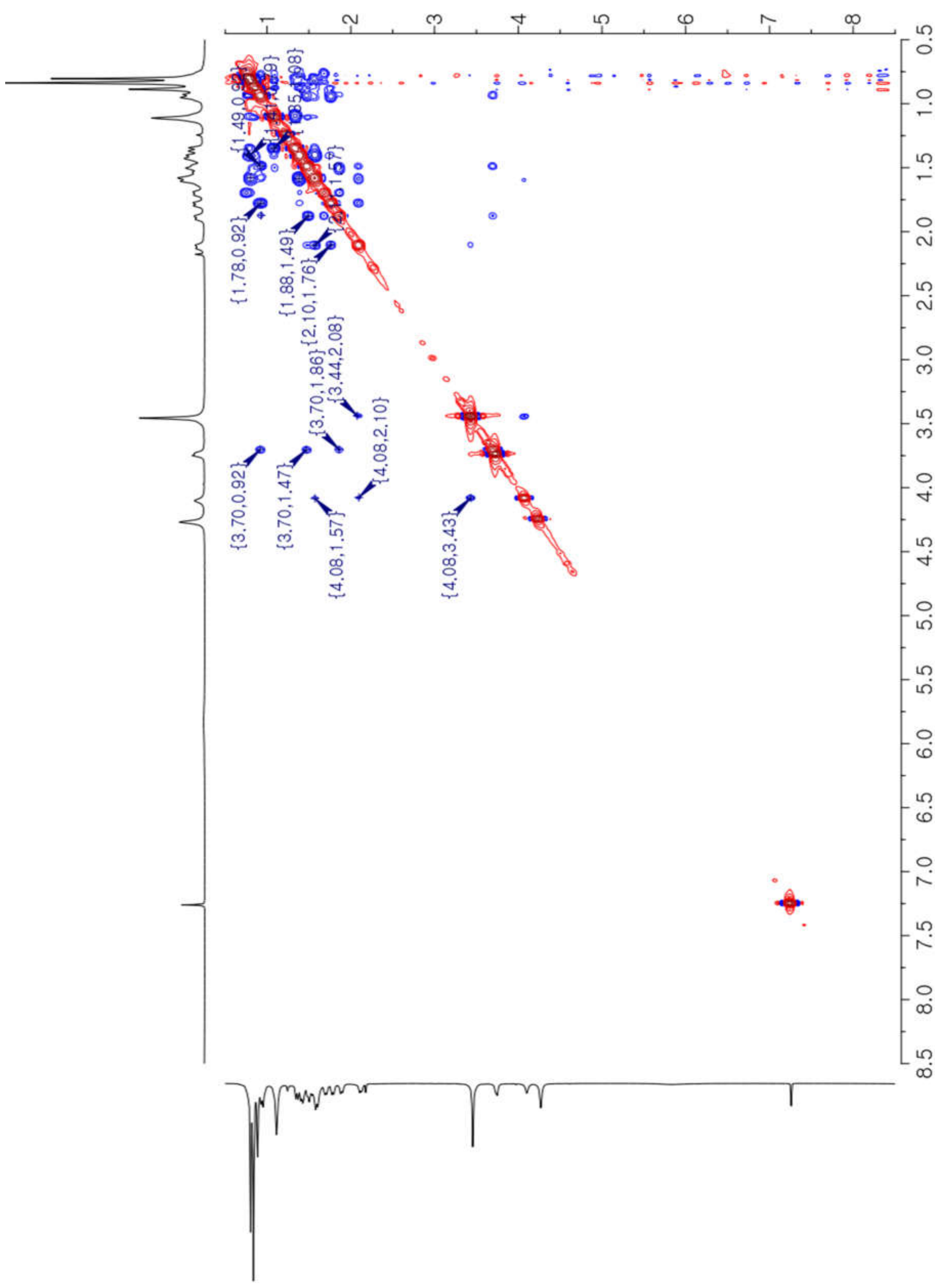

Figure S28. The NOESY (600 MHz, $CDCl_3$) spectrum of **4**

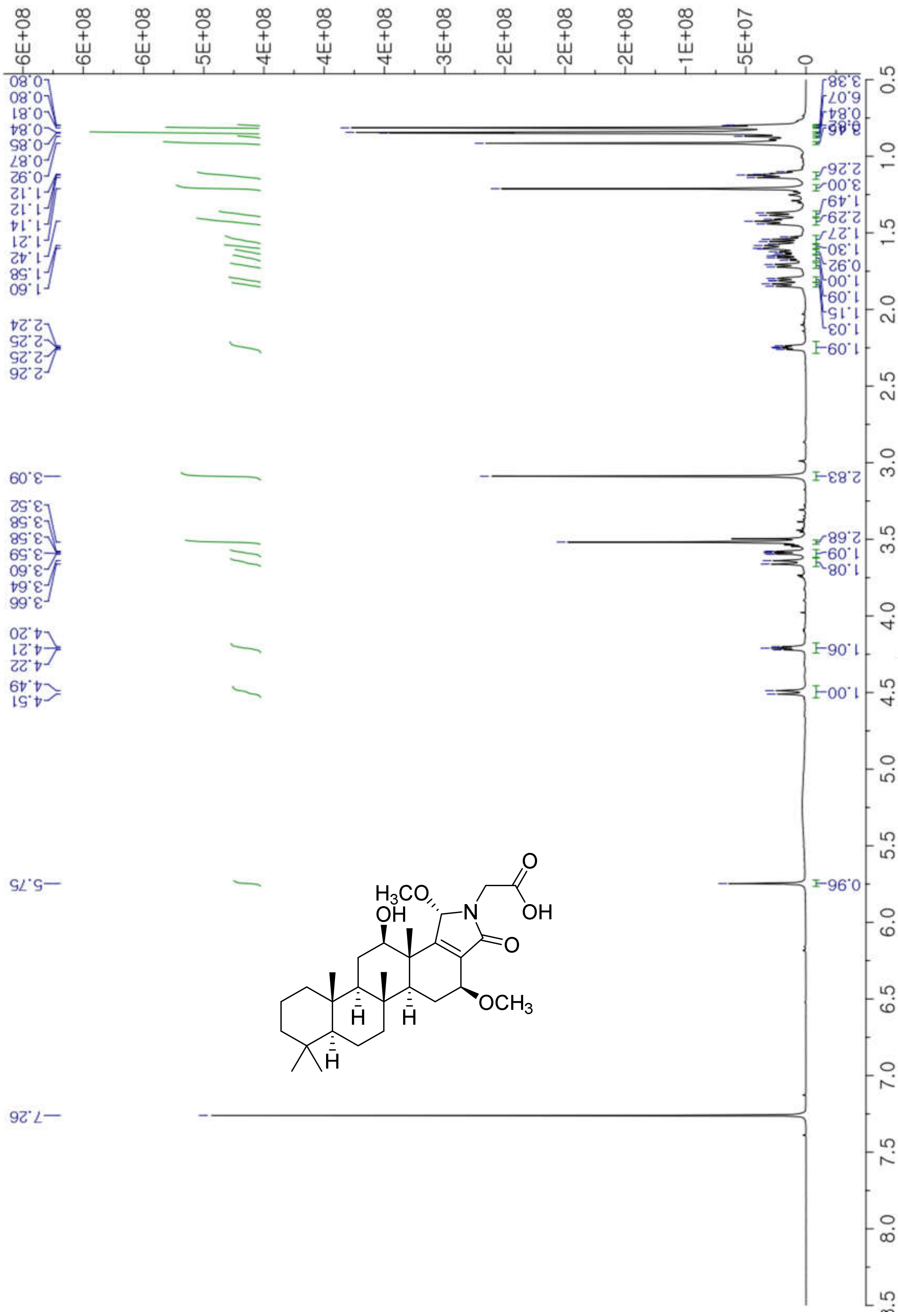

Figure S29. The ^{1}H NMR (600 MHz, $CDCl_3$) spectrum of **5**

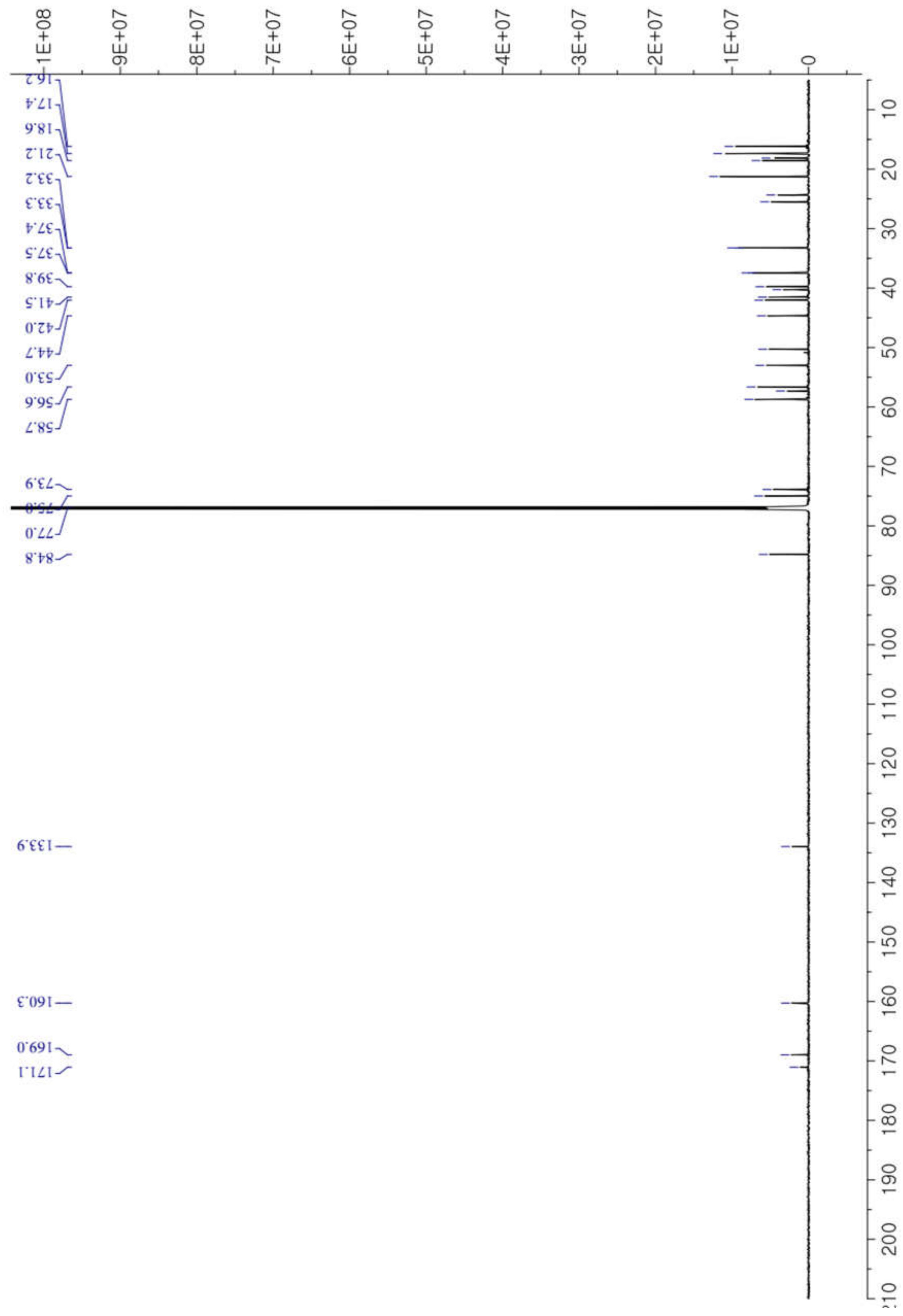

Figure S30. The ^{13}C NMR (150 MHz, $CDCl_3$) spectrum of **5**

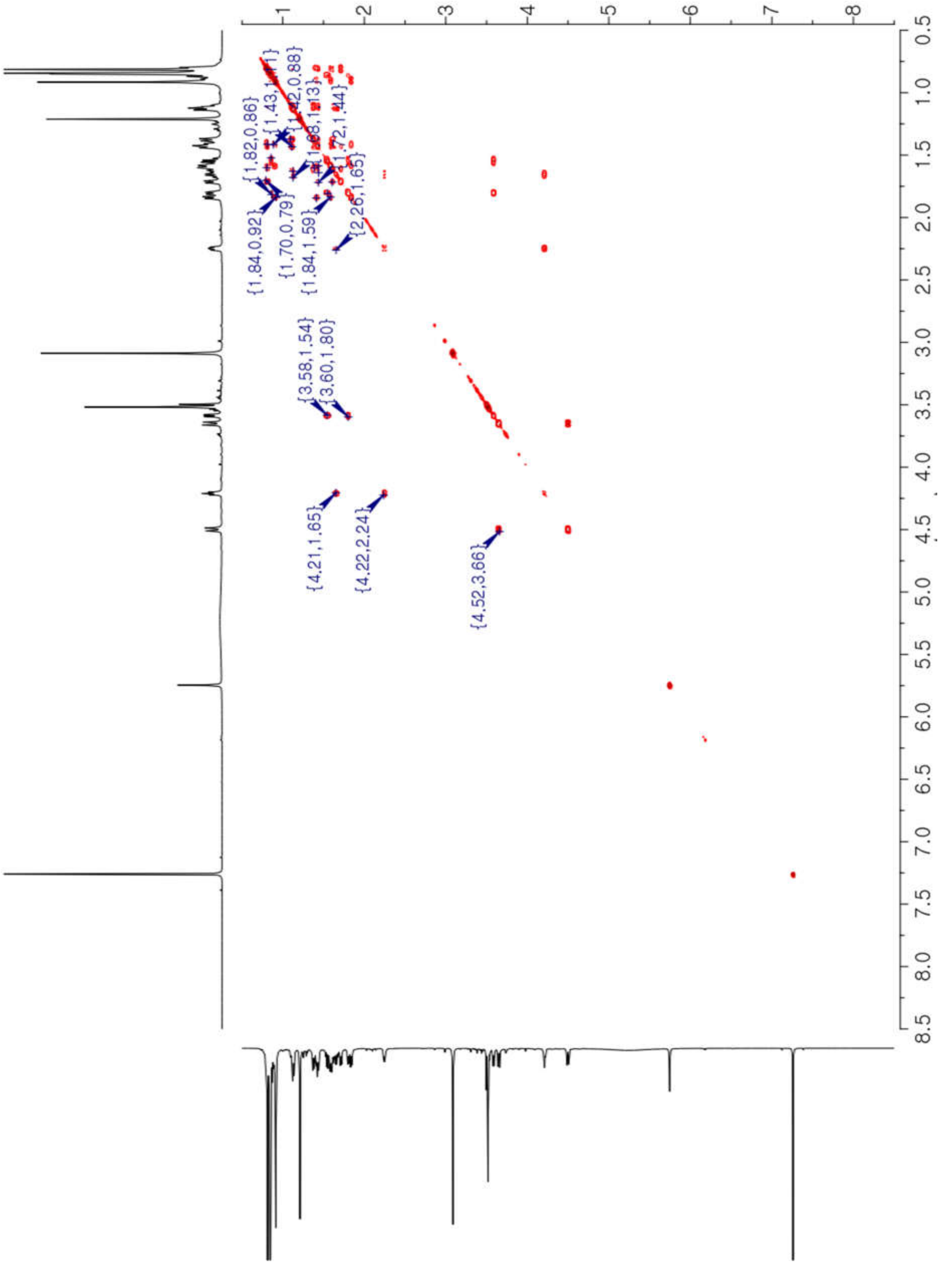

Figure S31. The COSY (600 MHz, $CDCl_3$) spectrum of **5**

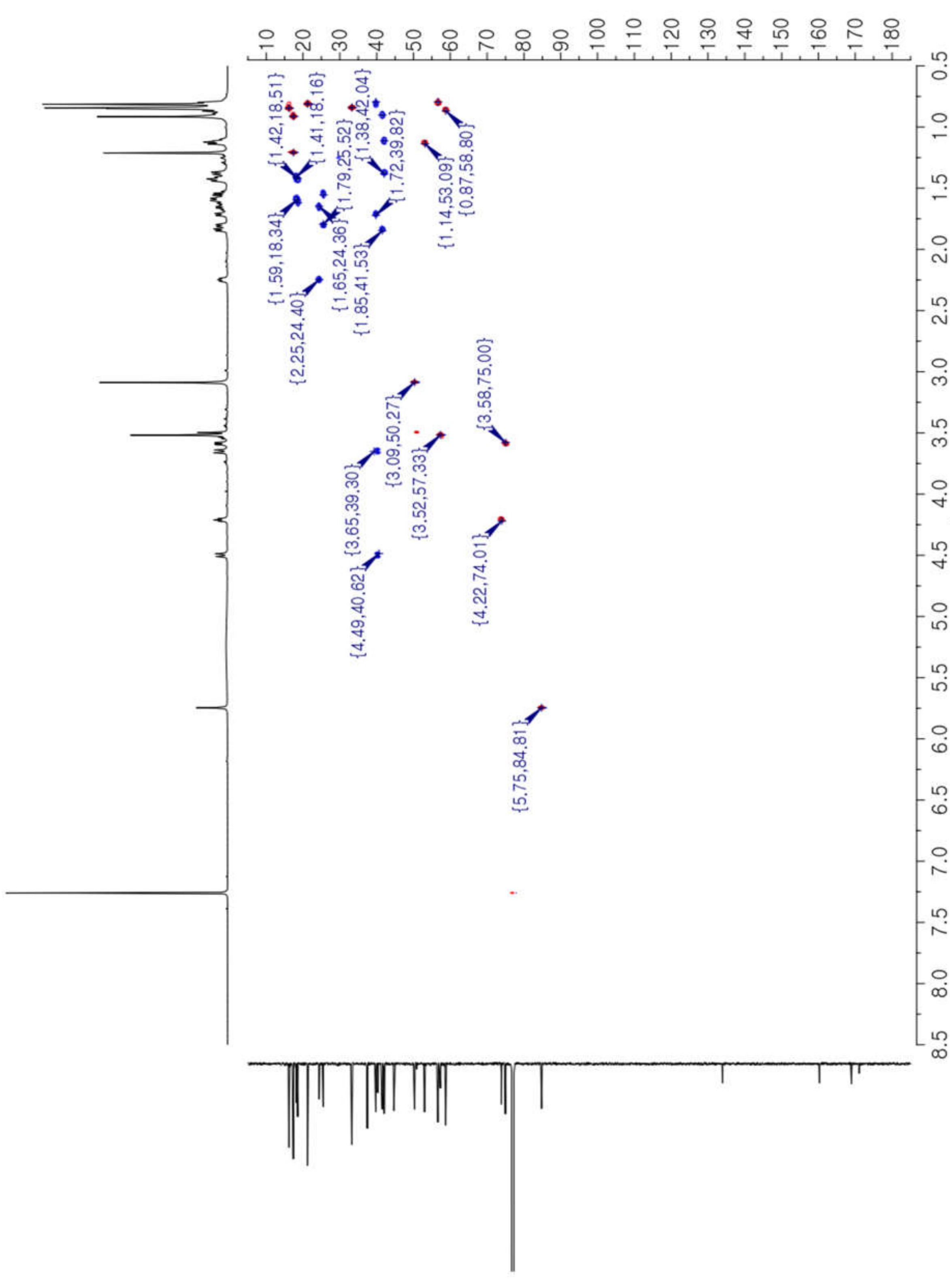

Figure S32. The eHSQC (600 MHz, $CDCl_3$) spectrum of **5**

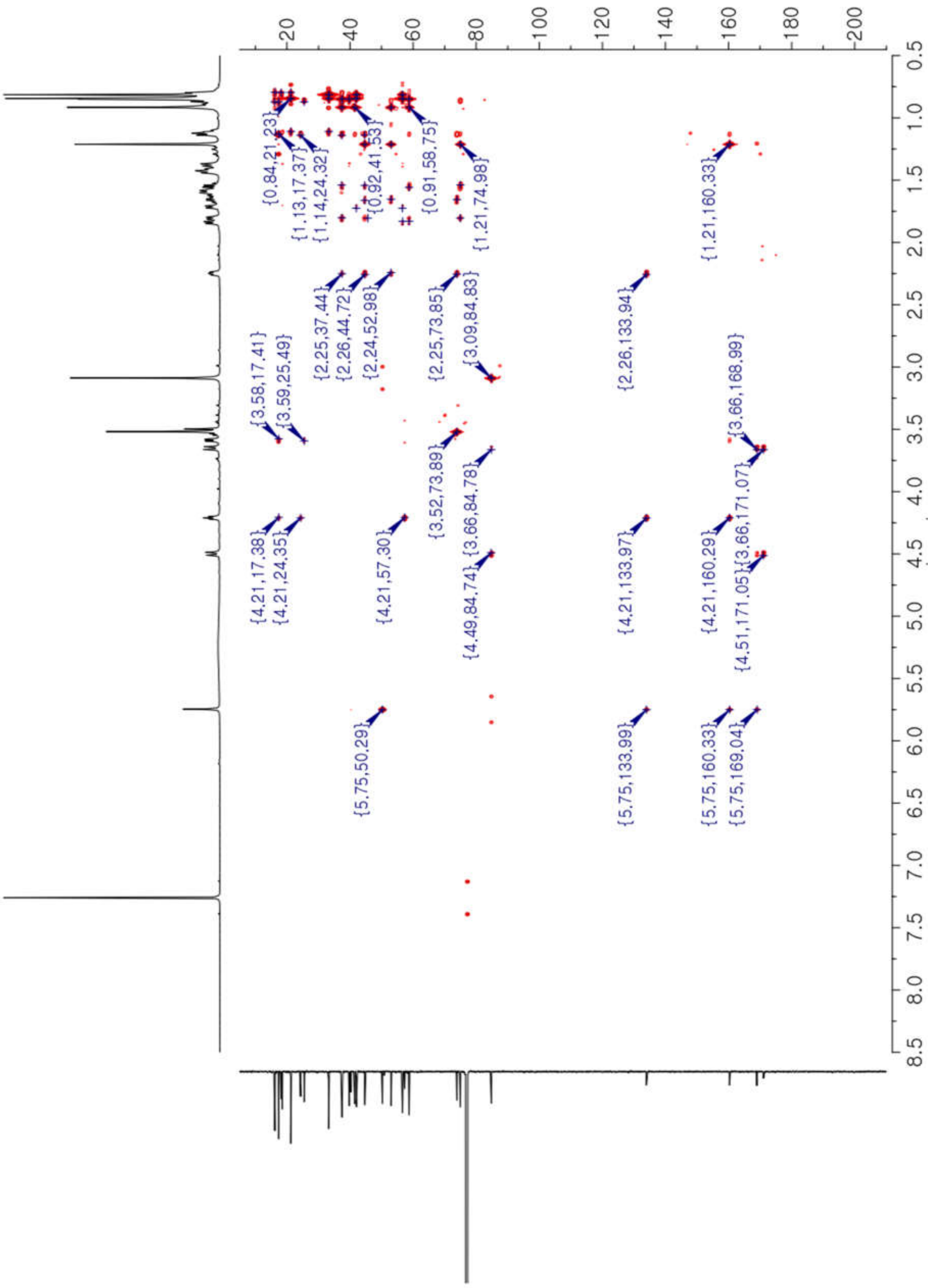

Figure S33. The HMBC (600 MHz, $CDCl_3$) spectrum of **5**

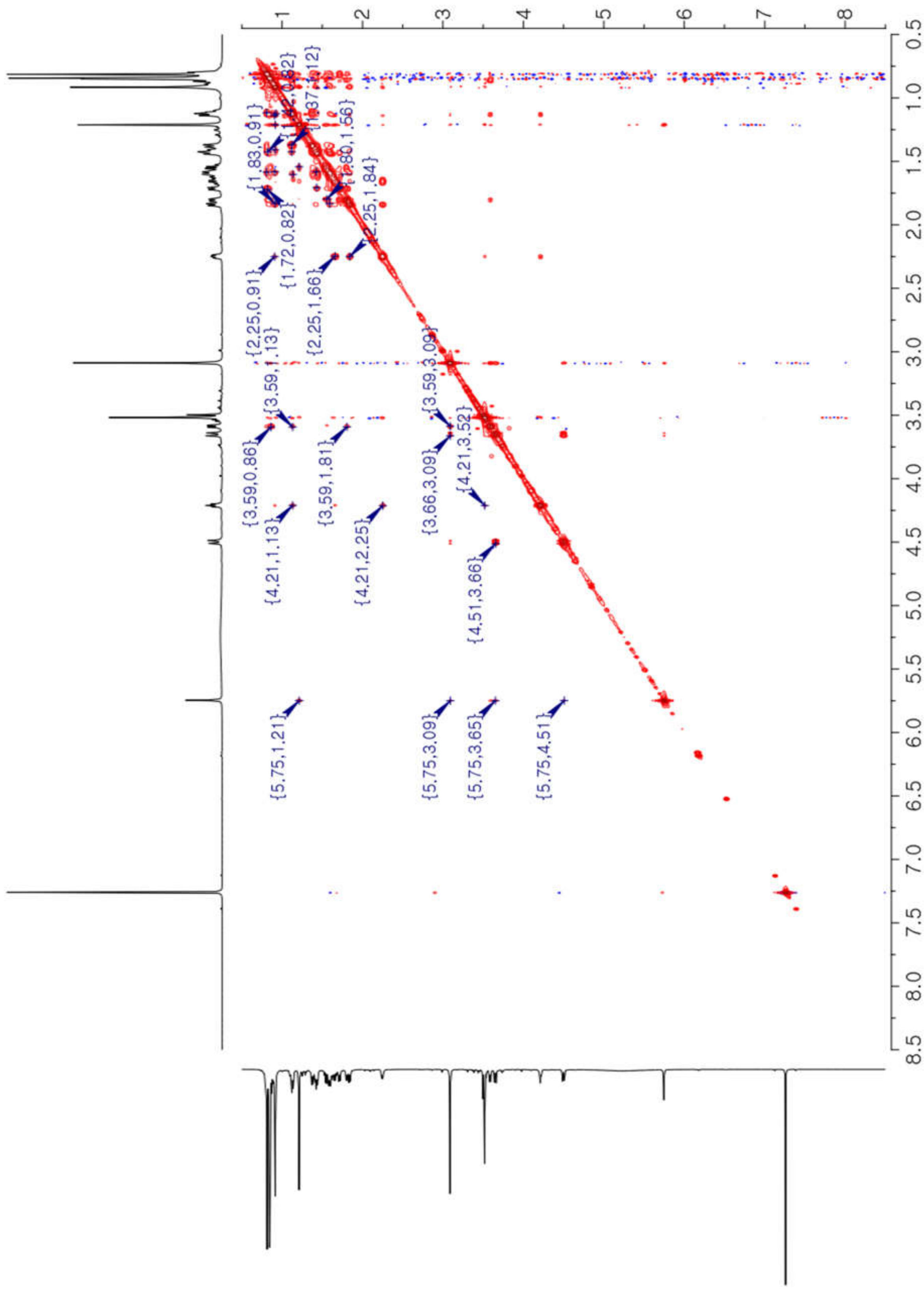

Figure S34. The NOESY (600 MHz, $CDCl_3$) spectrum of **5**

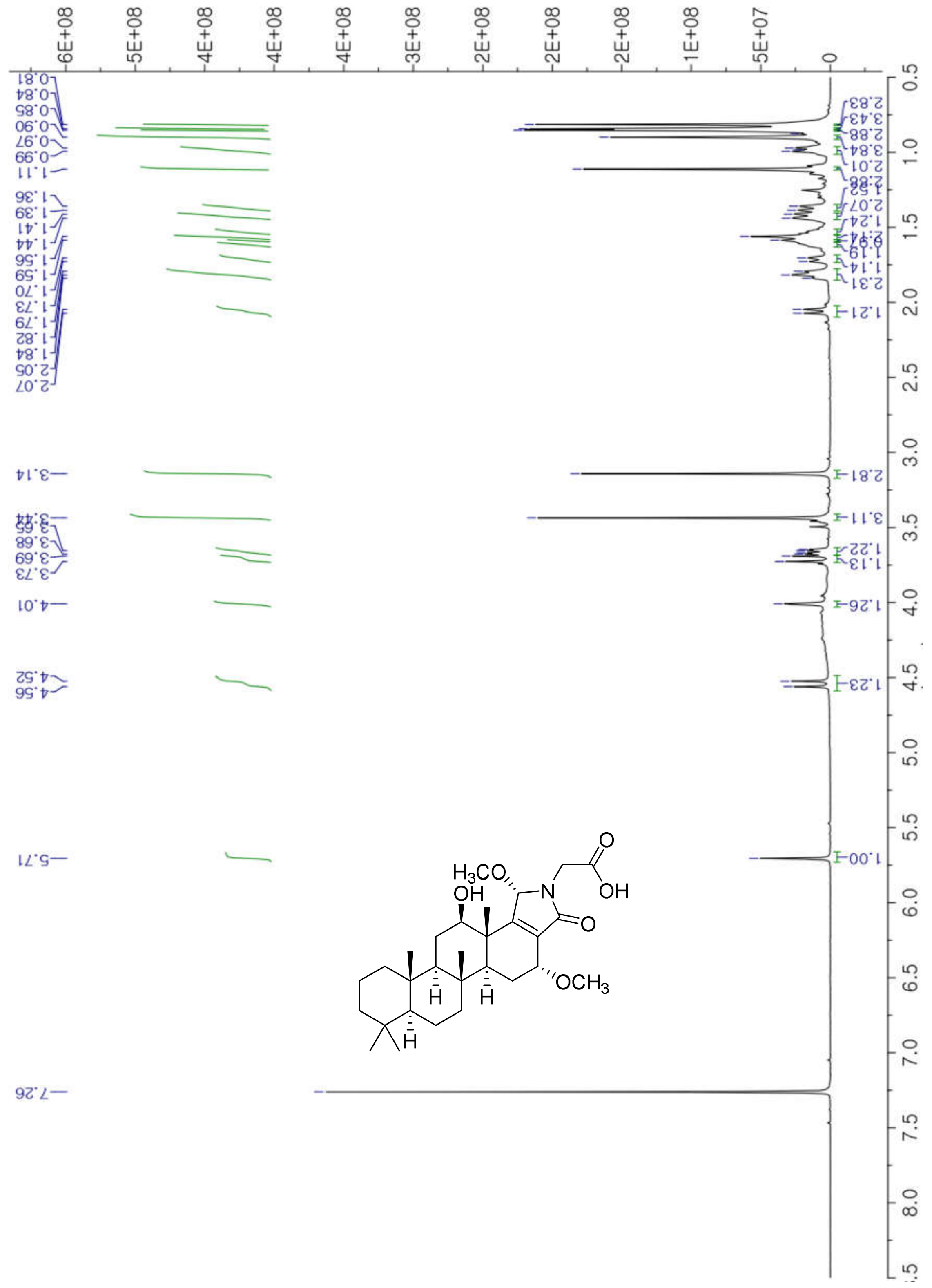

Figure S35. The ^1H NMR (600 MHz, $CDCl_3$) spectrum of **6**

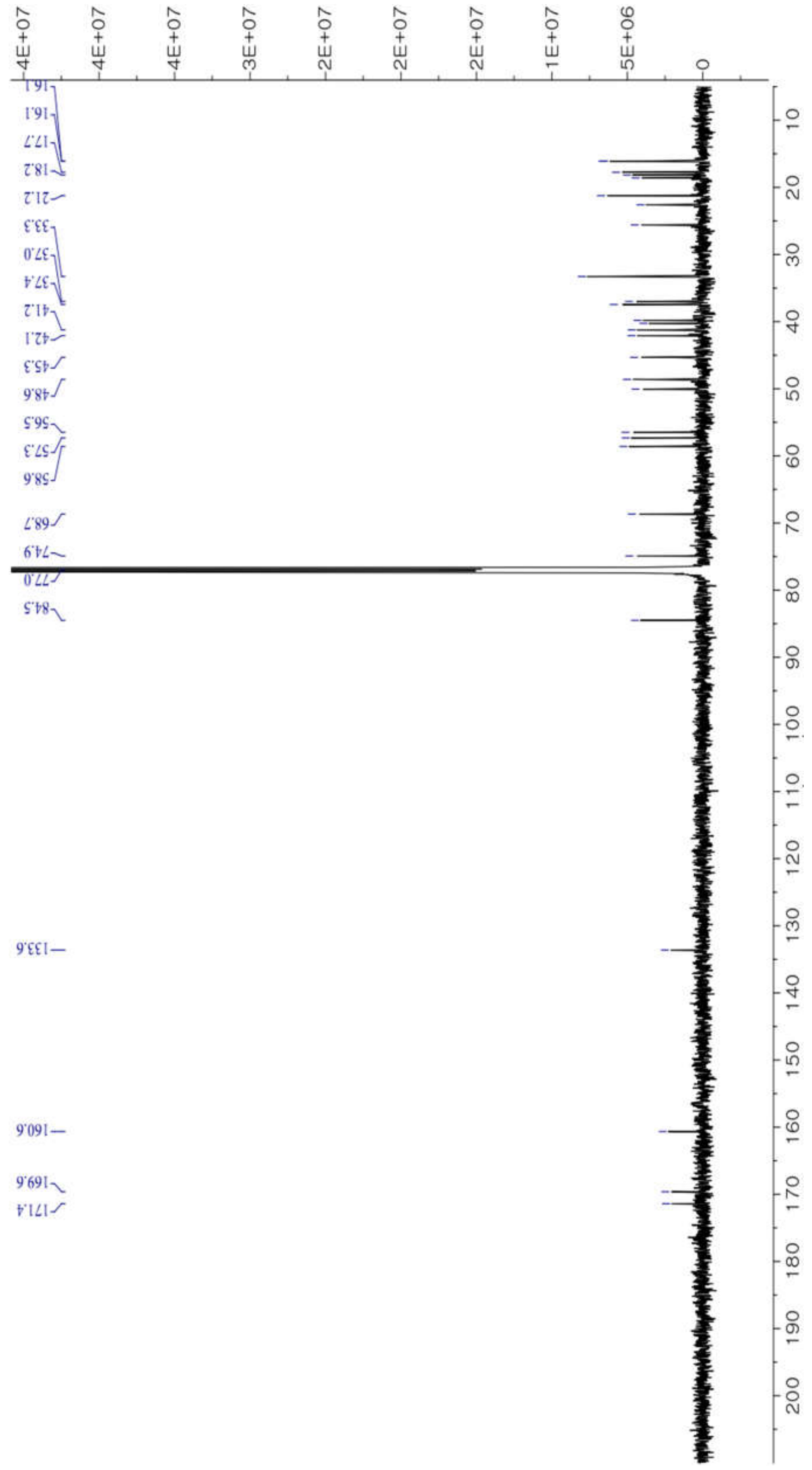

Figure S36. The ^{13}C NMR (150 MHz, $CDCl_3$) spectrum of **6**

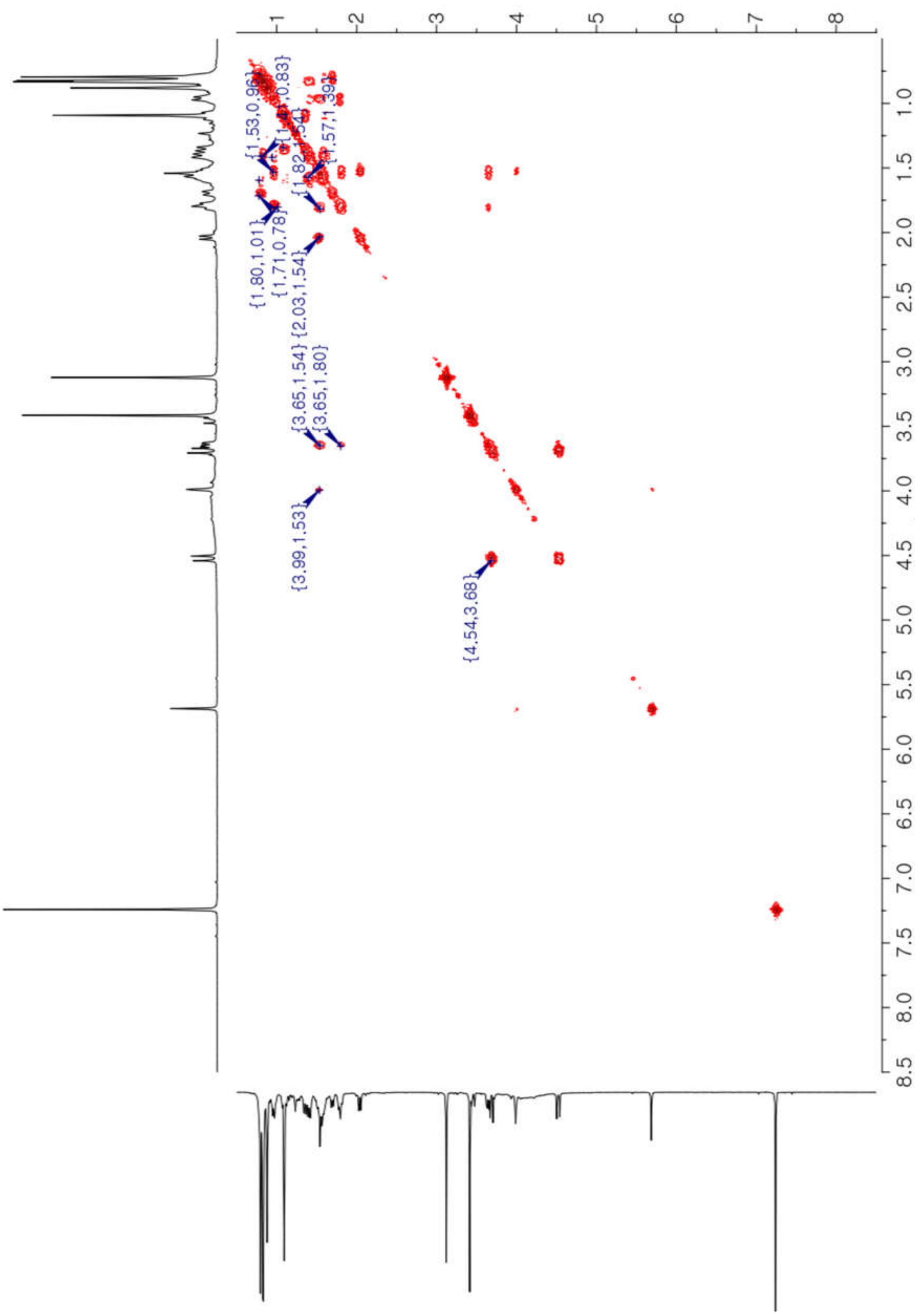

Figure S37. The COSY (600 MHz, $CDCl_3$) spectrum of **6**

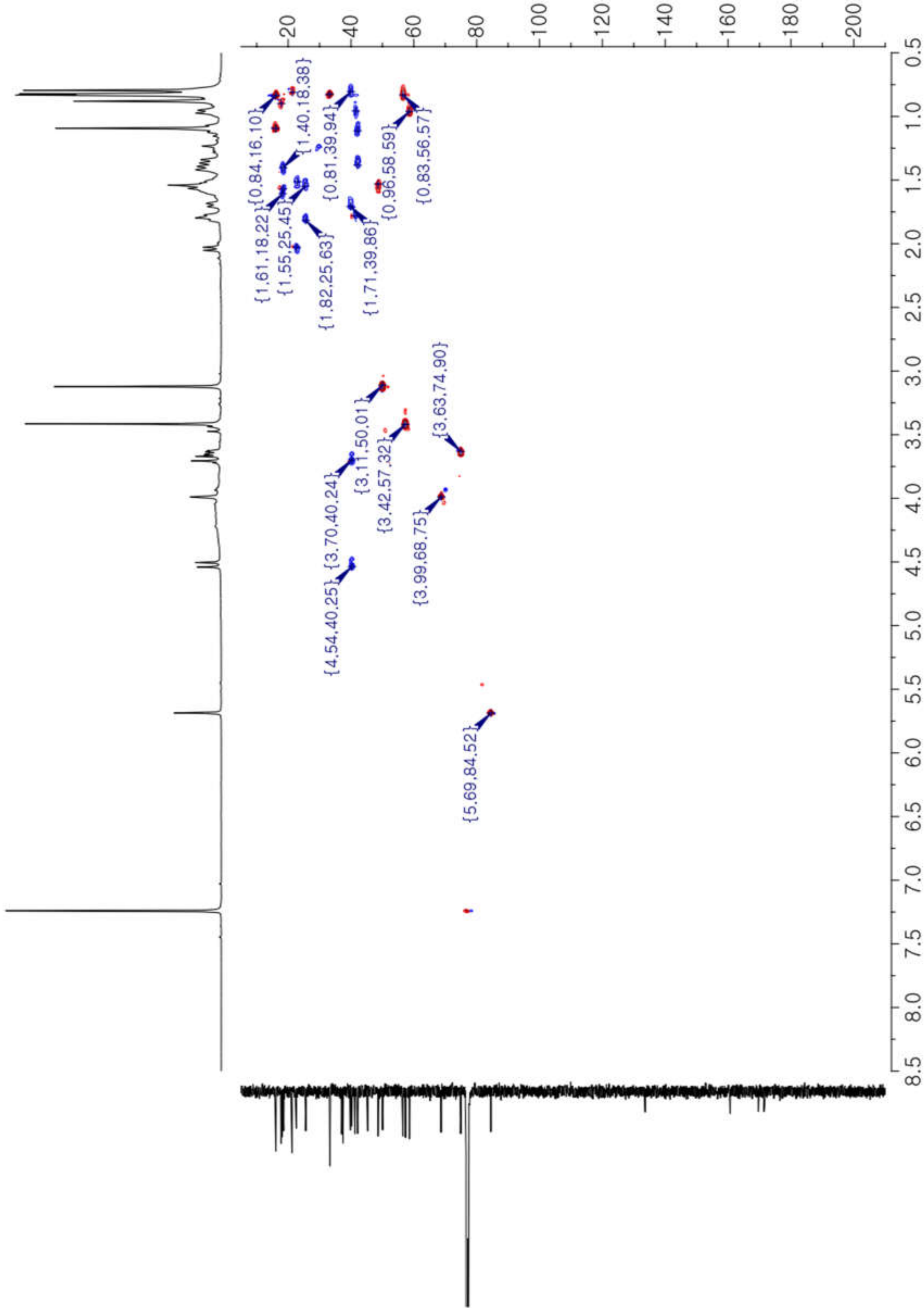

Figure S38. The eHSQC (600 MHz, $CDCl_3$) spectrum of **6**

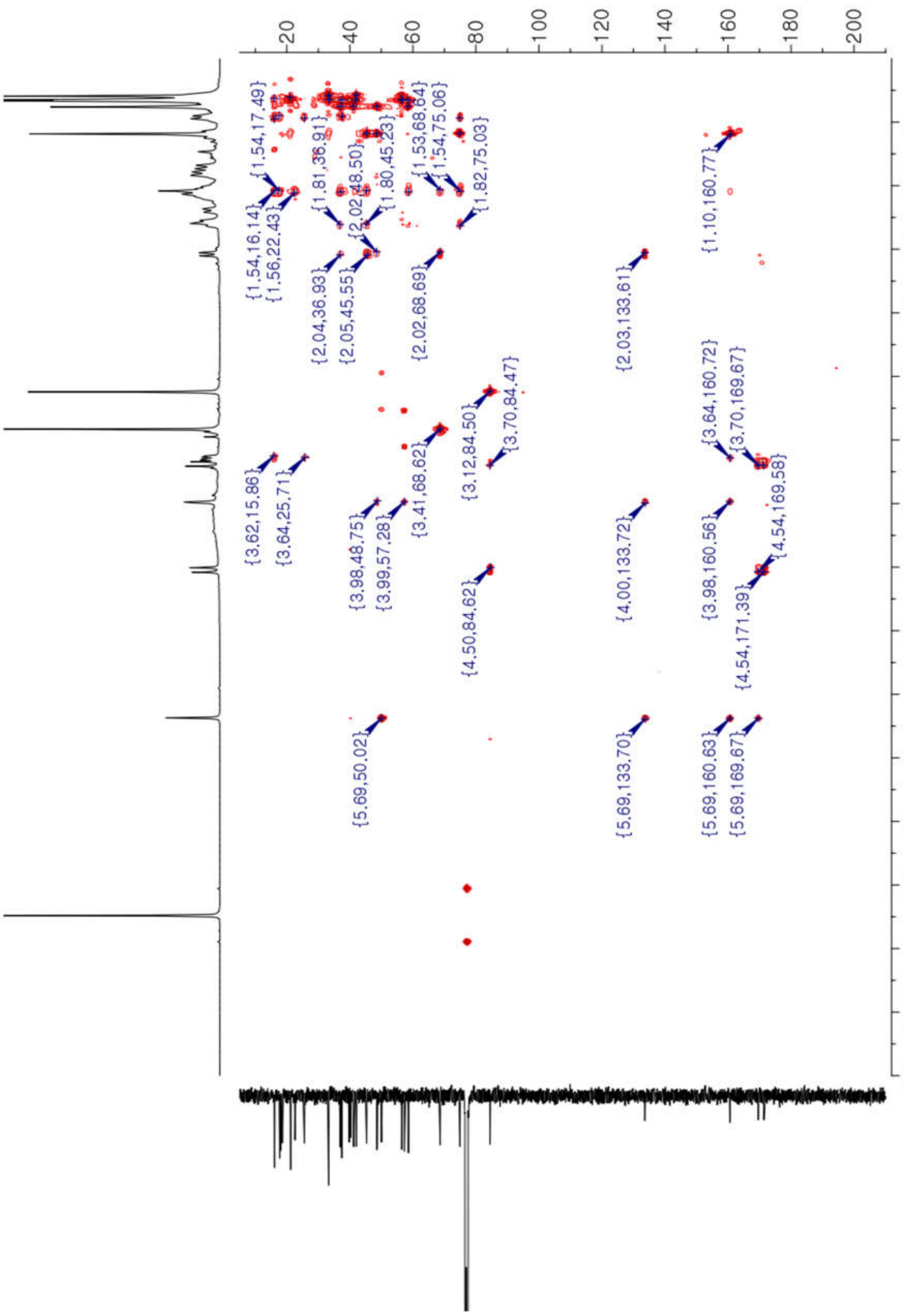

Figure S39. The HMBC (600 MHz, $CDCl_3$) spectrum of **6**

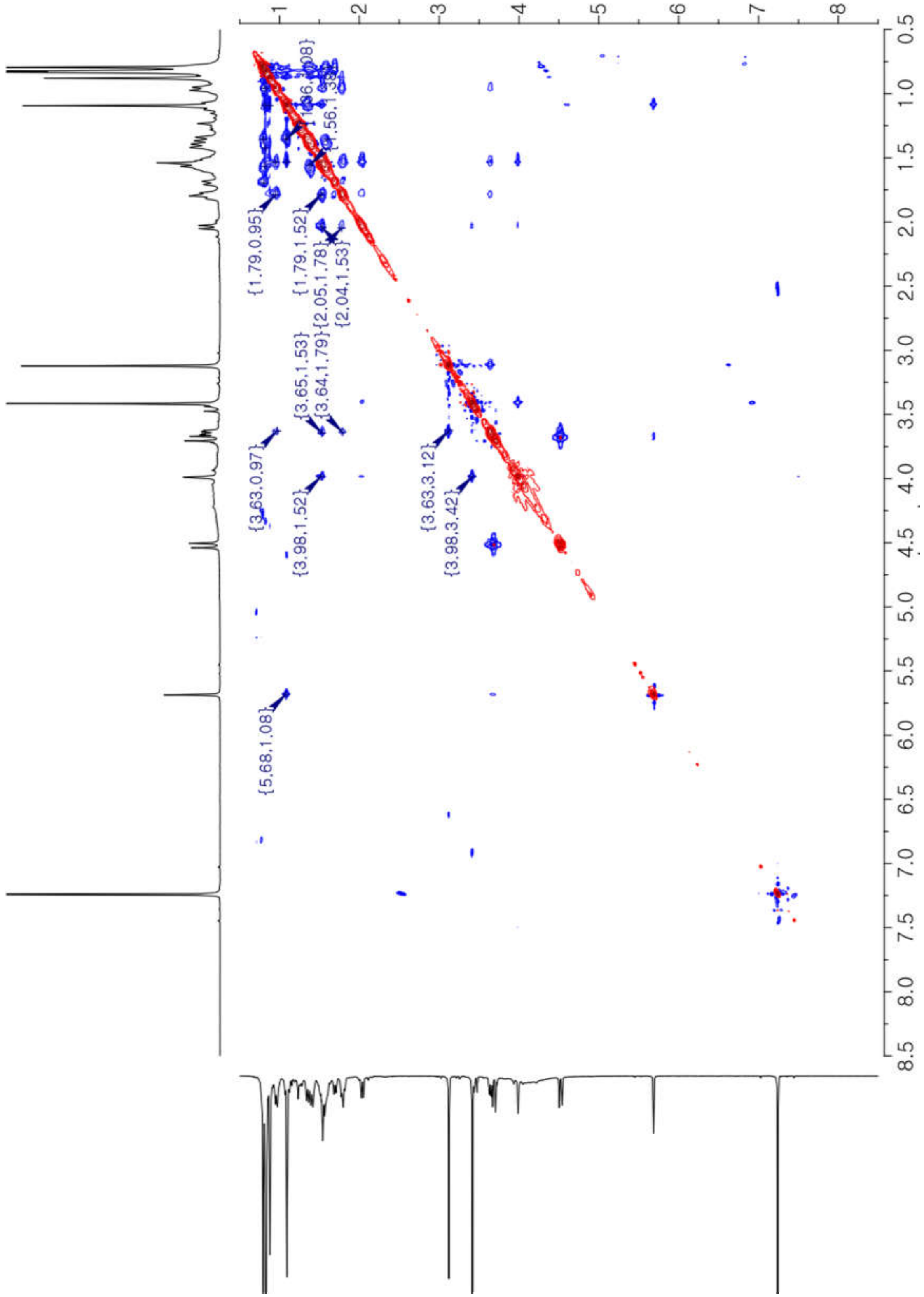

Figure S40. The NOESY (600 MHz, $CDCl_3$) spectrum of **6**

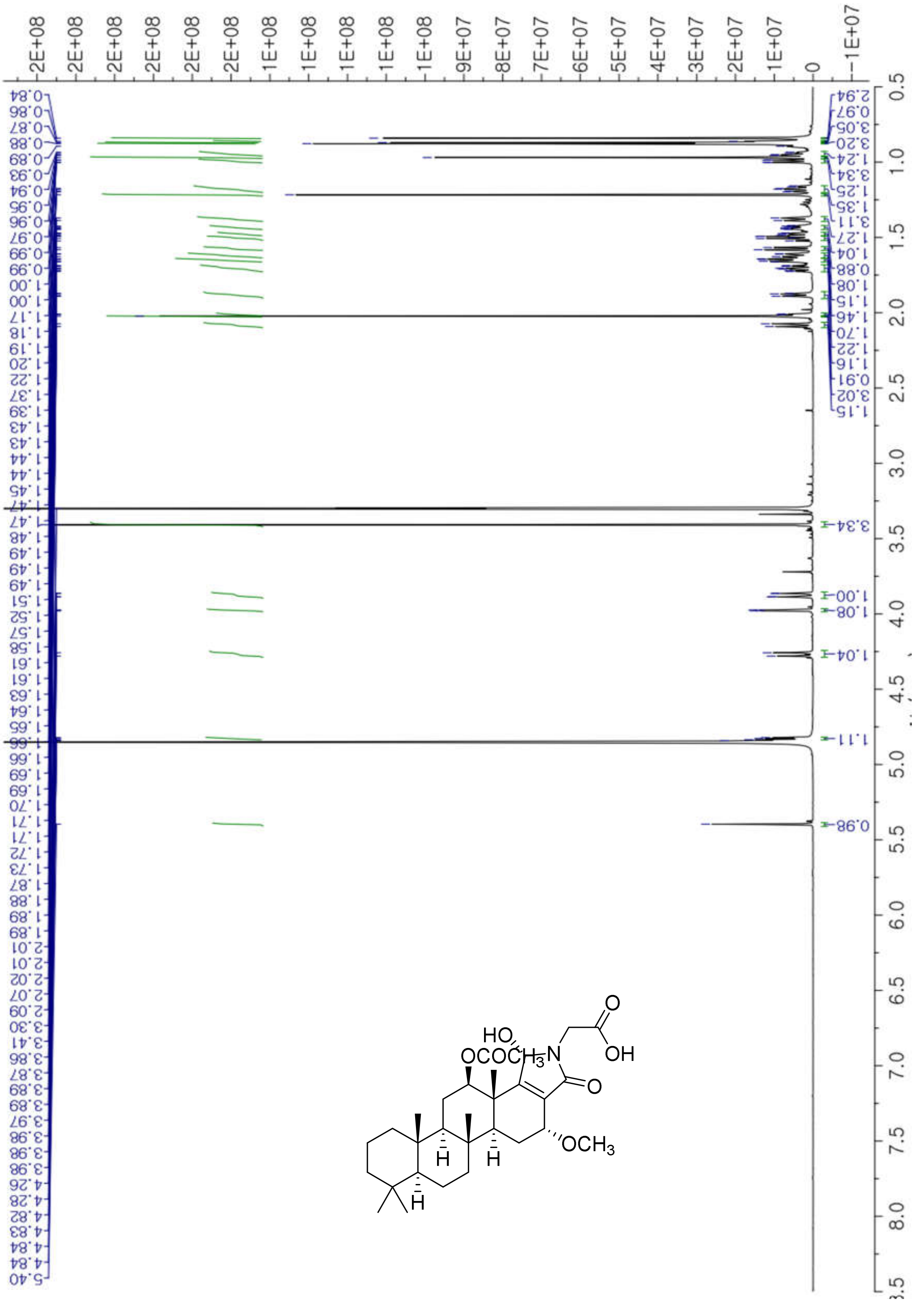

Figure S41. The ^{1}H NMR (600 MHz, MeOH-d_4) spectrum of **7**

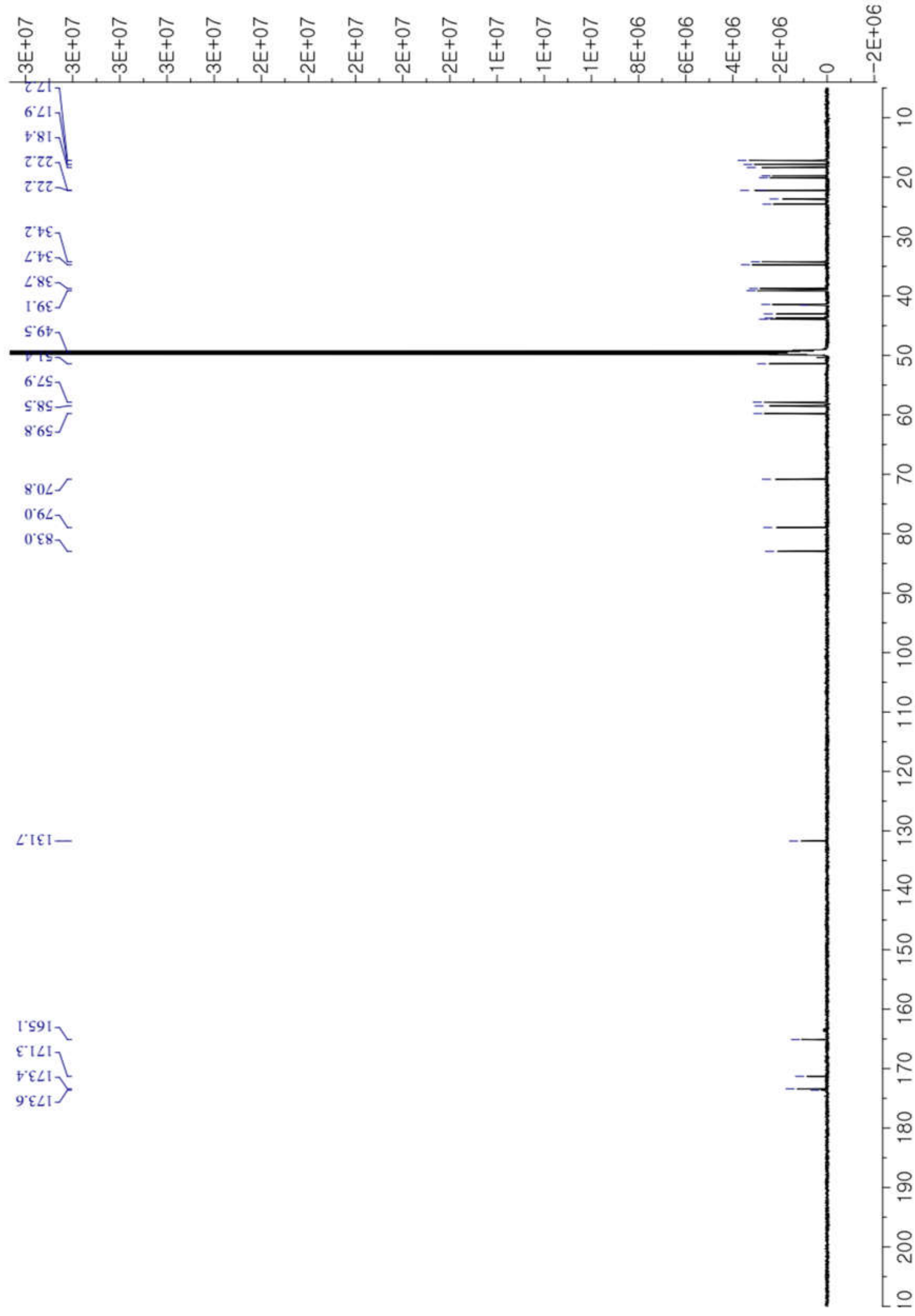

Figure S42. The ^{13}C NMR (150 MHz, MeOH-d_4) spectrum of **7**

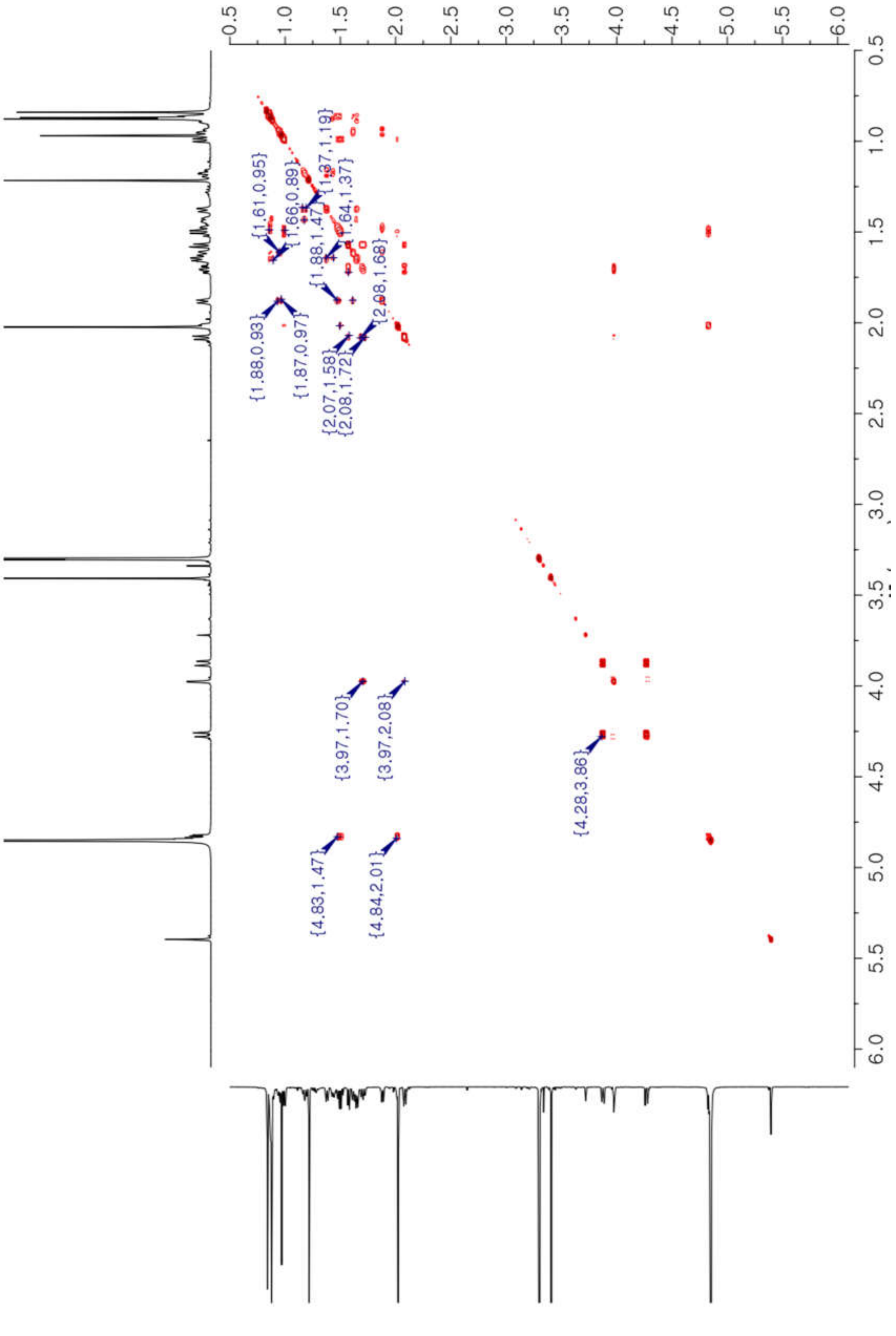

Figure S43. The COSY (600 MHz, MeOH-d_4) spectrum of **7**

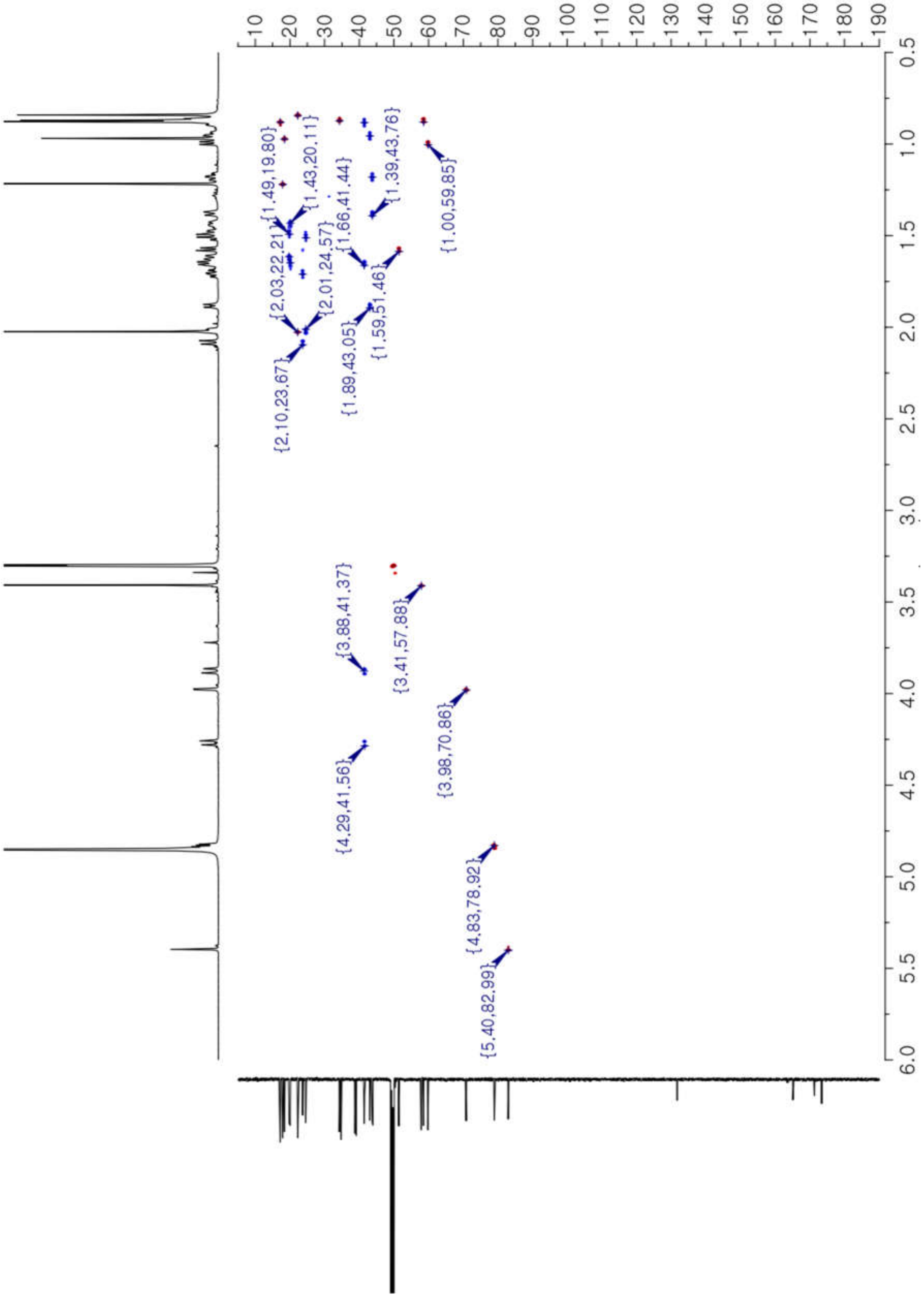

Figure S44. The eHSQC (600 MHz, MeOH-d_4) spectrum of **7**

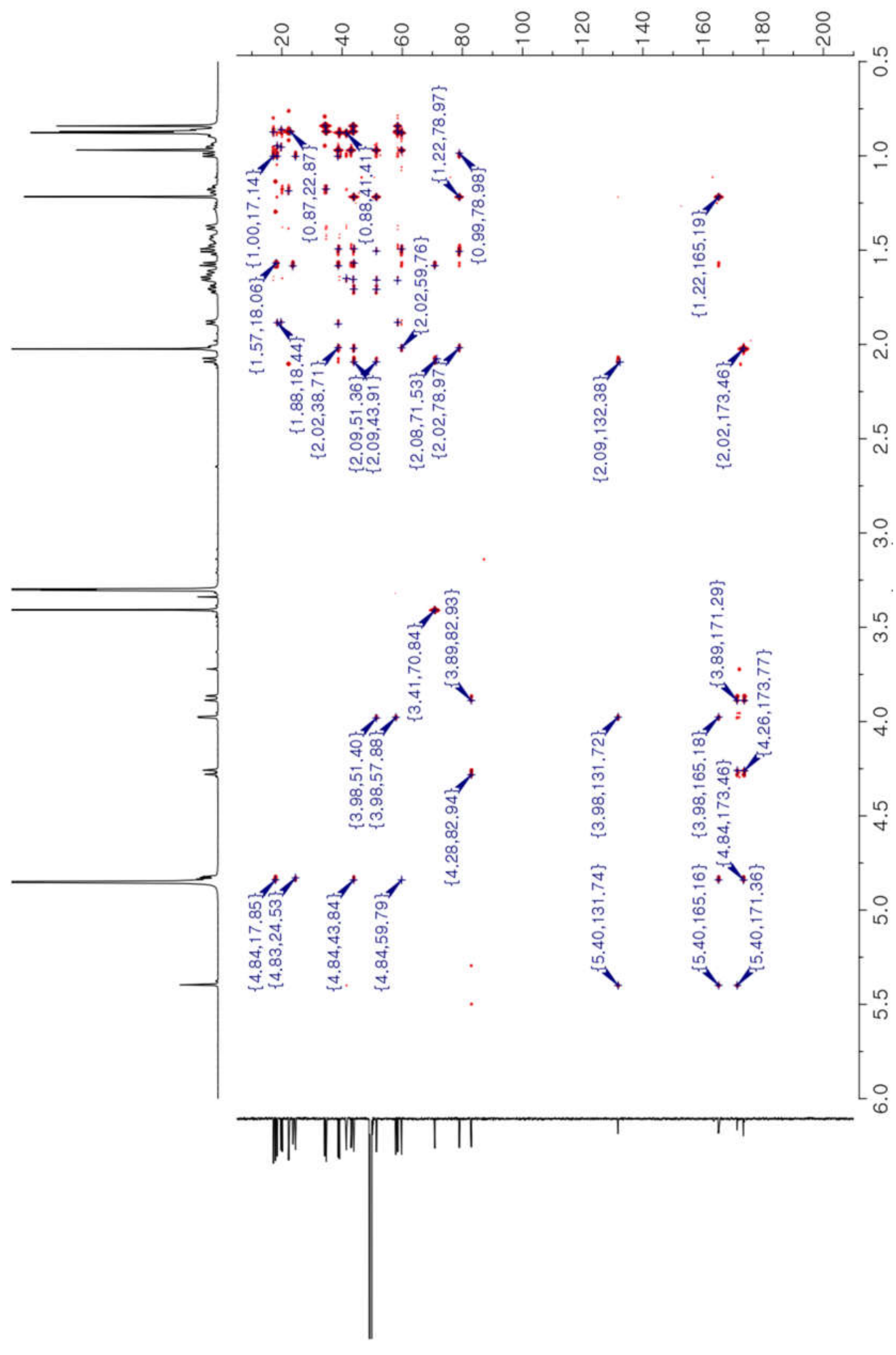

Figure S45. The HMBC (600 MHz, MeOH-d_4) spectrum of **7**

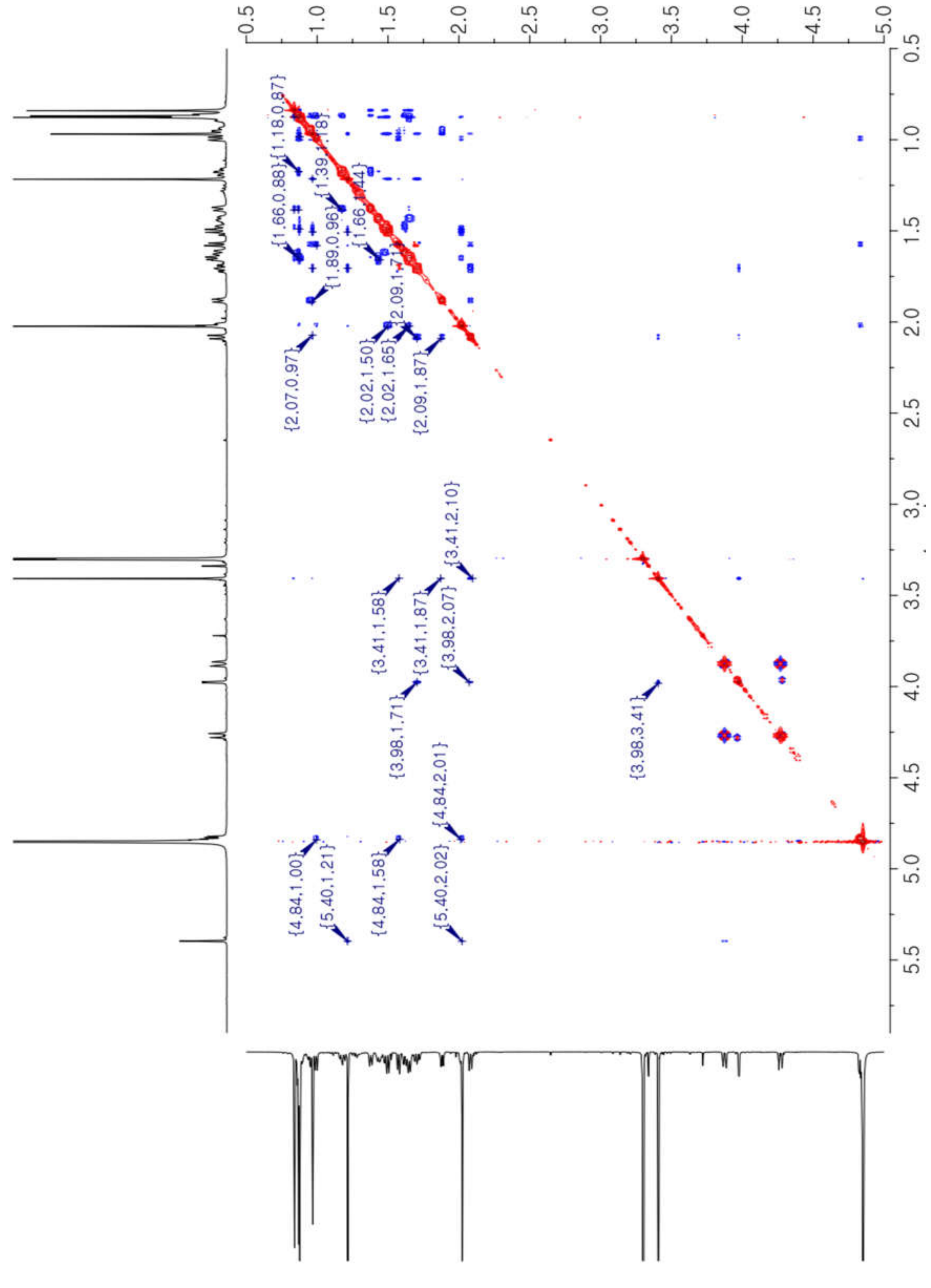

Figure S46. The NOESY (600 MHz, MeOH-d_4) spectrum of **7**

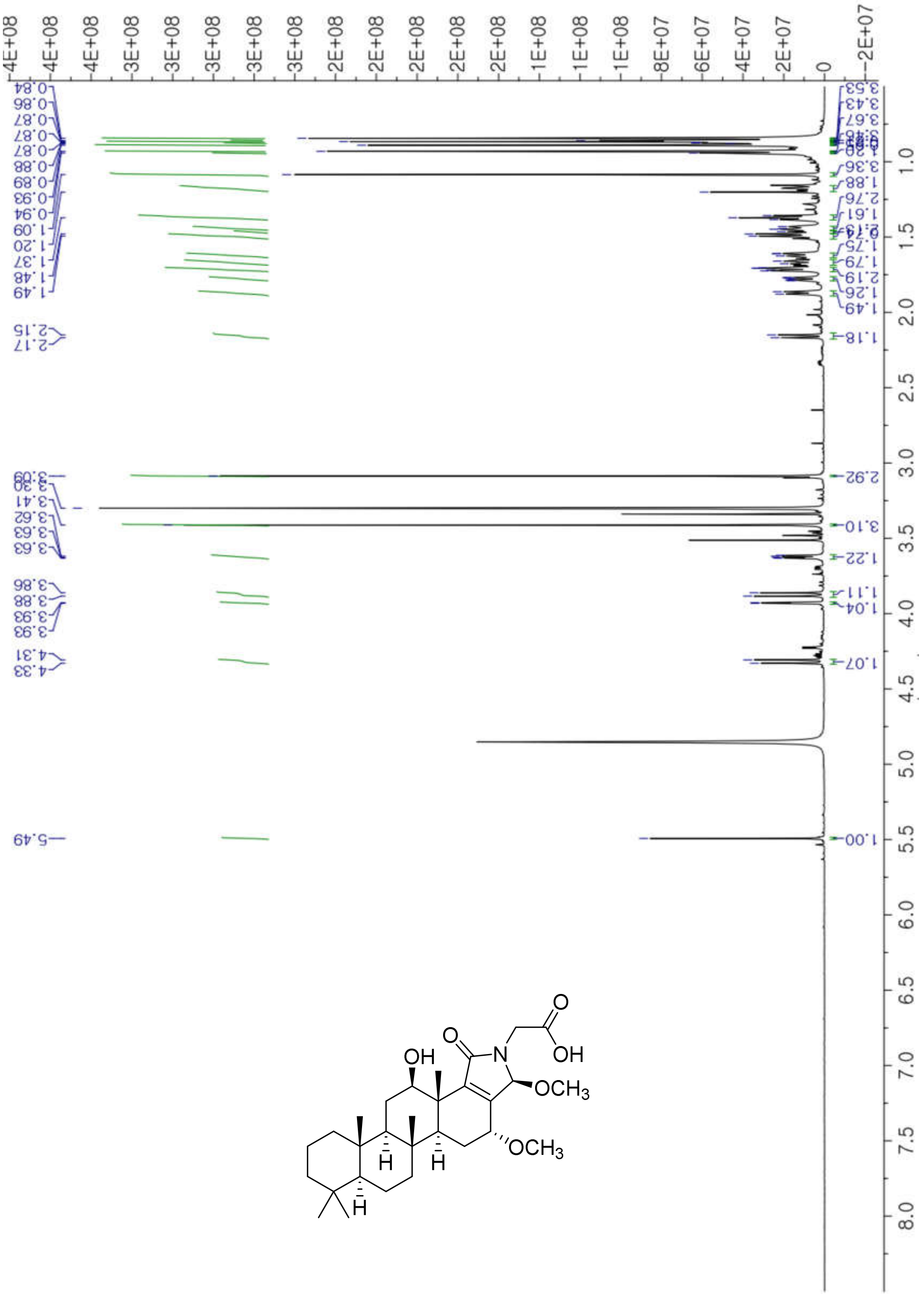

Figure S47. The ^{1}H NMR (600 MHz, MeOH-d_4) spectrum of **8**

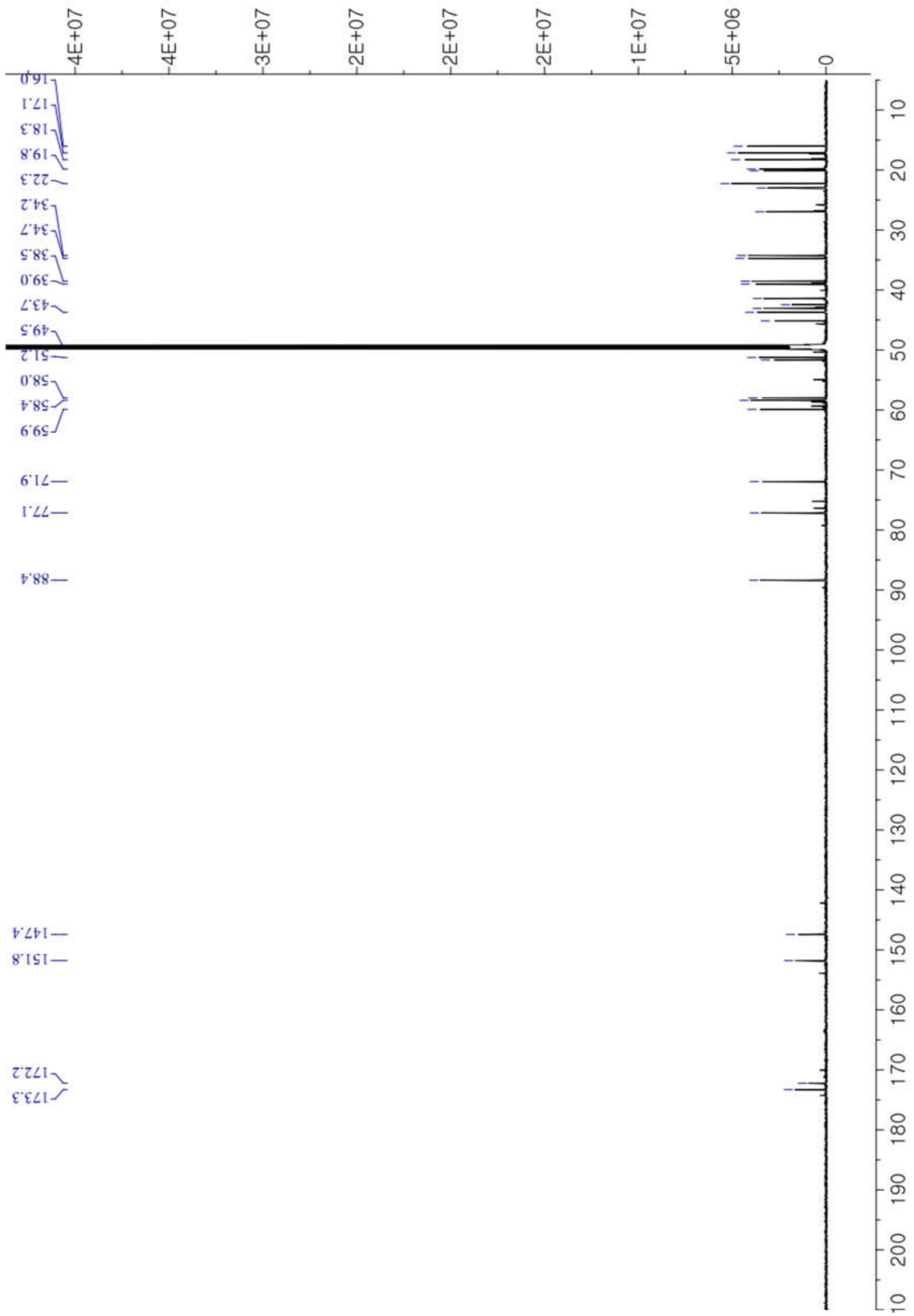

Figure S48. The ^{13}C NMR (150 MHz, MeOH-d_4) spectrum of **8**

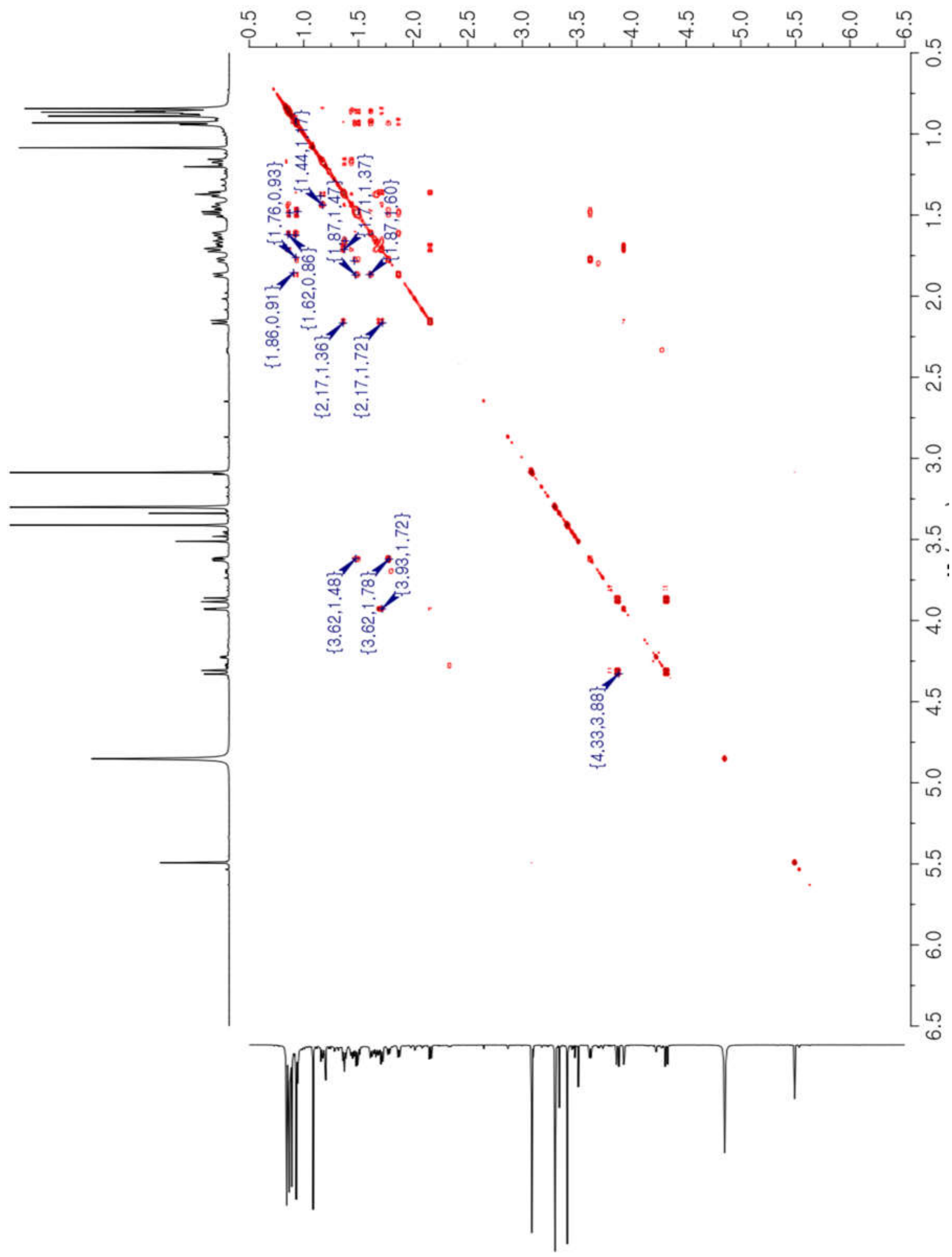

Figure S49. The COSY (600 MHz, MeOH-d_4) spectrum of **8**

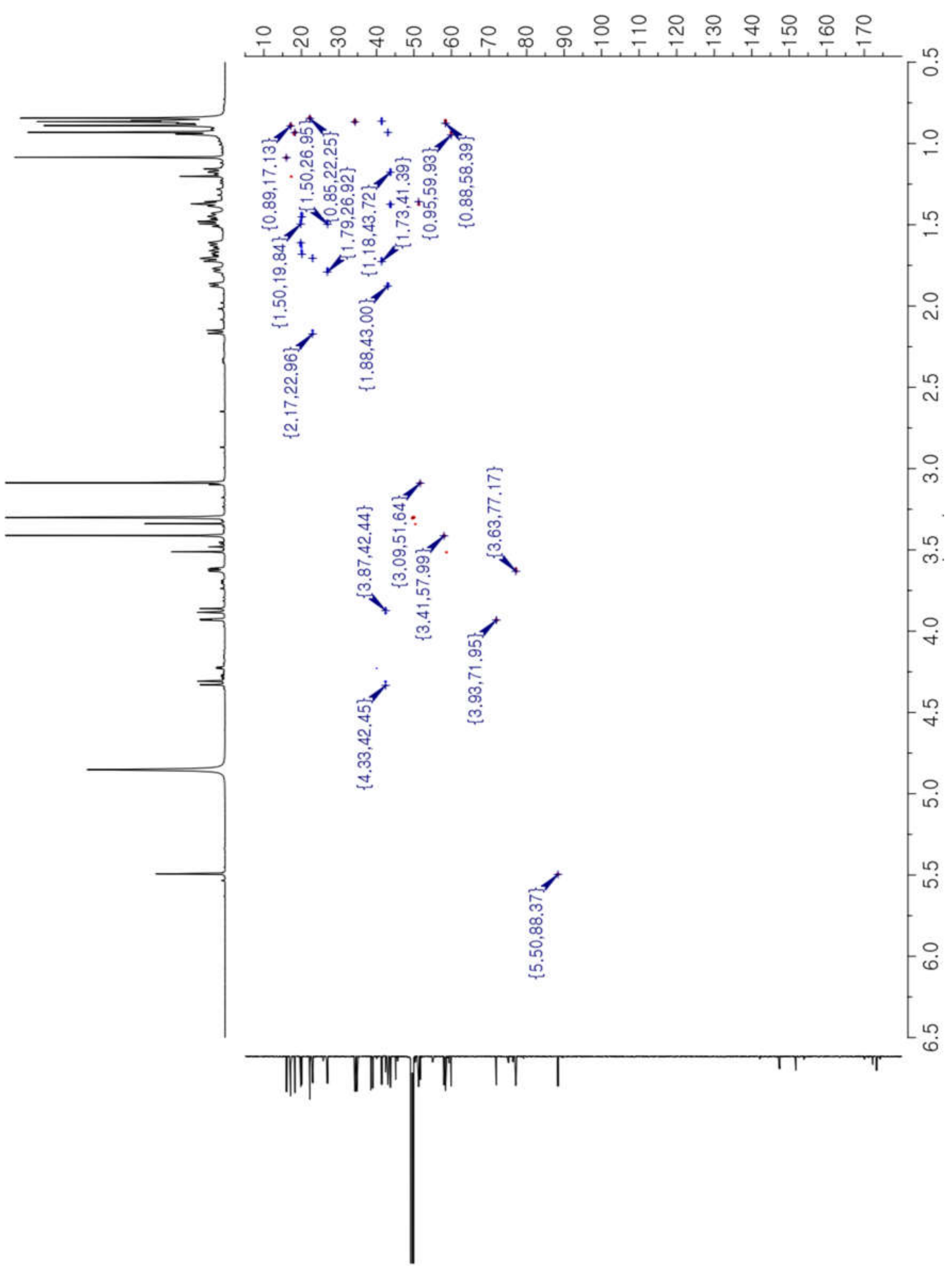

Figure S50. The eHSQC (600 MHz, MeOH-d_4) spectrum of **8**

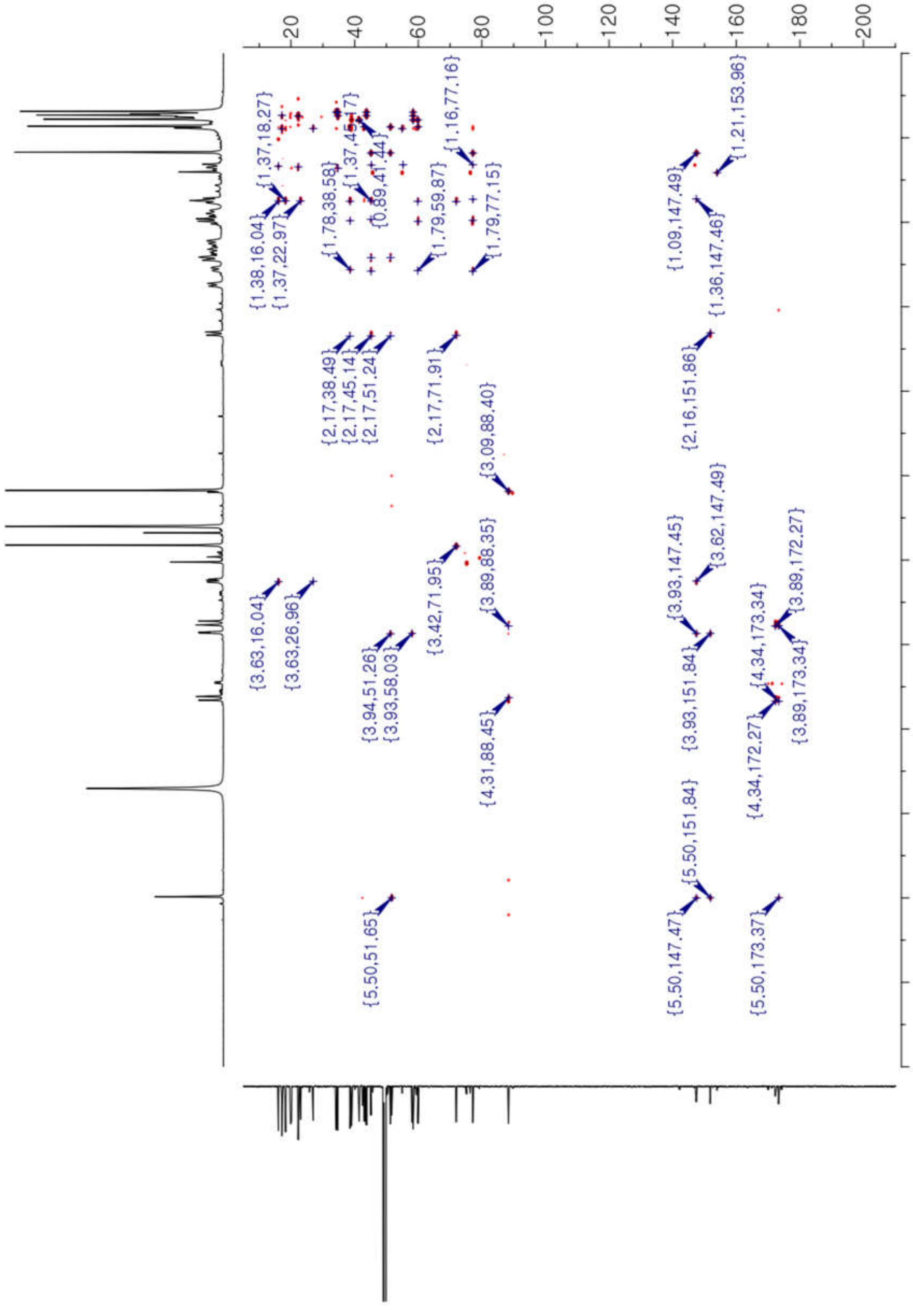

Figure S51. The HMBC (600 MHz, MeOH-d_4) spectrum of **8**

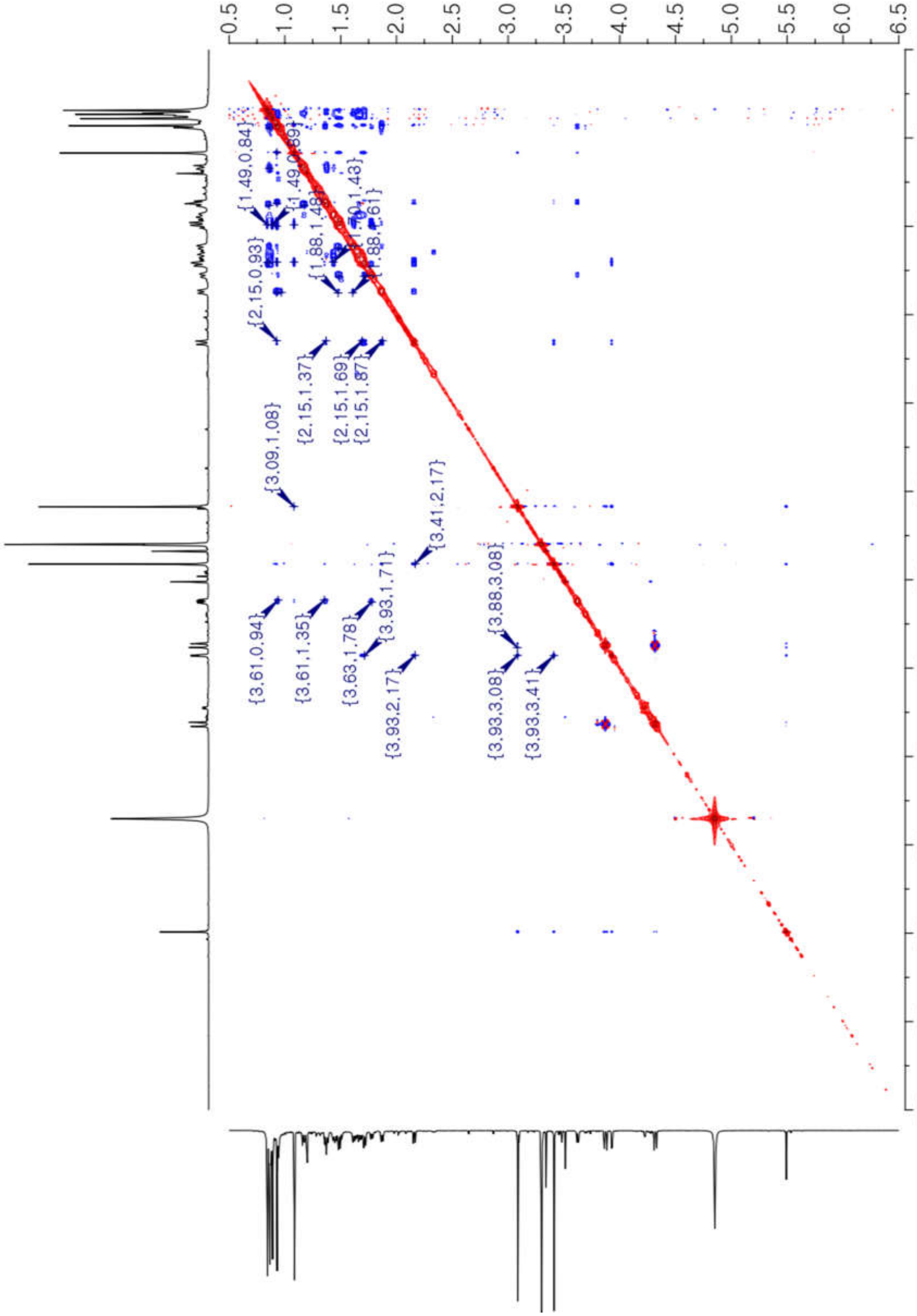

Figure S52. The NOESY (600 MHz, MeOH-d_4) spectrum of **8**

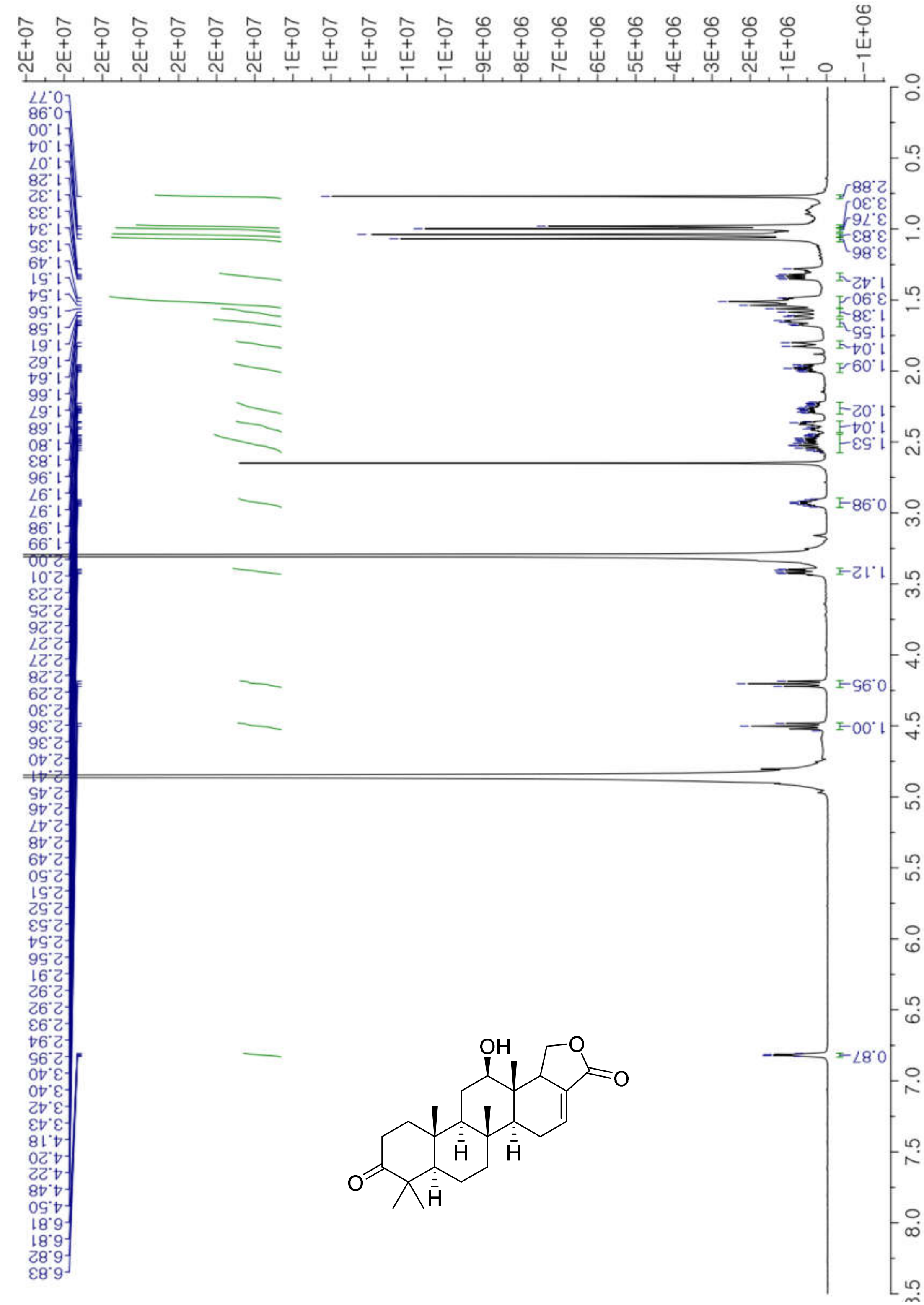

Figure S53. The ^{1}H NMR (600 MHz, MeOH-d_4) spectrum of **9**

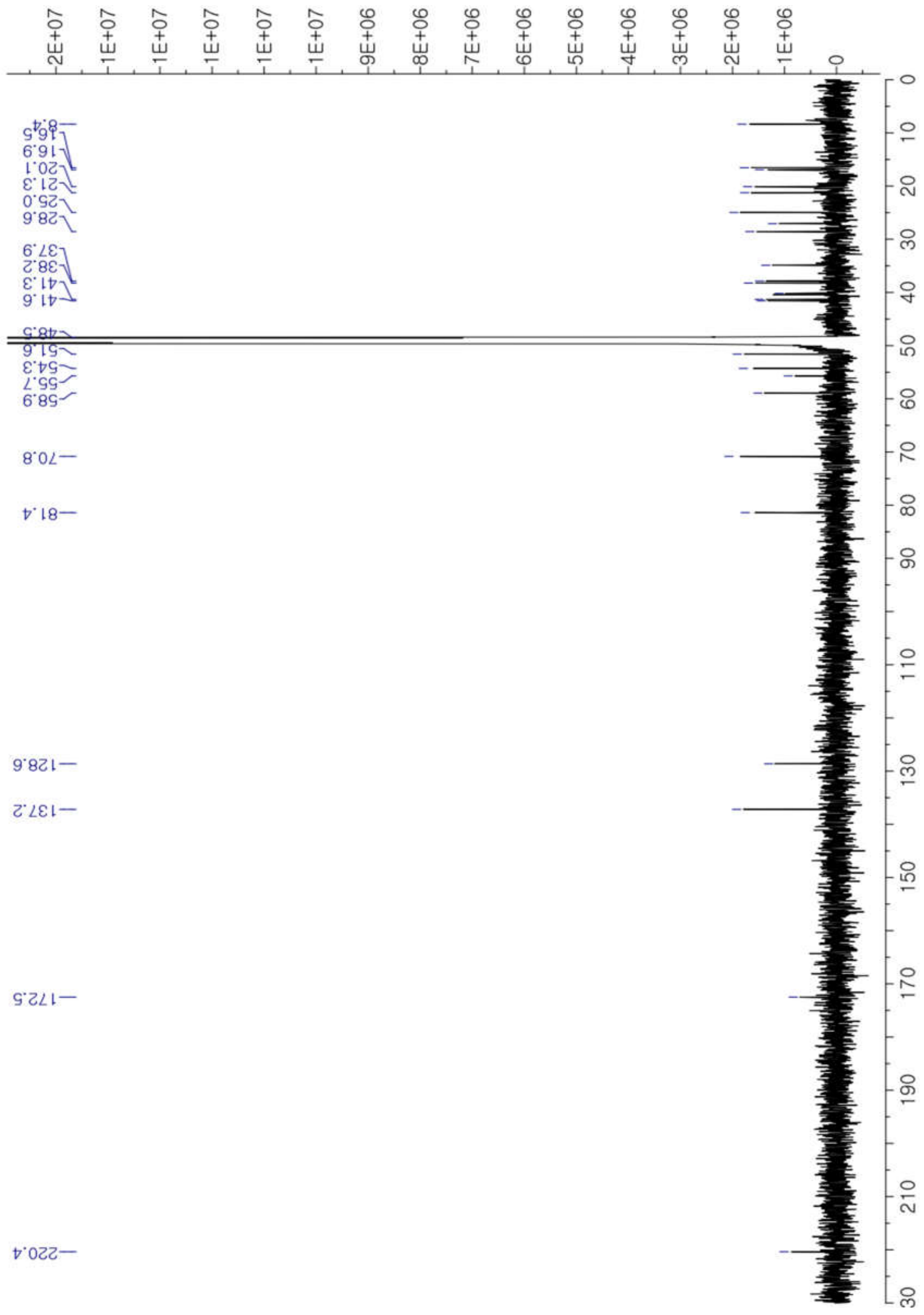

Figure S54. The ^{13}C NMR (150 MHz, MeOH-d_4) spectrum of **9**

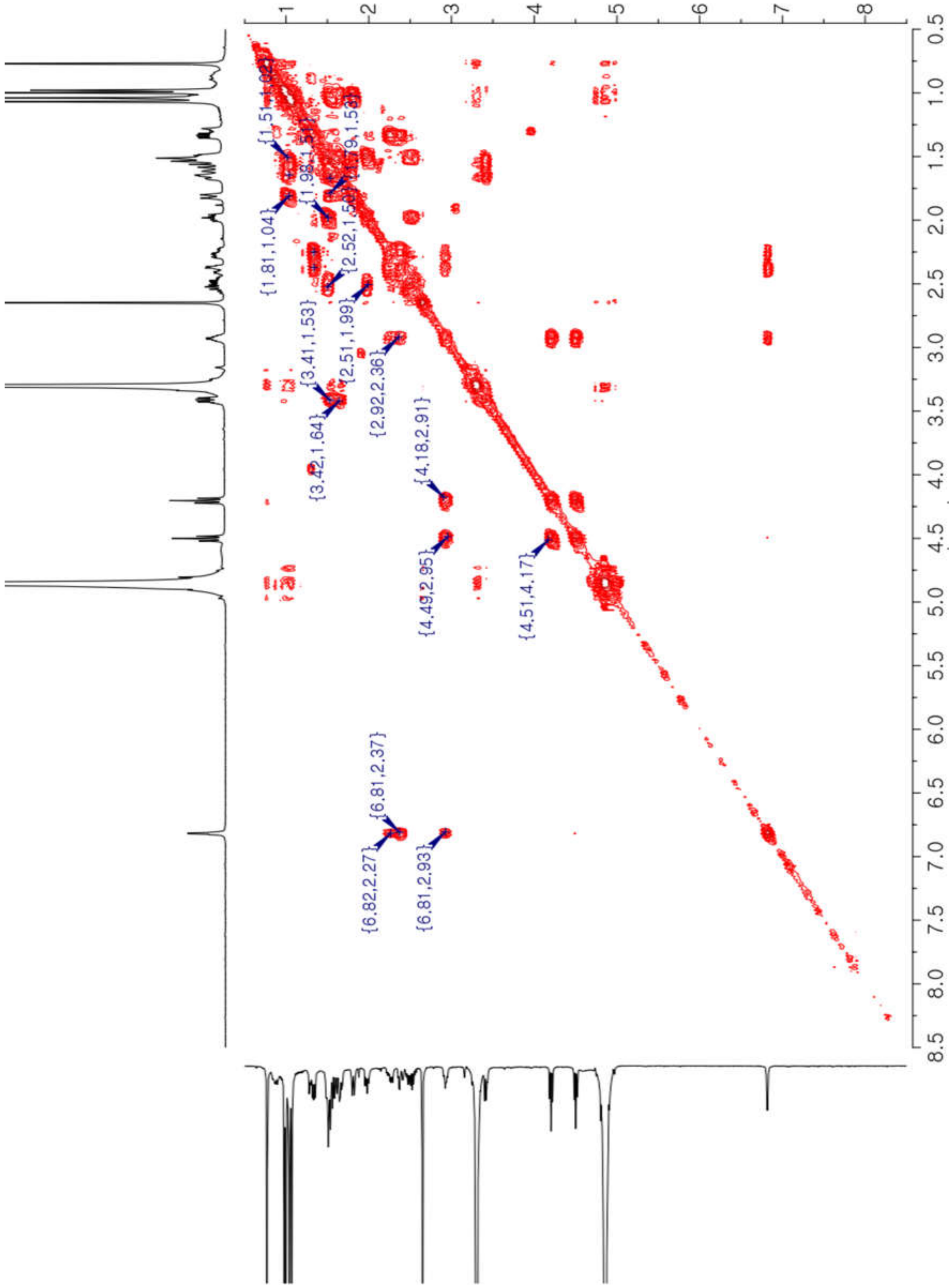

Figure S55. The COSY (600 MHz, MeOH-d_4) spectrum of **9**

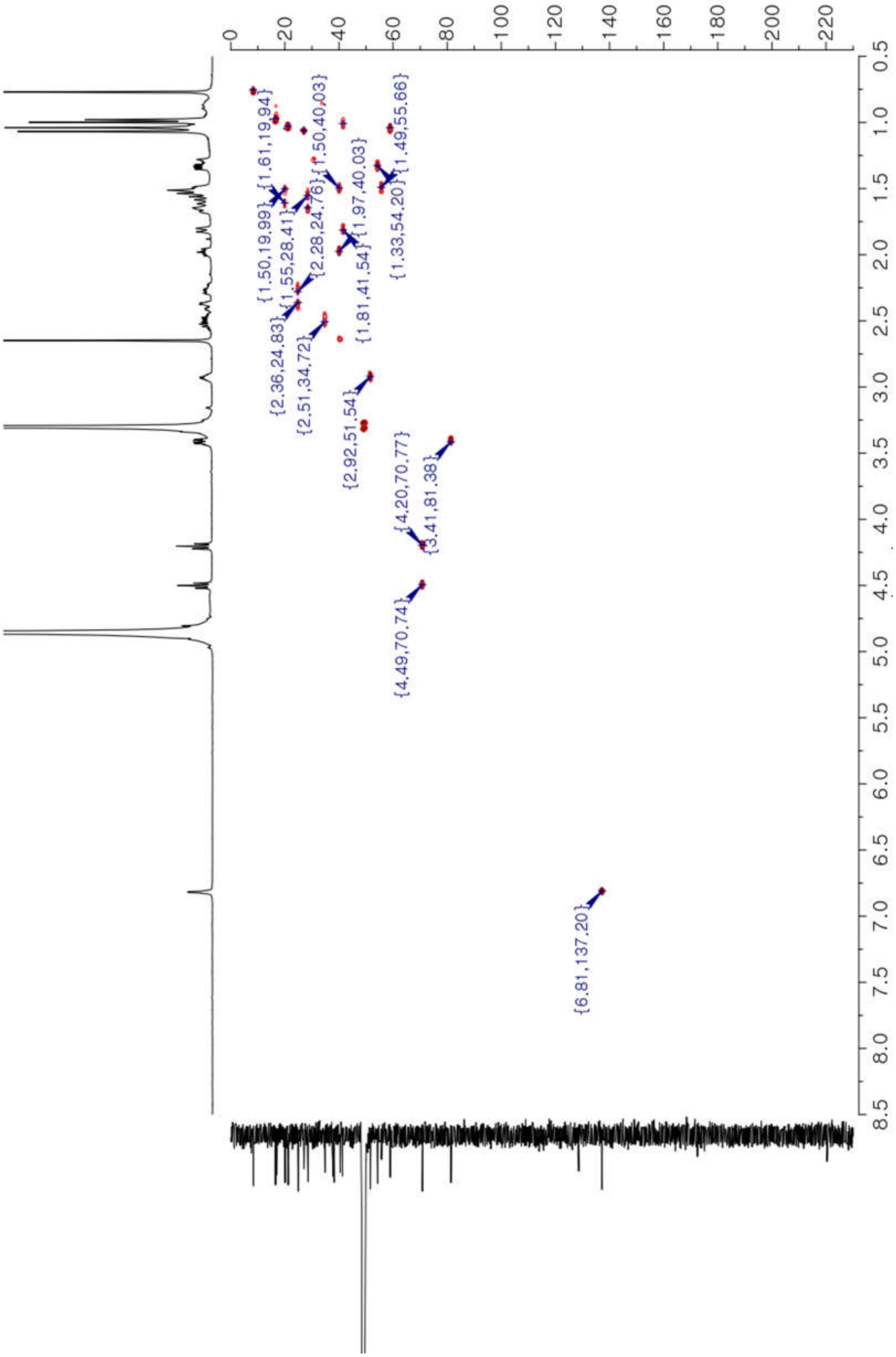

Figure S56. The HSQC (600 MHz, MeOH-d_4) spectrum of **9**

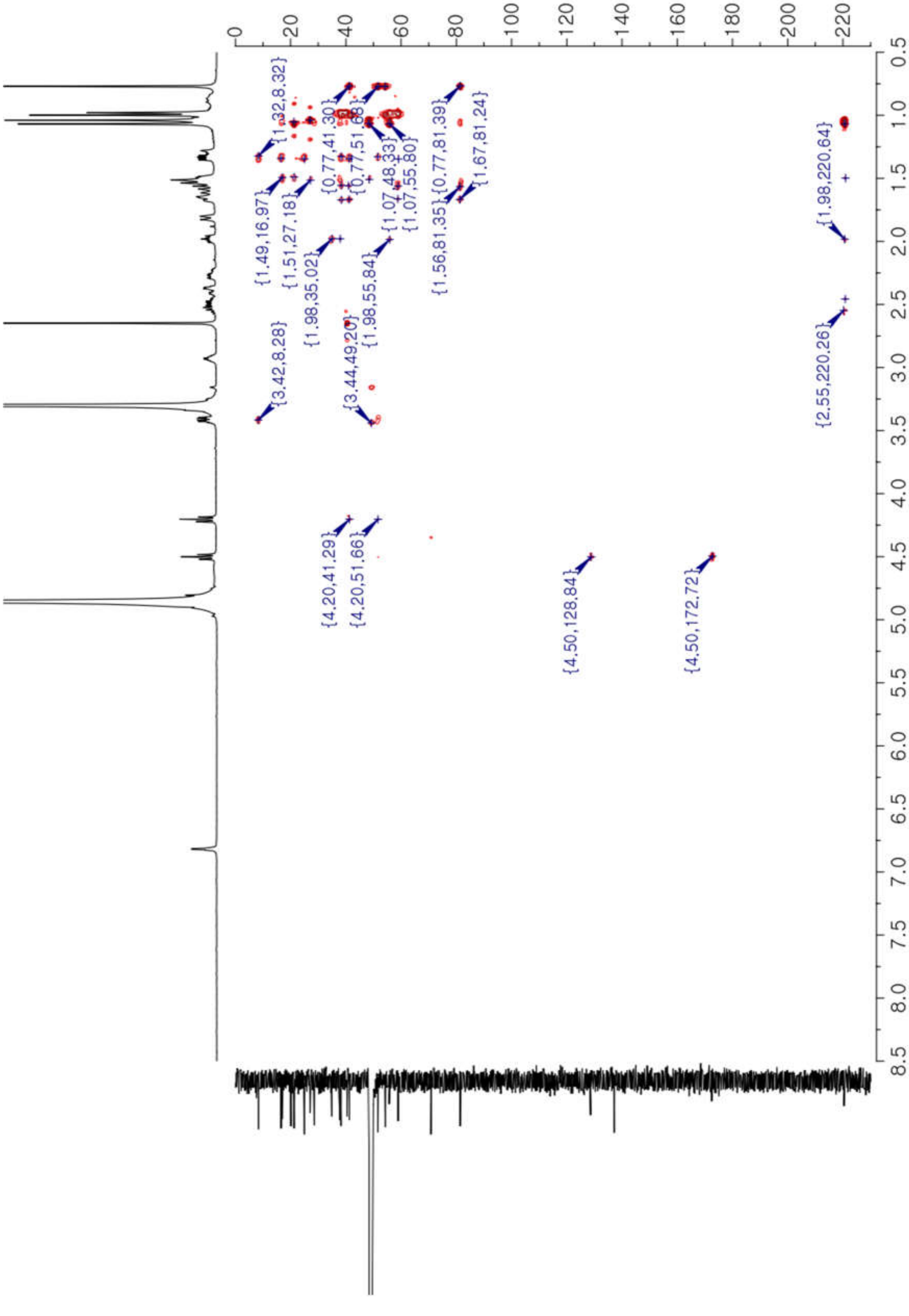

Figure S57. The HMBC (600 MHz, MeOH-d_4) spectrum of **9**

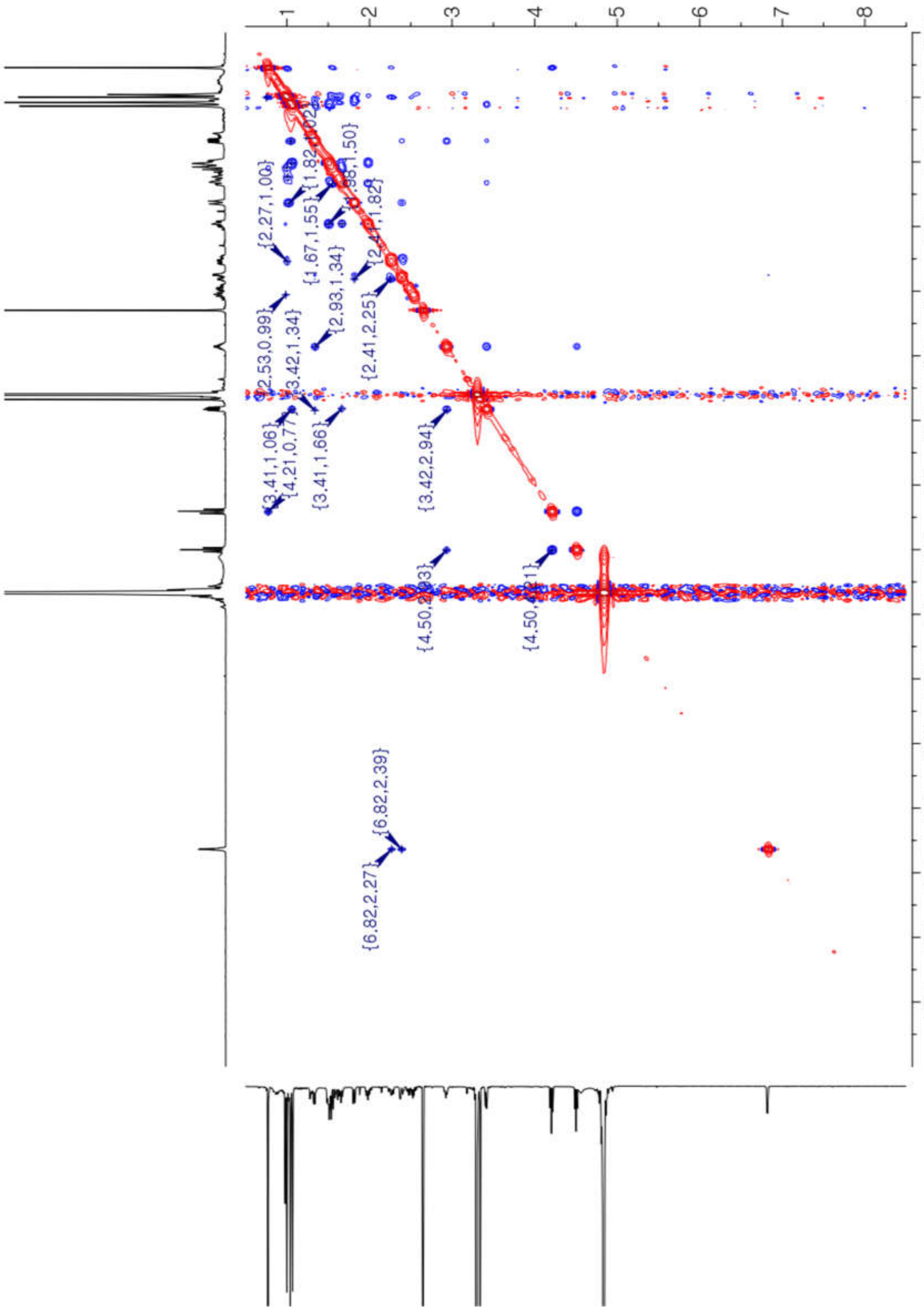

Figure S58. The NOESY (600 MHz, MeOH-d_4) spectrum of **9**

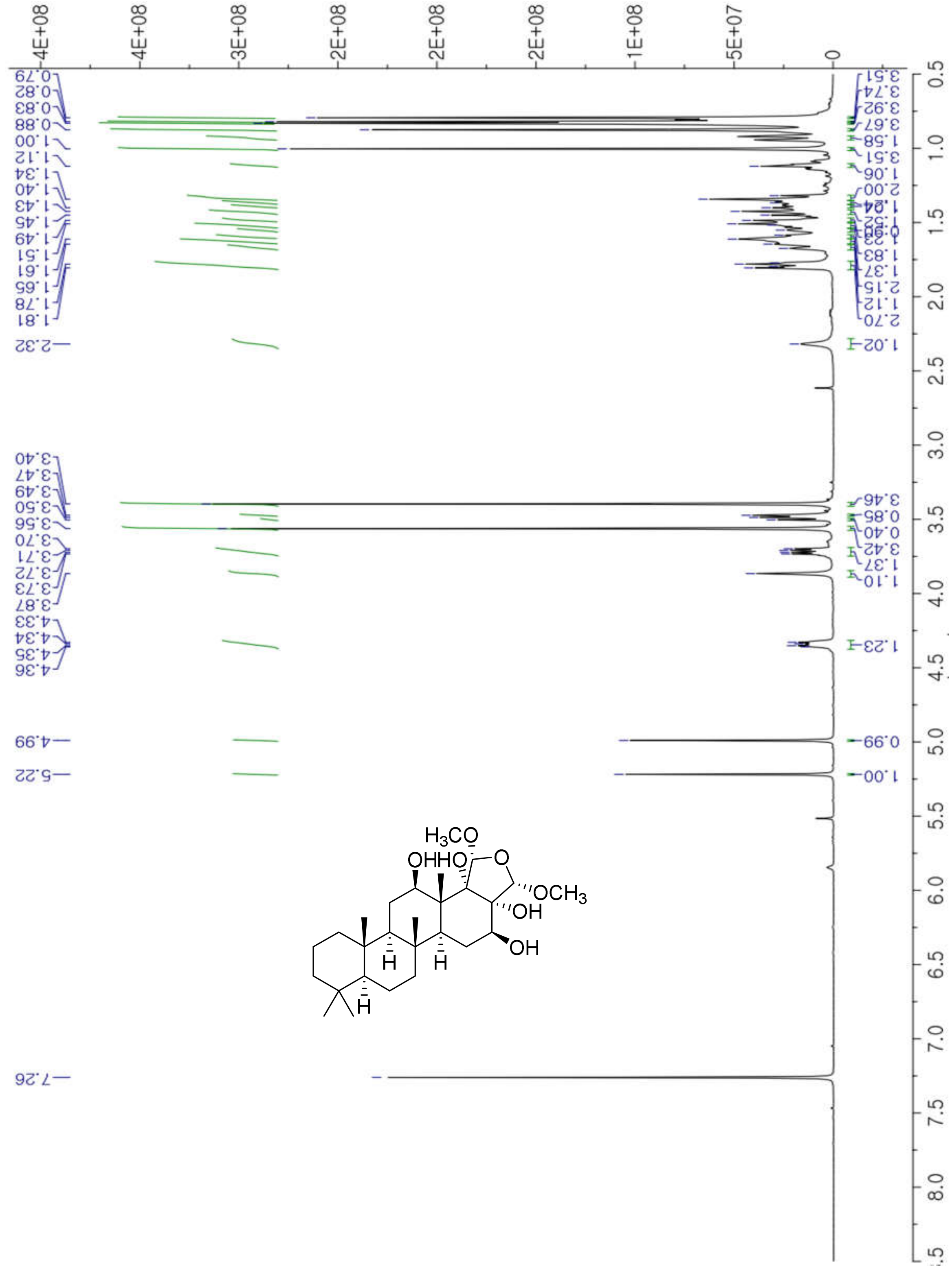

Figure S59. The ^{1}H NMR (600 MHz, $CDCl_3$) spectrum of **10**

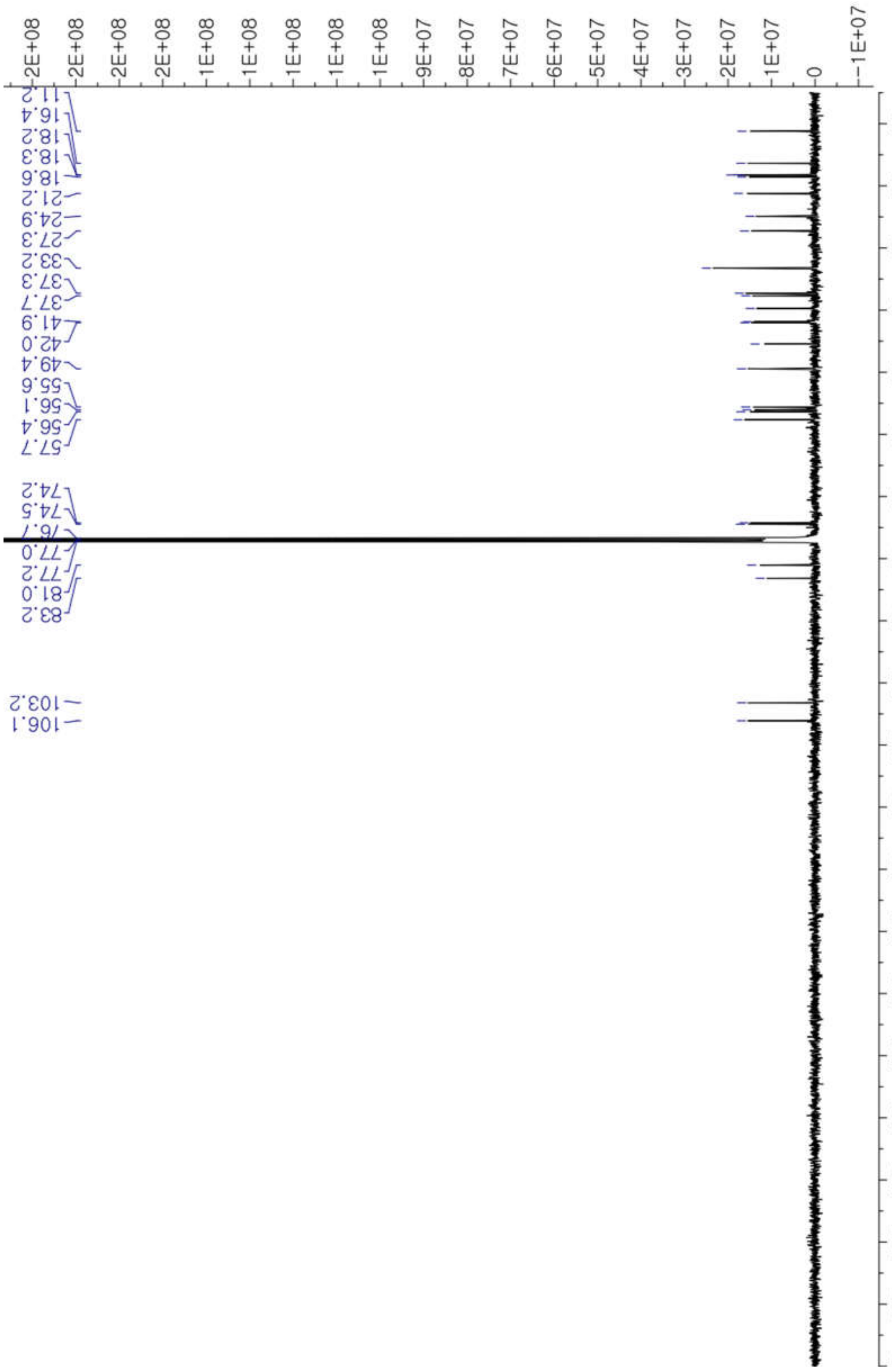

Figure S60. The ^{13}C NMR (150 MHz, $CDCl_3$) spectrum of **10**

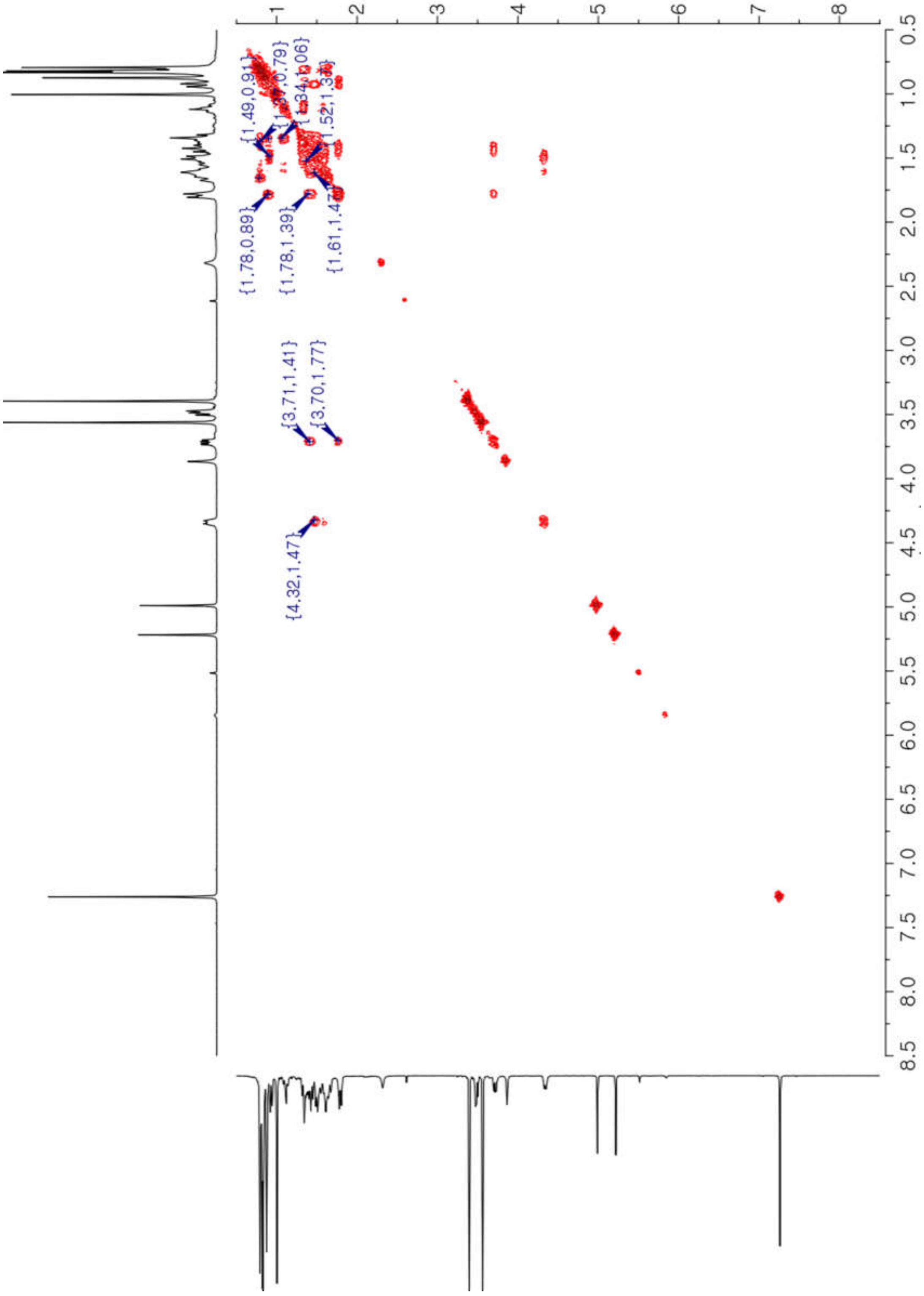

Figure S61. The COSY (600 MHz, $CDCl_3$) spectrum of **10**

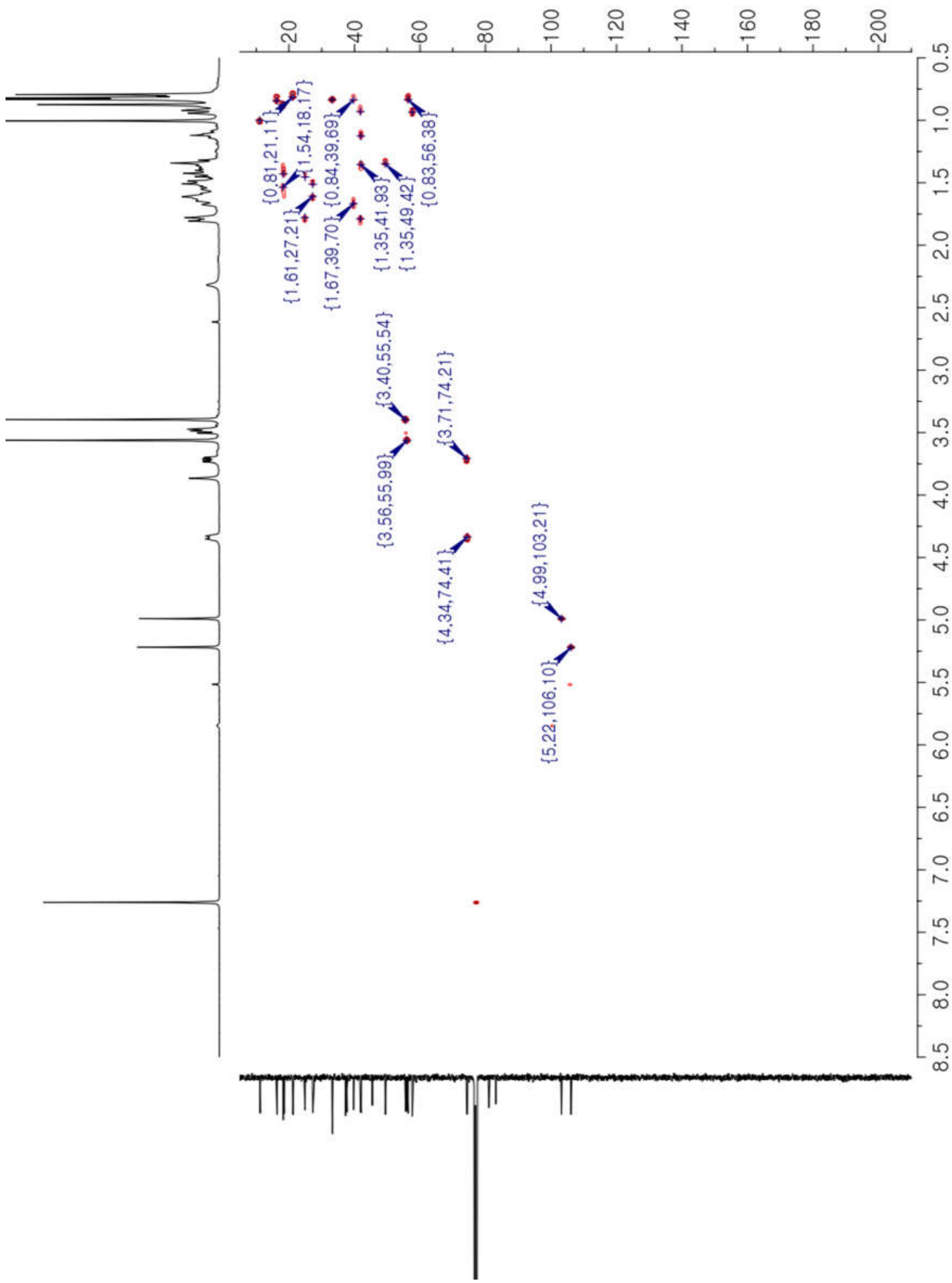

Figure S62. The HSQC (600 MHz, $CDCl_3$) spectrum of **10**

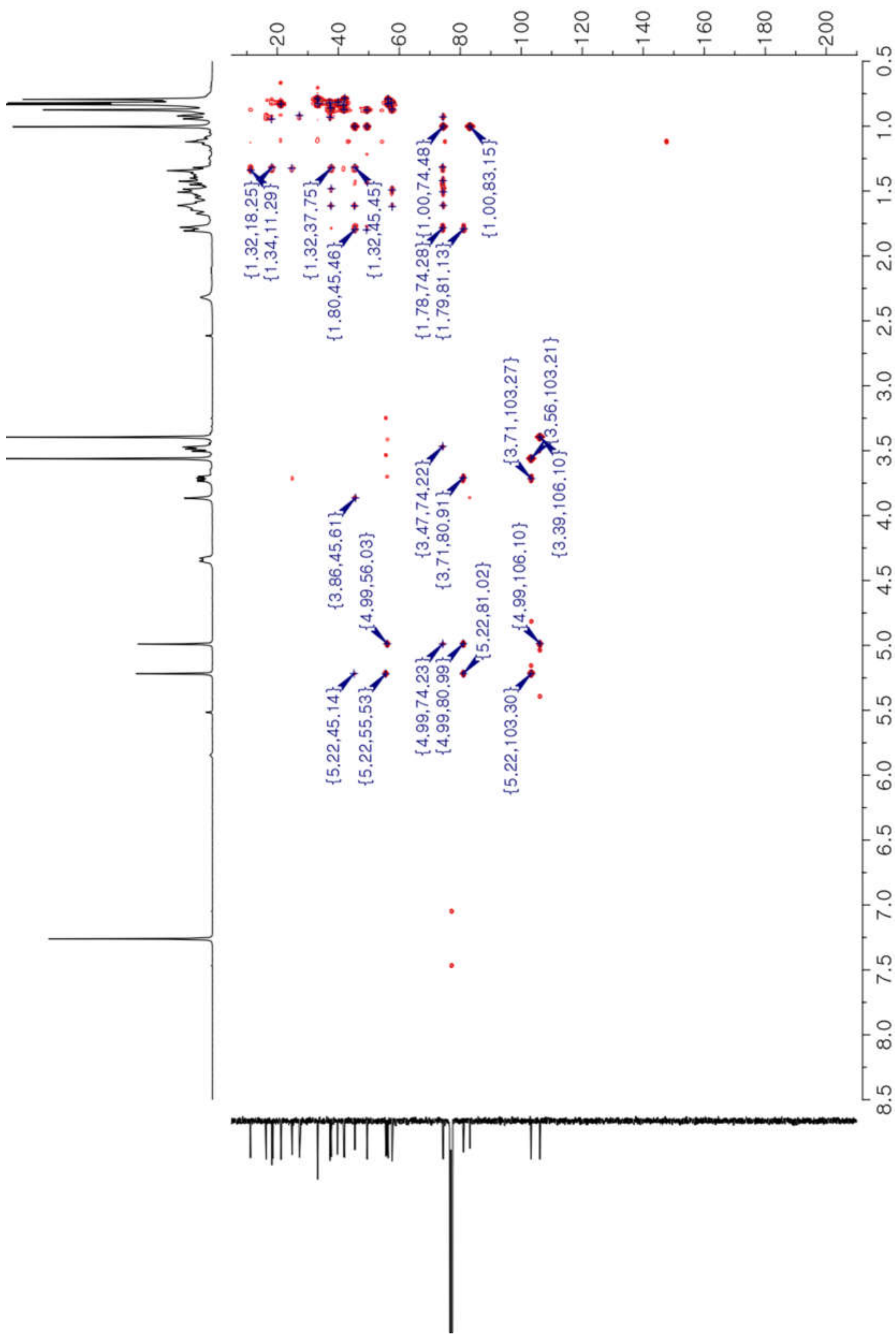

Figure S63. The HMBC (600 MHz, $CDCl_3$) spectrum of **10**

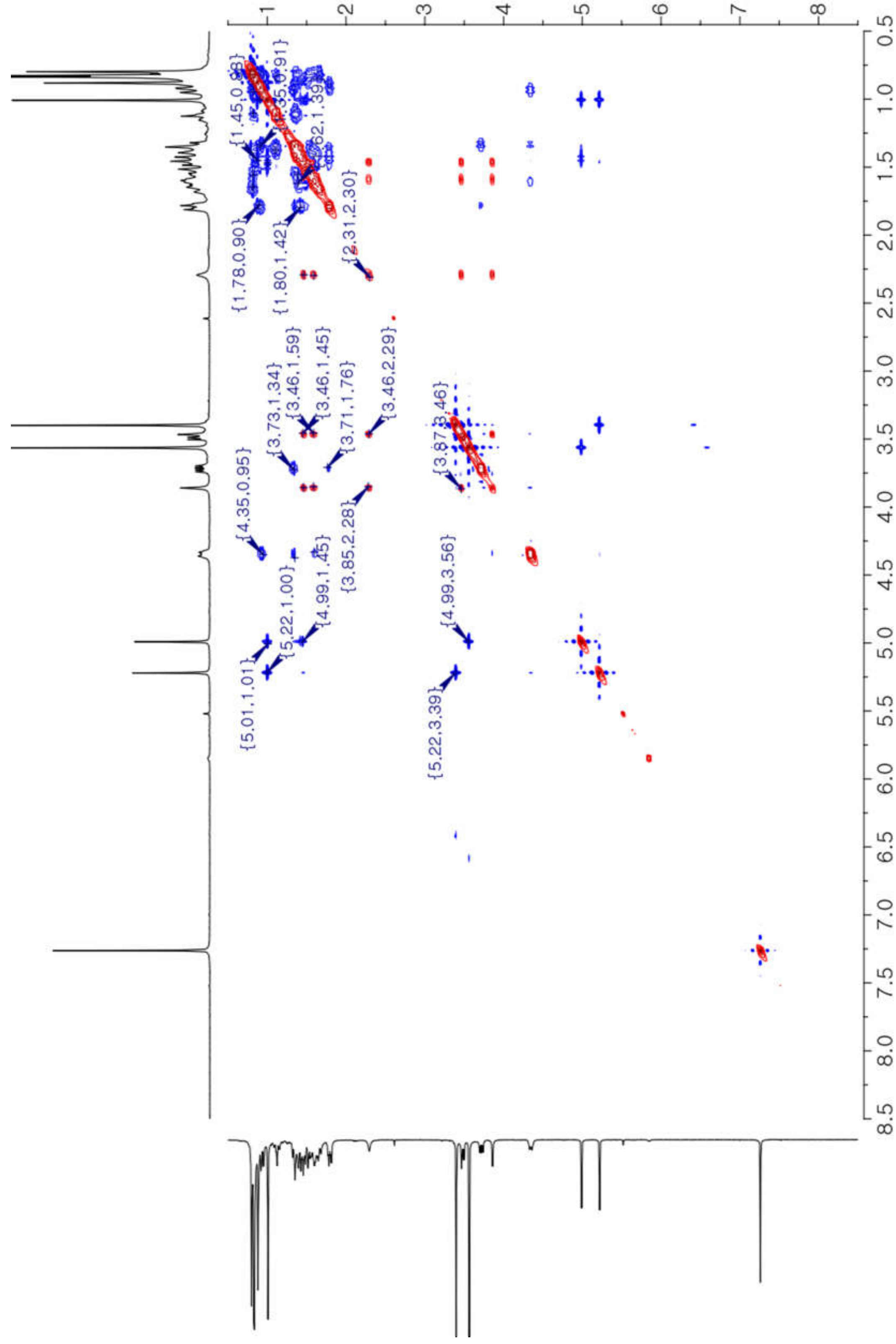

Figure S64. The NOESY (600 MHz, $CDCl_3$) spectrum of **10**

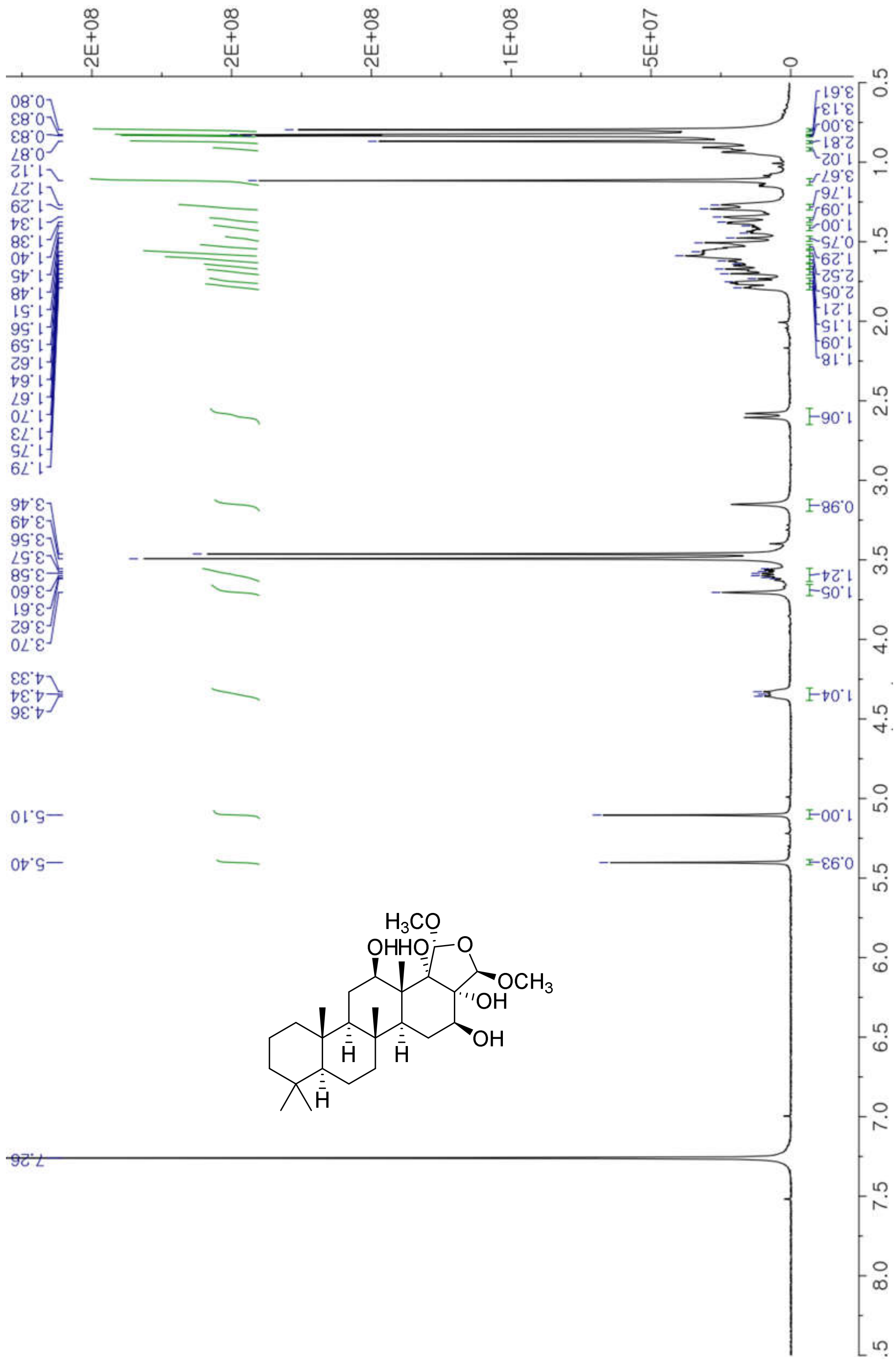

Figure S65. The ^{1}H NMR (600 MHz, $CDCl_3$) spectrum of **11**

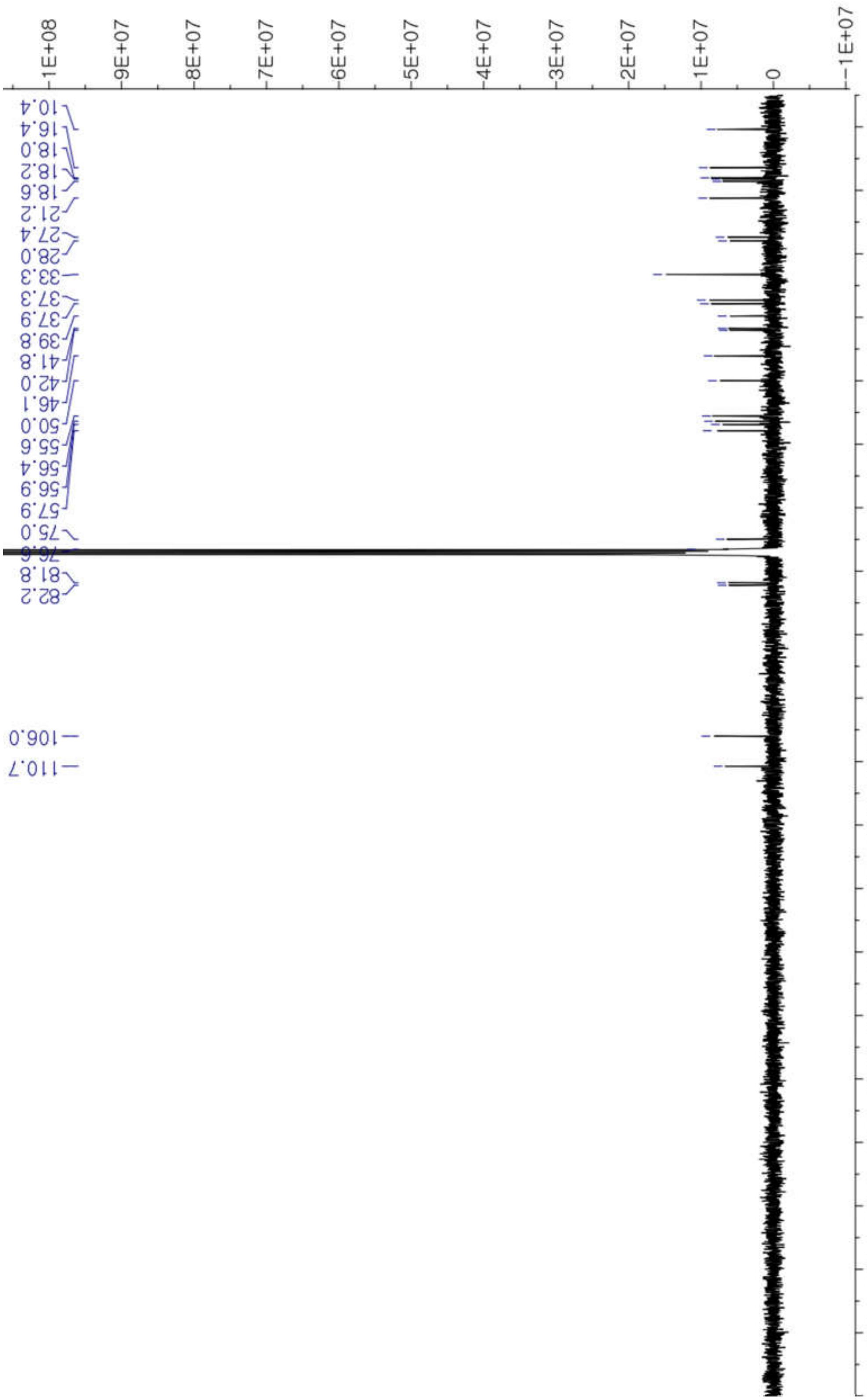

Figure S66. The ^{13}C NMR (150 MHz, $CDCl_3$) spectrum of **11**

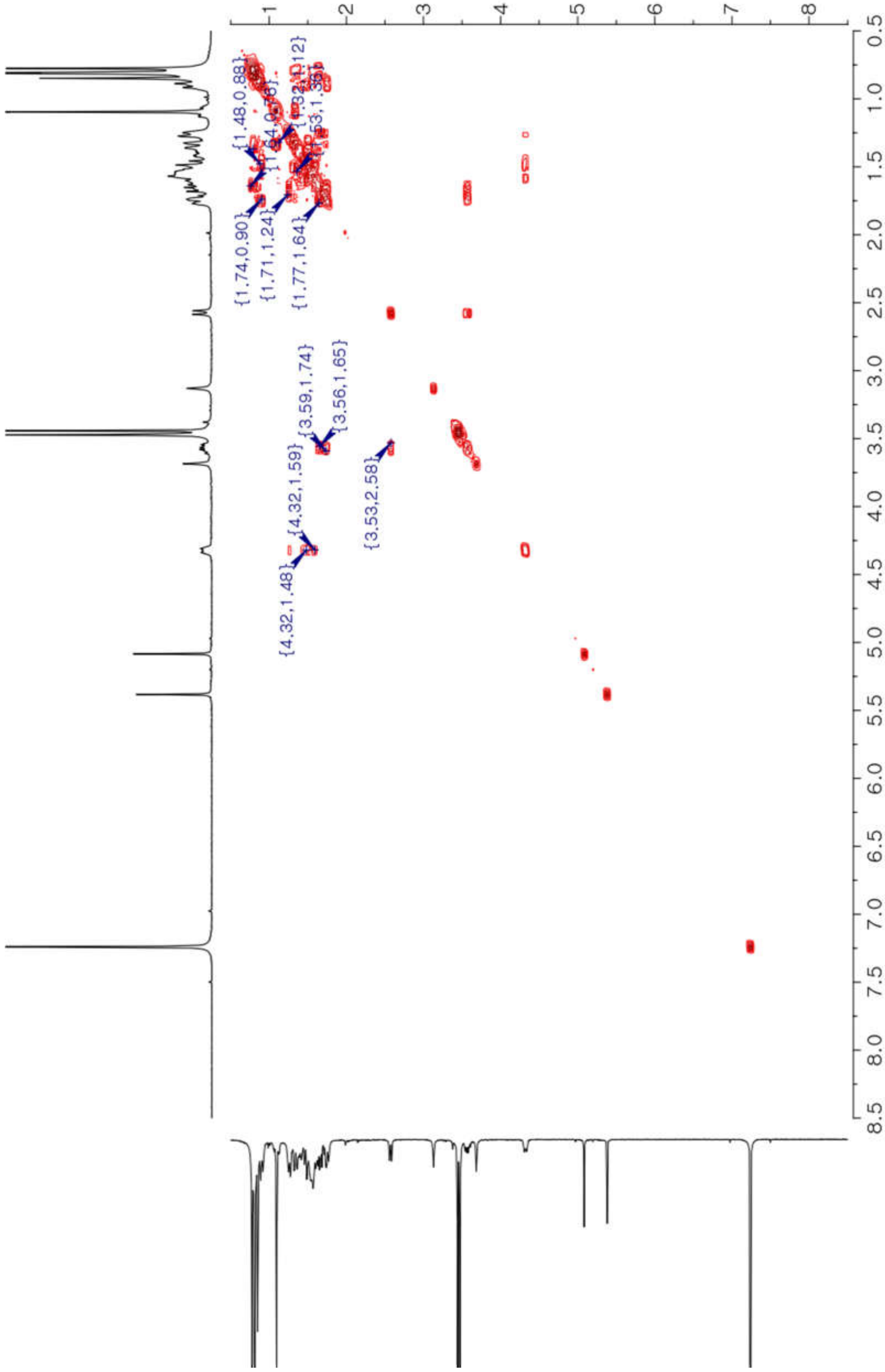

Figure S67. The COSY (600 MHz, $CDCl_3$) spectrum of **11**

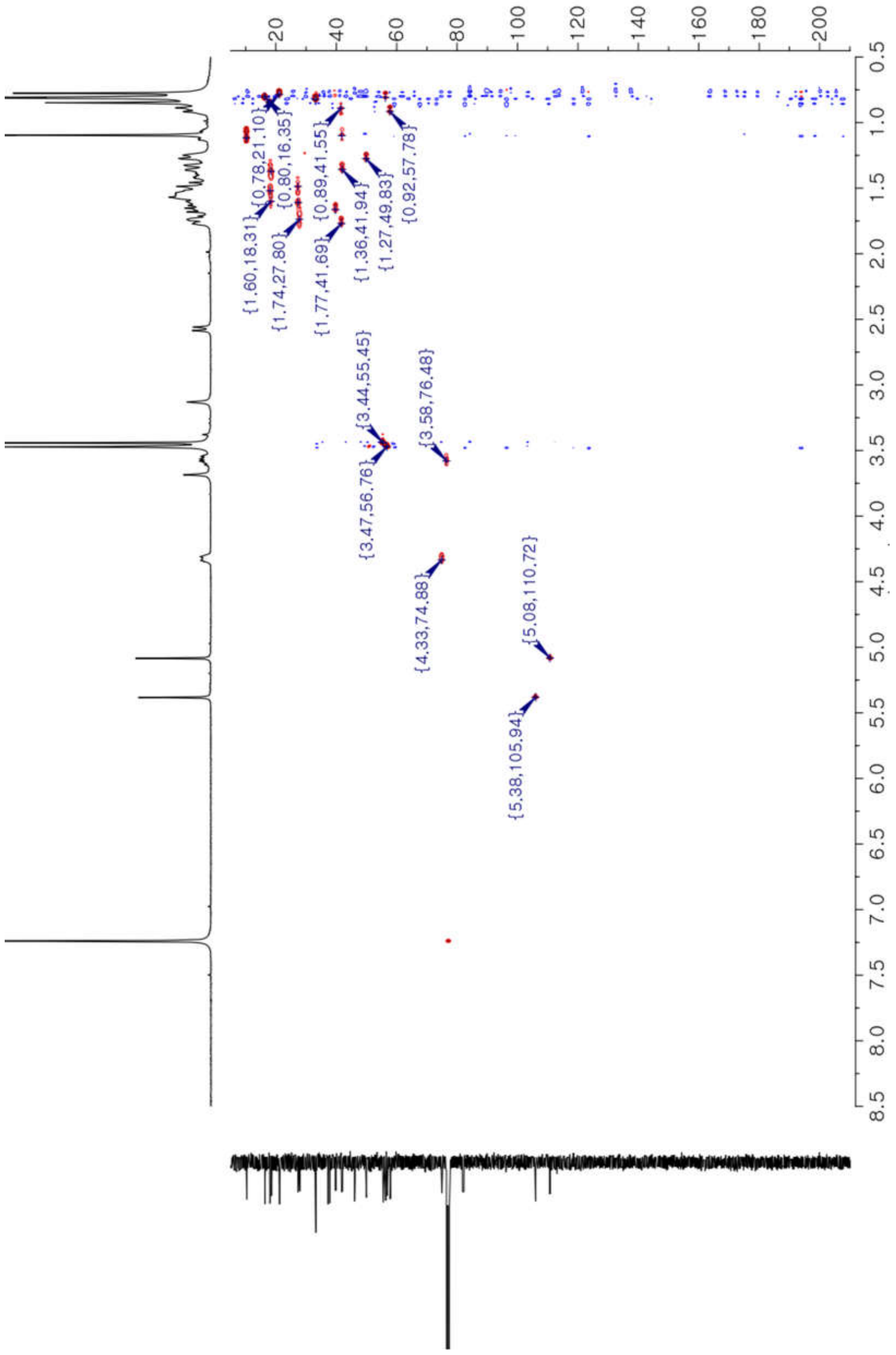

Figure S68. The HSQC (600 MHz, $CDCl_3$) spectrum of **11**

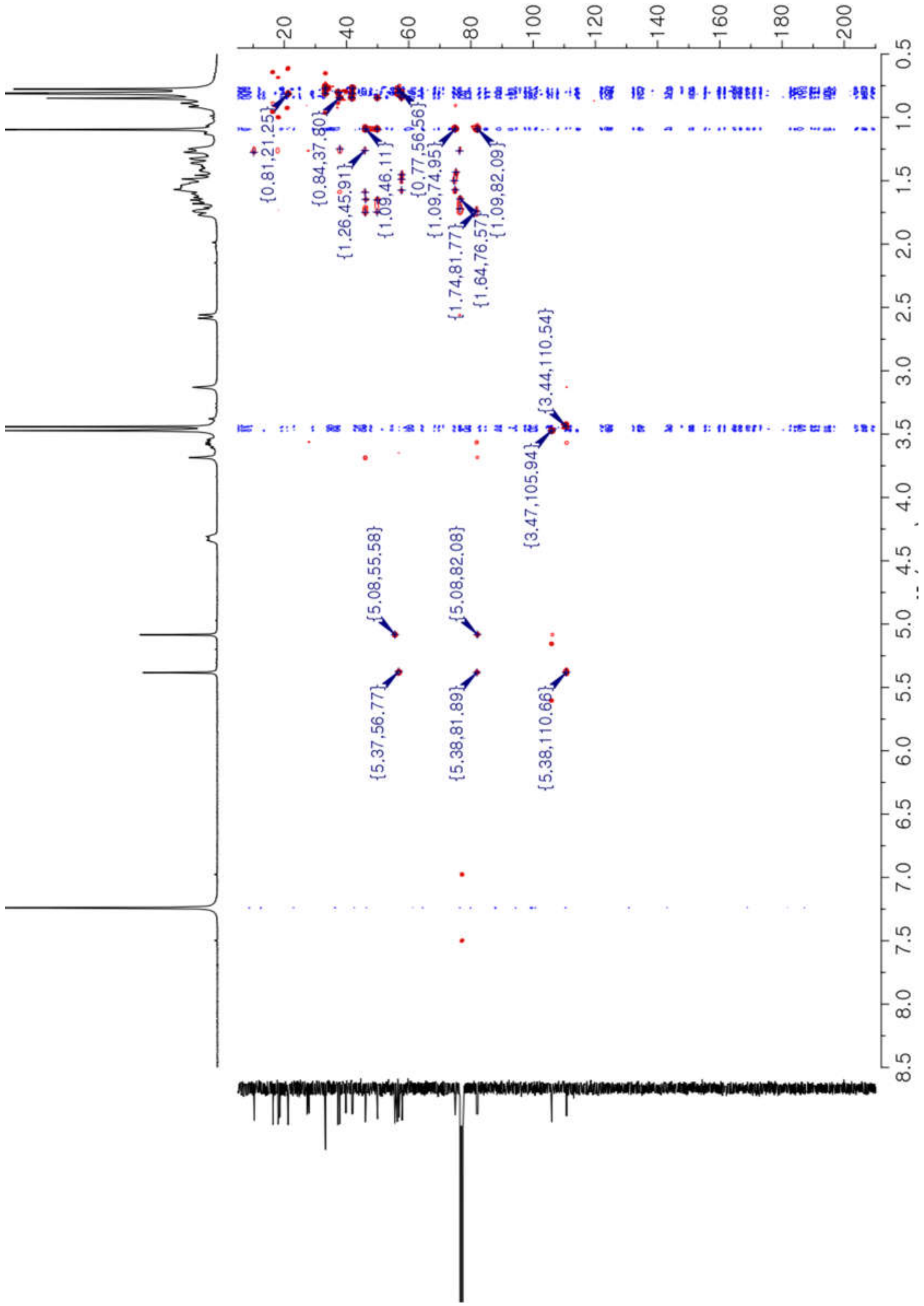

Figure S69. The HMBC (600 MHz, $CDCl_3$) spectrum of **11**

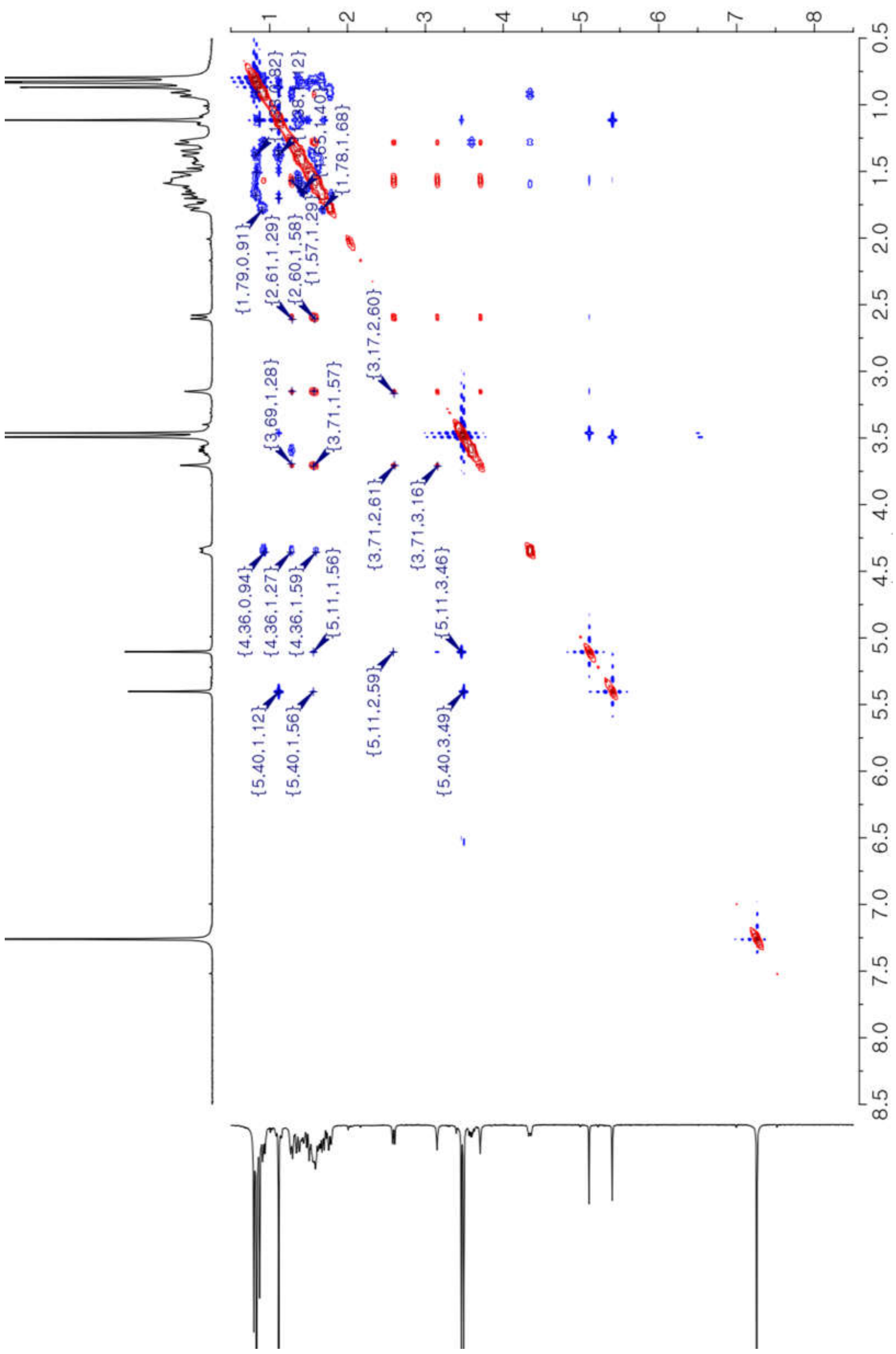

Figure S70. The NOESY (600 MHz, $CDCl_3$) spectrum of **11**

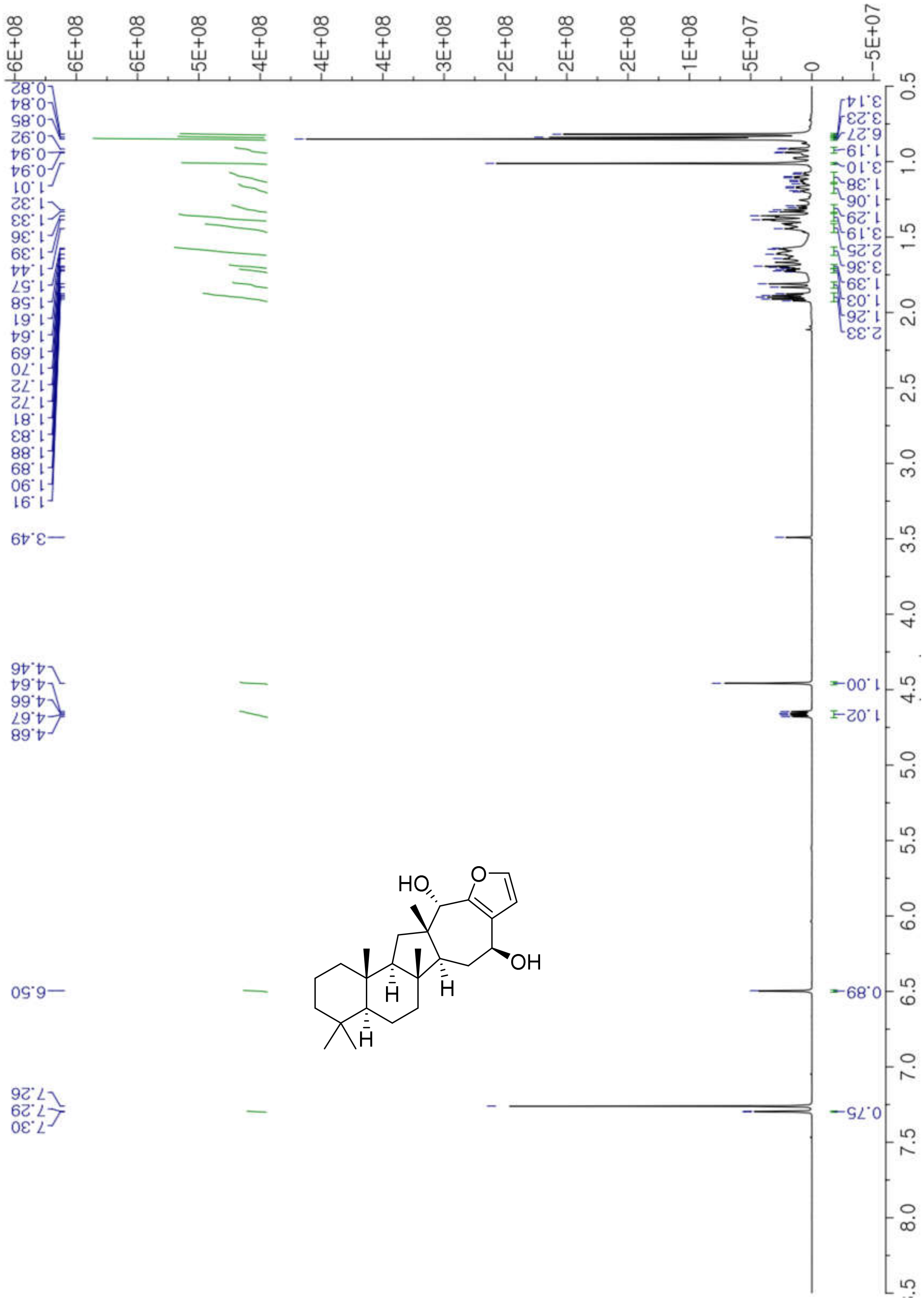

Figure S71. The ^{1}H NMR (600 MHz, $CDCl_3$) spectrum of **12**

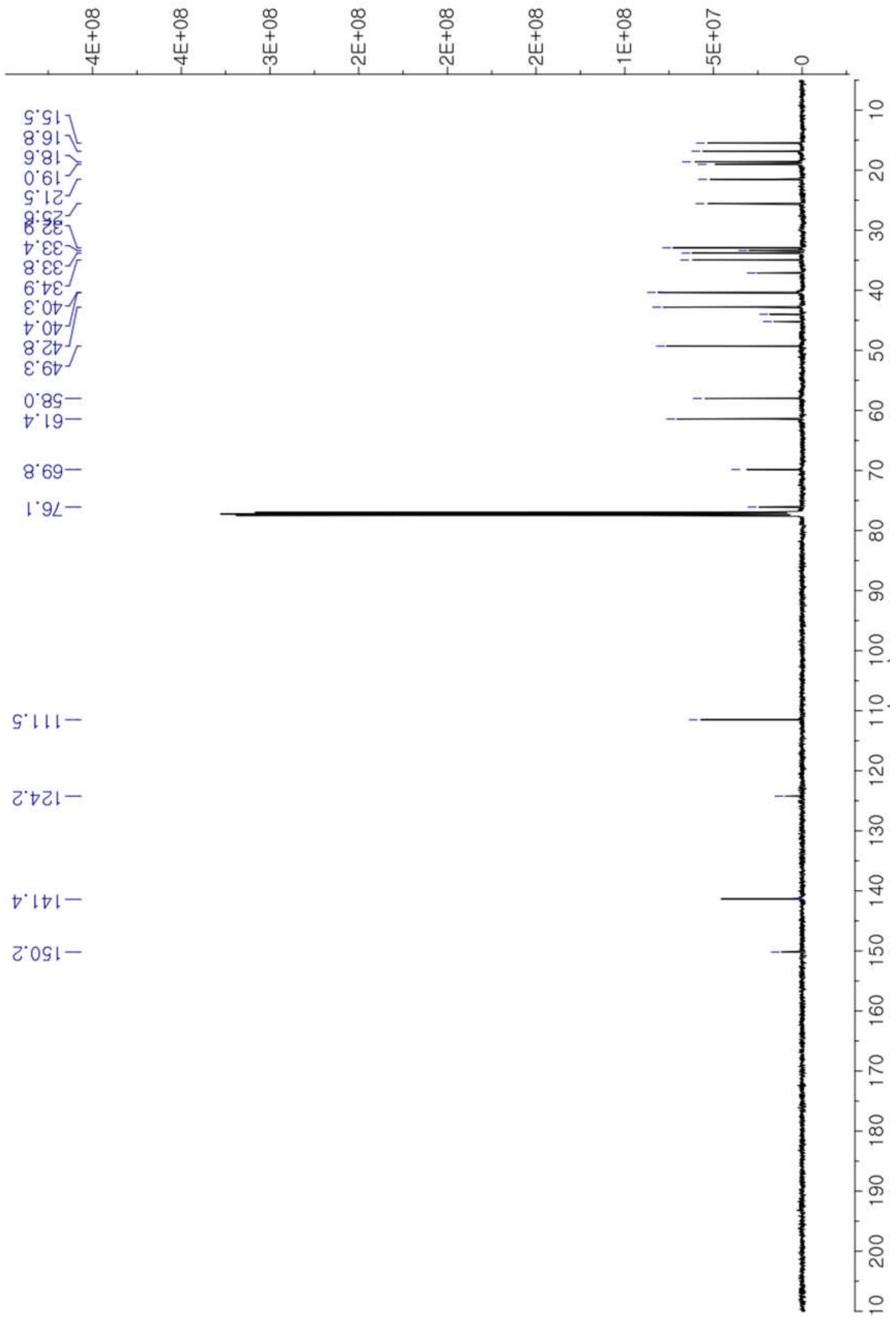

Figure S72. The ^{13}C NMR (150 MHz, $CDCl_3$) spectrum of **12**

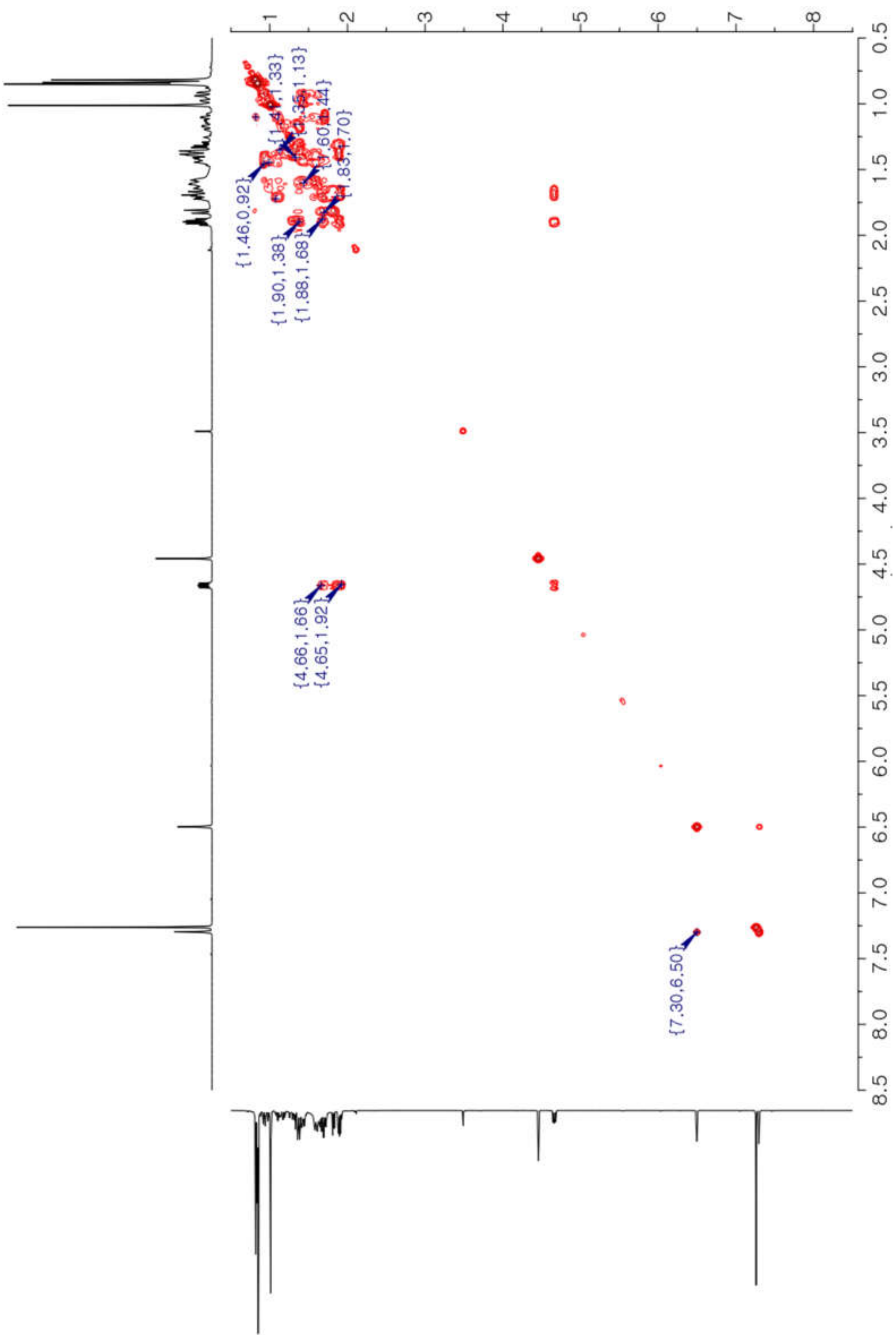

Figure S73. The COSY (600 MHz, $CDCl_3$) spectrum of **12**

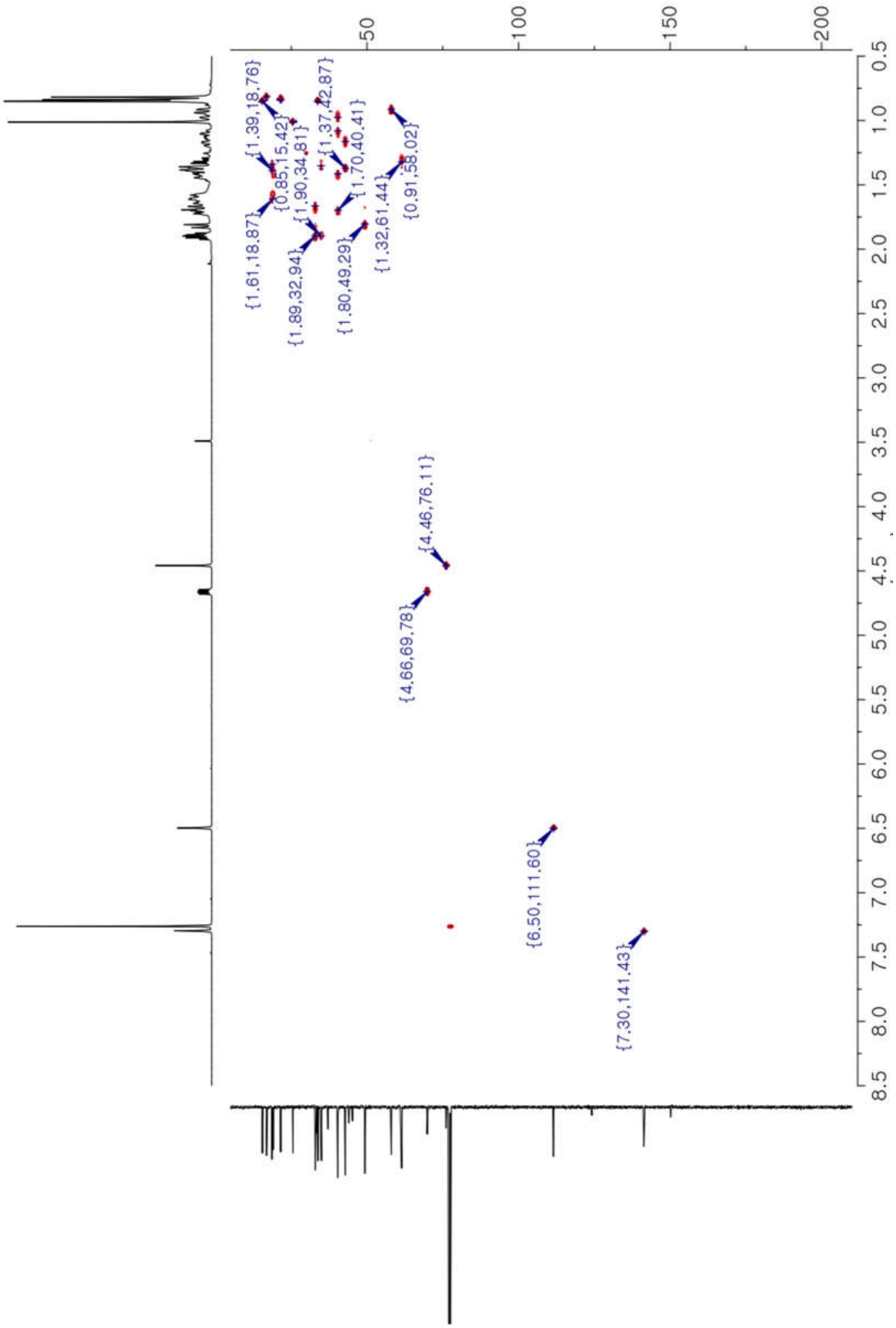

Figure S74. The HSQC (600 MHz, $CDCl_3$) spectrum of **12**

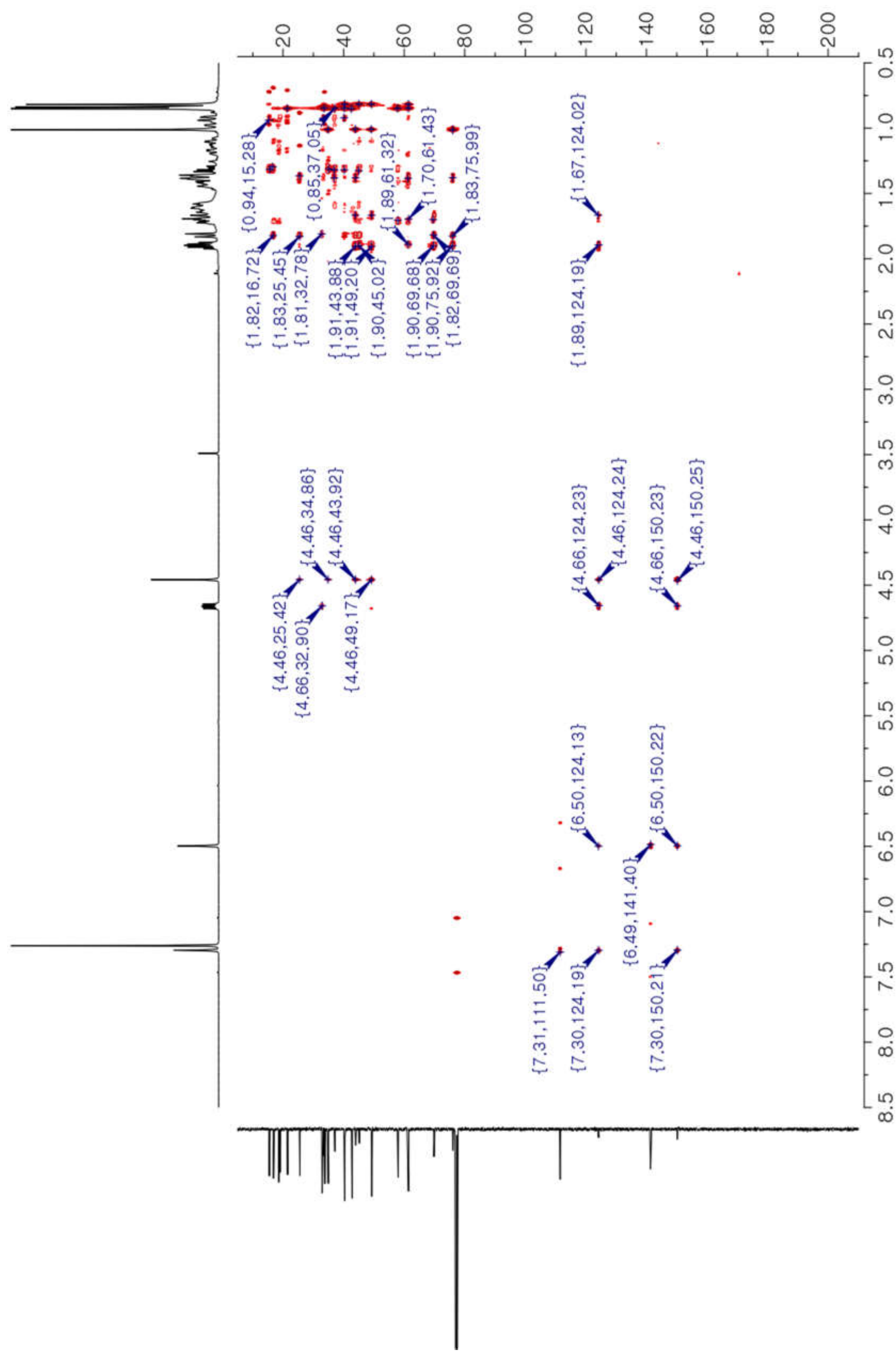

Figure S75. The HMBC (600 MHz, $CDCl_3$) spectrum of **12**

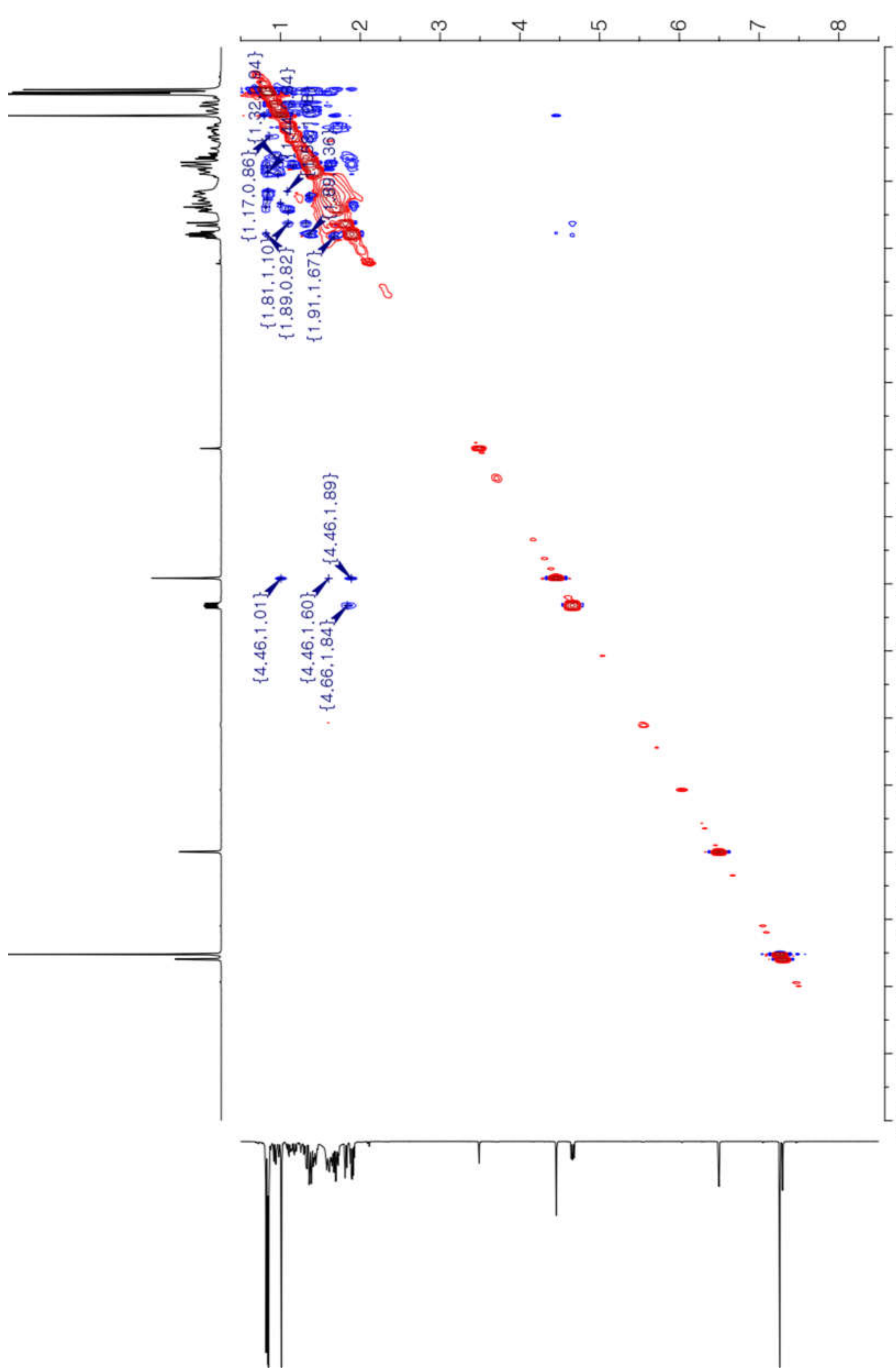

Figure S76. The NOESY (600 MHz, $CDCl_3$) spectrum of **12**

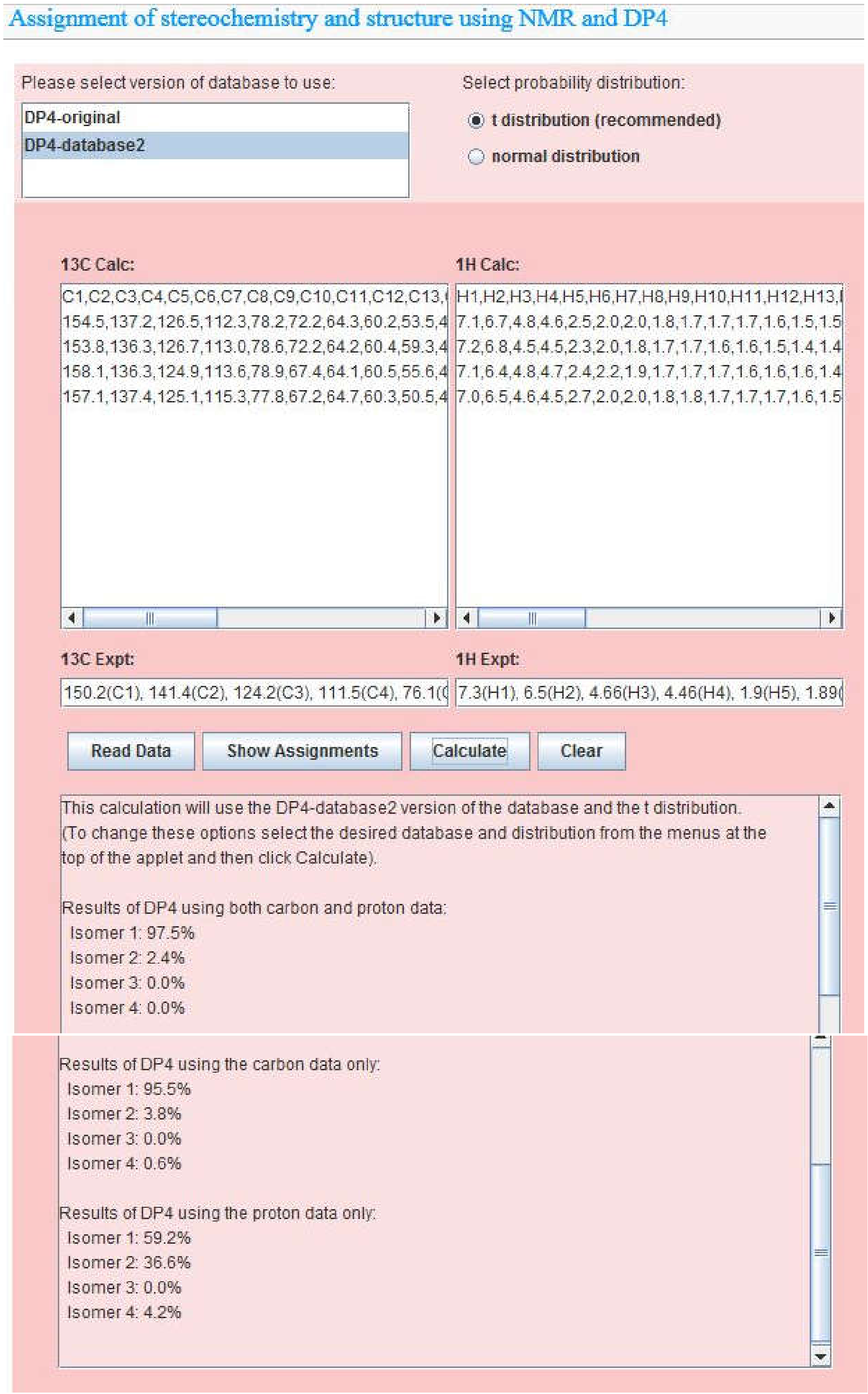

Figure S77. The results of DP4 analyses of **12**.

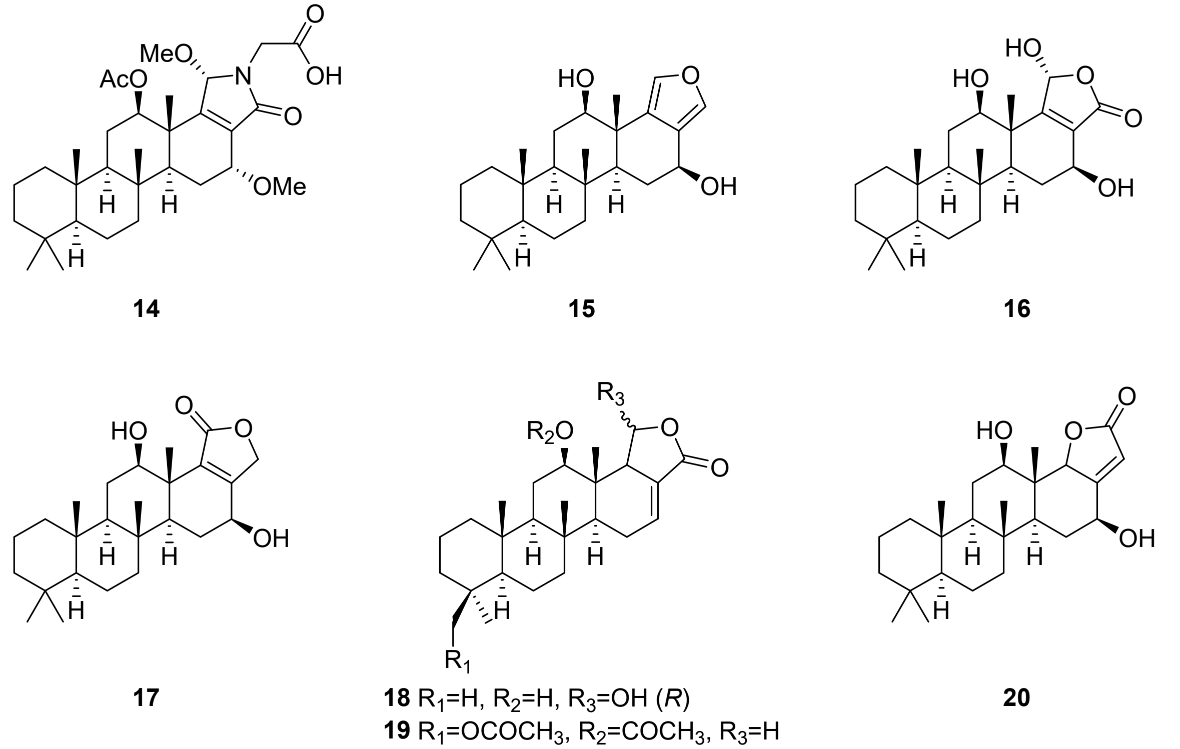

Figure S78. Isolated known compounds from *Hyrtios erectus*

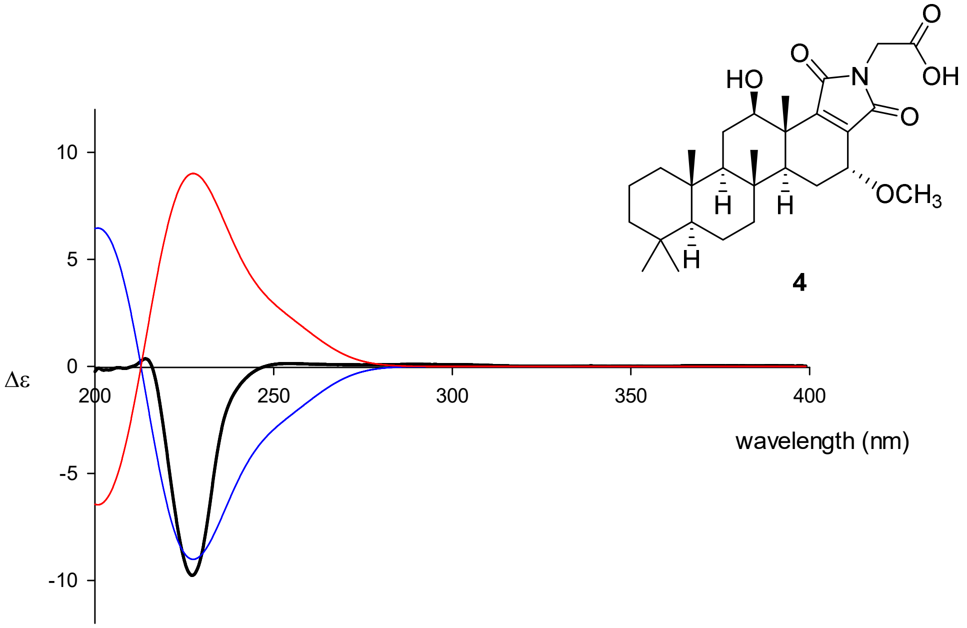

expt for **13**
calc for **13** (5*S*, 8*R*, 9*R*, 10*S*, 12*R*, 13*S*, 14*S*, 16*S*, 19*S*)
calc for **13** (5*R*, 8*S*, 9*S*, 10*R*, 12*S*, 13*R*, 14*R*, 16*R*, 19*R*)

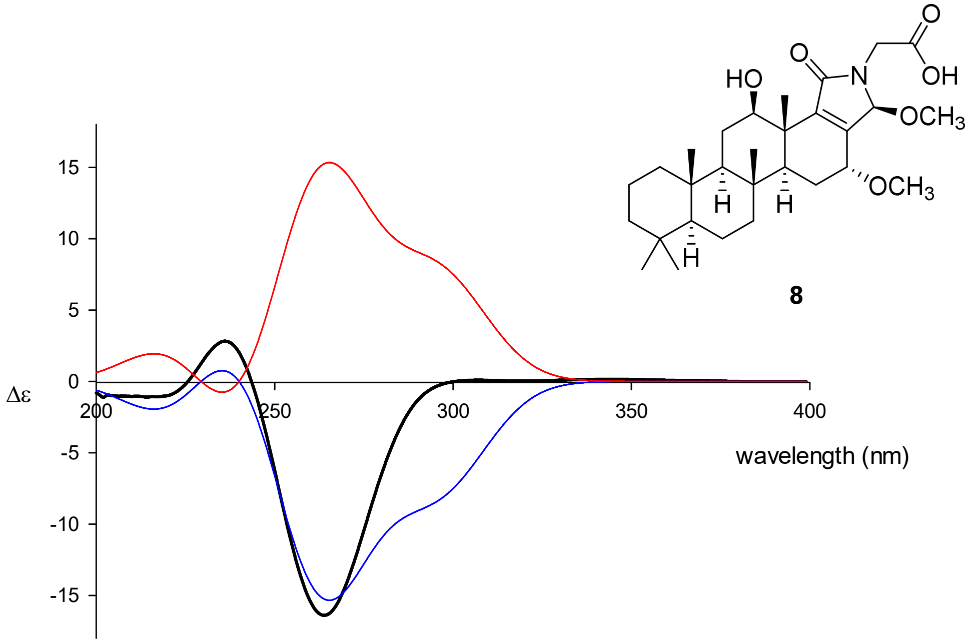

expt for **8**
calc for **8** (5*S*, 8*R*, 9*R*, 10*S*, 12*R*, 13*S*, 14*S*, 16*R*, 20*S*)
calc for **8** (5*R*, 8*S*, 9*S*, 10*R*, 12*S*, 13*R*, 14*R*, 16*S*, 20*R*)

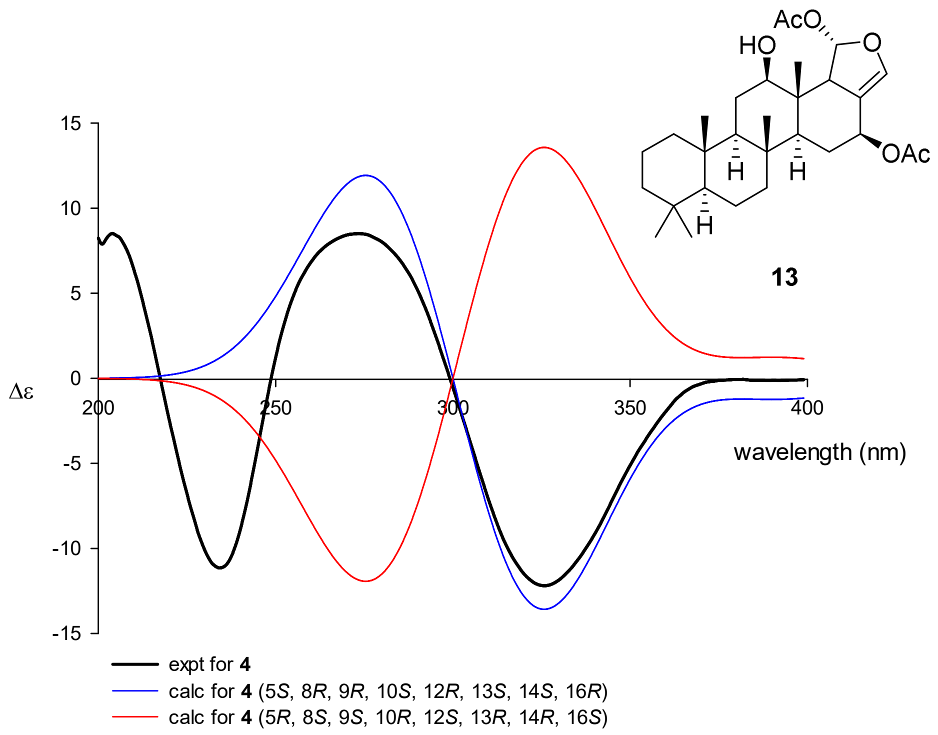

Figure S79. Calculated and experimental ECD spectra of **4**, **8** and **13**
